# NERINE reveals rare variant associations in gene networks across phenotypes and implicates an *SNCA-PRL-LRRK2* subnetwork in Parkinson’s disease

**DOI:** 10.1016/j.xgen.2026.101284

**Published:** 2026-06-22

**Authors:** Sumaiya Nazeen, Xinyuan Wang, Autumn R. Morrow, Ronya Strom, Elizabeth Ethier, Dylan Ritter, Alexander B.H. Henderson, Jalwa Afroz, Christopher S. Cassa, Nathan O. Stitziel, Rajat M. Gupta, Kelvin C. Luk, Lorenz Studer, Vikram Khurana, Shamil R. Sunyaev

**Affiliations:** 1 Department of Biomedical Informatics, Harvard Medical School, Boston, MA, USA; 2 Division of Genetics, Brigham and Women’s Hospital and Harvard Medical School, Boston, MA, USA; 3 American Parkinson’s Disease Association Center for Advanced Research, Harvard Biomarkers Study 2.0 and MyTrial Programs, Division of Movement Disorders, Department of Neurology, Brigham and Women’s Hospital and Harvard Medical School, Boston, MA, USA; 4 Broad Institute of MIT and Harvard, Cambridge, MA, USA; 5 The Center for Stem Cell Biology, Sloan-Kettering Institute for Cancer Research, New York, NY, USA; 6 Department of Neurology, Sean M. Healey & AMG Center for ALS, Massachusetts General Hospital and Harvard Medical School, Boston, MA, USA; 7 Cardiovascular Division, John T. Milliken Department of Medicine, Washington University School of Medicine, St. Louis, MO, USA; 8 Department of Genetics, Washington University School of Medicine, St. Louis, MO, USA; 9 Division of Cardiovascular Medicine, Brigham and Women’s Hospital and Harvard Medical School, Boston, MA, USA; 10 Department of Pathology and Laboratory Medicine, Perelman School of Medicine at the University of Pennsylvania, Philadelphia, PA, USA; 11 Aligning Science Across Parkinson’s (ASAP) Collaborative Research Network, Chevy Chase, MD, USA; 12 Harvard Stem Cell Institute, Cambridge, MA, USA

**Keywords:** network-based rare variant association, multivariate statistical genetics, complex disease genetics, Parkinson's disease, *α-synuclein/prolactin* stress response, neuronal Parkinson's model, type 2 diabetes, coronary artery disease, myocardial infarction, breast cancer

## Abstract

Studying the genetic basis of human phenotypes involves two primary strategies. Model-system experiments generate interpretable gene networks but do not establish relevance to human disease. In contrast, statistical genetics identifies variant- and gene-level associations but cannot test mechanistic models. Here, we bridge these approaches by introducing NERINE, a hierarchical model-based rare variant association test that incorporates gene network topology while remaining robust to network inaccuracies. NERINE supports analysis of networks from established pathway databases and model-system screens. A comprehensive search across pathway databases reveals associations for breast cancer, cardiovascular diseases, and type 2 diabetes not detected by single-gene tests. Applied to experimental screen-derived networks in Parkinson’s disease (PD), NERINE highlights autophagy-, vesicle-trafficking-, and protein-homeostasis-related gene modules. Genome-scale CRISPR interference (CRISPRi) screening in human neurons and NERINE converge on *PRL*, revealing an intraneuronal *α-synuclein*/*prolactin* stress response that may impact resilience to PD.

## Highlights

• A hierarchical model for rare variant association integrating gene network topology
• NERINE significantly improves association power in noisy networks
• We identify gene networks associated with several human diseases
• NERINE and CRISPRi screen link *α-synuclein*/*prolactin* stress response to Parkinson’s

## Graphical abstract

**Figure:**
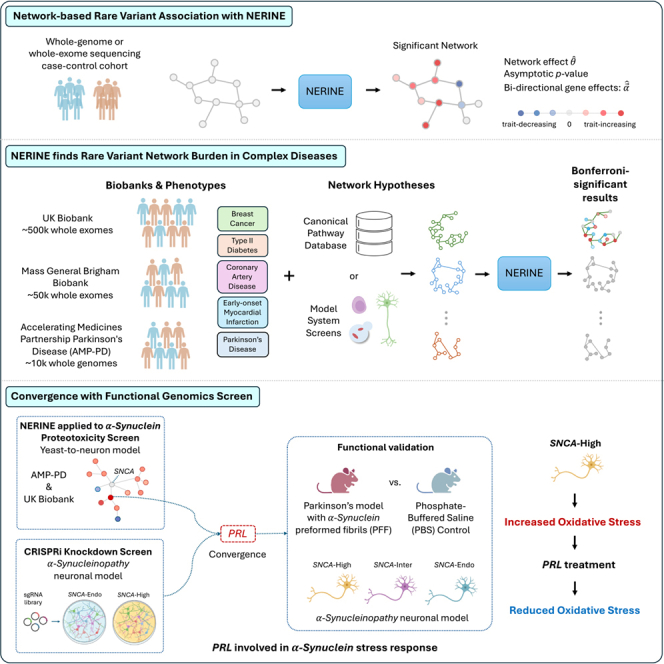

## Teaser

In this article, Nazeen, Khurana, Sunyaev, and colleagues present NERINE, a statistical test that links rare genetic variation in gene networks to human diseases, identifying new disease associations in biobanks. Coupled with CRISPRi screening in neuronal models, this framework reveals an *α-synuclein*/*prolactin* stress-response axis in Parkinson’s disease.

## Introduction

Advancements in high-throughput screening have resulted in large-scale datasets that capture crucial biological knowledge, often represented as gene and protein interaction networks.^1^^,^^2^^,^^3^^,^^4^^,^^5^^,^^6^^,^^7^ Some networks represent metabolic or signaling pathways, while others capture regulatory interactions, physical protein-protein interactions (PPIs), or protein-DNA associations. More abstractly, genetic interactions, such as co-essentiality, reflect dependencies in the contributions of genes to cellular fitness and phenotypes. As a result, gene networks serve as fundamental frameworks for understanding polygenic trait variation, including complex disease mechanisms, enabling researchers to formulate testable biological hypotheses about human phenotypes.^8^^,^^9^^,^^10^

Establishing causal links between gene networks and phenotypes requires direct evidence from human genetics. Rare protein-coding variants offer a straightforward link, as they directly impact gene function^11^^,^^12^; pathway-level associations reveal new insights into causal disease mechanisms.^13^^,^^14^^,^^15^^,^^16^ However, existing tests do not leverage network topology.^14^^,^^15^^,^^16^^,^^17^^,^^18^^,^^19^^,^^20^^,^^21^^,^^22^^,^^23^^,^^24^^,^^25^ They treat pathways as either a single entity (“mega-gene”) or a simple collection of genes (“bag of genes”) and ignore the fact that not all genes in a pathway contribute equally to a phenotype.

To address this gap, we present NERINE (network-based rare variant enrichment), a rare variant association testing framework that integrates information about genes and their interactions into a hierarchical model. NERINE offers several advantages. First, it evaluates and prioritizes competing network topologies and the experimental assays that define them by their relevance to human phenotypes. Second, it improves statistical power by aggregating rare variants across gene modules with defined topologies and estimates gene-level directional effects on phenotypes. Third, it improves biological specificity by utilizing experimentally derived topologies. Fourth, it accommodates network inaccuracies (e.g., false positive “nodes”). This framework enables integrative strategies in which functional screens generate network hypotheses for association testing with human phenotypes, which can subsequently be refined and validated through targeted experiments, thereby synergizing genetic discovery and mechanistic understanding.

We demonstrate two avenues to apply NERINE: (1) testing database-derived pathway modules in the UK Biobank (UKBB) and Mass General Brigham Biobank (MGBBB) for associations with breast cancer (BRCA), type 2 diabetes (T2D), coronary artery disease (CAD), and early-onset myocardial infarction (MI) and (2) analyzing networks generated from experimental screens targeting core Parkinson’s disease (PD) pathologies—*α-synuclein* (*αS*) proteotoxicity and dopaminergic (DA) neuron survival in UKBB and AMP-PD cohorts, where gene-level tests remain underpowered.^26^^,^^27^ For common diseases, NERINE uncovers associations in estrogen receptor regulation (BRCA), adipogenesis (T2D), and non-lipid inflammatory response pathways (CAD and MI). For PD, we identified associations in networks linked to autophagy regulation (*HMGB1-OPTN-USP10* module) and vesicle trafficking and protein homeostasis (*LRRK2-PRL-SNCA* module).

Notably, an association identified by NERINE between rare damaging missense variants at the *PRL* locus encoding *prolactin* (*Prl*) and PD risk converges with an independent genome-scale functional screen in a CRISPR interference (CRISPRi)-induced synucleinopathy cortical neuron (CiS-CN) model designed to study *αS* toxicity modifiers.^28^ Subsequent functional validation using the CiS-CN model and a chronic fibrillar *αS* mouse model^29^ supports a hitherto unknown intraneuronal role of *PRL* in the *αS* stress response in PD. This underscores the potential of experimental screens and human genetics as mutually reinforcing methodologies to uncover disease mechanisms.

## Results

### Modeling rare variant burden in gene networks incorporating edge geometry

**Figure 1:**
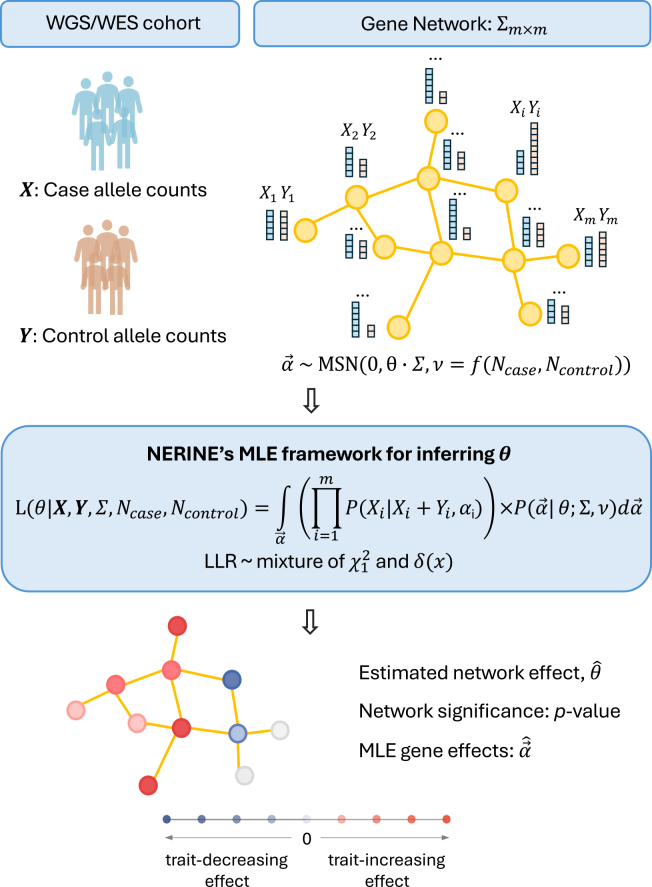
Overview of NERINE: A rare variant association test leveraging gene network topology NERINE’s hierarchical framework for testing rare variant burden aggregated across a gene network for binary traits. Inputs include allele counts in cases (***X***) and controls (***Y***) from WGS or WES datasets and a gene network encoded by a symmetric positive semidefinite matrix, Σ_m×m_. Σ can represent diverse biological relationships (e.g., physical or genetic interactions, co-expression, co-essentiality, and pathway membership). Gene effects $\left(\vec{\alpha }\right)$ are modeled with a multivariate skew-normal distribution parameterized by Σ, network effect *θ*, and case-control skew *ν*. This parameterization allows network genes to have either zero effect or varying degrees of trait-increasing and -decreasing effects. *θ* is inferred using a maximum likelihood estimation (MLE) framework where the likelihood, *L*, is an integral over the product of two terms—(1) the product of the per-gene conditional probability of observing allele count in cases (*X*_*i*_) given the allele count in the overall cohort (*X*_*i*_ + *Y*_*i*_) in the network and (2) the probability of observing a specific combination of gene effects determined by *θ* and Σ. NERINE performs nested hypothesis testing, with the log likelihood ratio (LLR) being the test statistic, and provides an asymptotic *p* value from the mixture of the delta function, *δ*(*x*), and a chi-squared distribution with one degree of freedom $\chi_{1}^{2}$. It also estimates the most likely gene effects ($\hat{\vec{\alpha }}$) under the estimated $\hat{\theta }$ (STAR Methods).

We present NERINE, a statistical framework for assessing the cumulative effect of rare variants in gene networks on dichotomous phenotypes (Figure 1). NERINE incorporates information on network vertices (genes) and edges (interactions) into a parametric model and is robust to the presence of uninformative genes in the network (see Table S1 for comparison with existing methods). Various data types represent interactions between genes and proteins. Interactions differ in terms of relationship types (from protein complexes to sets of co-expressed genes to protein-DNA or protein-RNA interactions) and scale (from large interaction networks to pathways of just a dozen genes). We encode gene-gene relationships using a symmetric positive-semidefinite matrix, Σ.

We model gene effect sizes ($\vec{\alpha }$) as draws from a multivariate skew-normal distribution $\vec{\alpha }\;∼\;MSN\left(\vec{\alpha }\;|0,\theta \cdot \Sigma ,\nu \right)$ to capture the biological expectation that functionally related genes exhibit correlated effect sizes (when non-zero) and correlated probabilities of having no phenotypic effect (STAR Methods). Here, *θ* reflects the effect of the network on the phenotype and is the object of inference, and ν encodes the case-control skew. This parameterization allows gene effect sizes to vary in both magnitude and direction while permitting some to be zero (in case of noisy networks).

NERINE infers *θ* using the maximum likelihood estimation (MLE) framework, where the likelihood is given by $L\left(\theta |X,Y,\vec{\alpha },\Sigma ,N_{case},N_{control}\right)=\int \left(\prod_{i}P\left(X_{i}|X_{i}+Y_{i},\alpha_{i}\right)\right)P\left(\vec{\alpha }|\theta ;\Sigma ,\nu \right)d\vec{\alpha }$. To compute L efficiently, we make several modeling assumptions for rare variant counts in network genes in cases (***X***) and controls (***Y***), as well as for gene effects $\vec{\alpha }$. We approximate L as a weighted sum over multivariate quadrature points in the domain of integration, achieved through a lookup table with pruning (STAR Methods). NERINE performs nested hypothesis testing (*θ* = 0 vs. *θ* > 0) using the log likelihood ratio (LLR) statistic (STAR Methods). The asymptotic distribution of LLR arises from a weighted mixture of a point mass at zero and a chi-squared distribution with one degree of freedom,^30^^,^^31^ which we confirm through null simulations (*θ* = 0) using canonical pathways (Figures S1 and S2) and artificial topologies (Figure S3). When the alternative hypothesis is true, NERINE estimates a non-zero network effect ($\hat{\theta })$, provides asymptotic *p* values, and predicts the most likely gene effects under $\hat{\theta }$, but it does not provide gene-level *p* values (STAR Methods).

**Figure 2:**
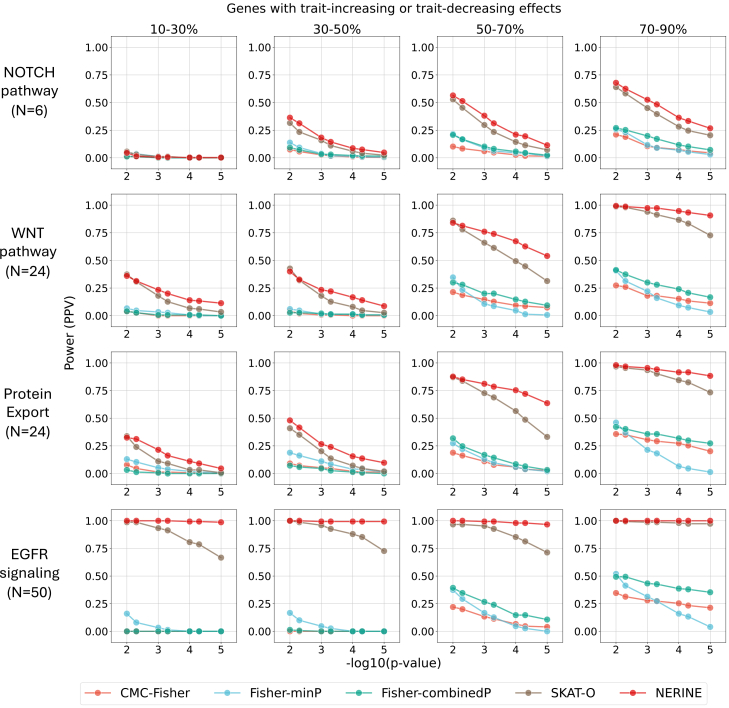
NERINE outperforms existing rare variant association tests in simulations Power was evaluated using a simulated binary trait (2,000 cases and 2,000 controls) across four canonical pathways (NOTCH pathway, WNT pathway, protein export, and EGFR signaling) with non-zero network effect (*θ* = 0.2). Noise was varied by the proportion of genes with non-zero effects (10%–90%), spanning highly noisy (only 10%–30% genes with effect) to highly informative (70%–90% genes with effect) networks. For each noise profile, 250 iterations were performed per network, and power was measured as the positive predictive value (PPV) at different significance cutoffs (1 × 10^−2^, 5 × 10^−3^, 1 × 10^−3^, 5 × 10^−4^, 1 × 10^−4^, 5 × 10^−5^, and 1 × 10^−5^) (STAR Methods). NERINE consistently outperformed existing rare variant association tests, with the largest gains in noisy settings. All tests were two-sided.

We benchmarked NERINE’s performance against existing gene- and pathway-based rare variant tests (CMC-Fisher, Fisher minimum *p* value, Fisher combined test, SKAT-O, and RVTT) in simulations under the alternative hypothesis (*θ* > 0) (Figures 2, S4, and S5, STAR Methods). NERINE consistently showed higher empirical power compared to other tests, especially in noisy networks (Figures 2, S4, and S5). Positive control experiments on LDL and HDL cholesterol (LDL-C and HDL-C) phenotypes in UKBB whole-exome sequencing (WES) data confirmed NERINE’s ability to detect significant burden in key lipid-related pathways in European ancestry-specific (Figures S6–S8; STAR Methods) as well as pan-ancestry (Figure S9; Table S2; STAR Methods) analyses. Compared with SKAT-O, NERINE yielded lower *p* values, demonstrating its ability to leverage network connectivity to improve power (Figure S6C). NERINE’s estimated gene-level effect sizes and directions recapitulated known biology^32^: rare loss-of-function (LoF) variants in *PCSK9* and *APOB* were associated with low LDL-C levels, and those in *LDLR* were linked to high LDL-C levels (Figure S6D). Similarly, for the HDL-C phenotype, it linked rare LoF variants in *ABCA1*, *LCAT*, and *APOA1* to low HDL-C levels and those in *CETP*, *LIPC*, *LIPG*, and *SCARB1* to high HDL-C levels. (Figure S6E).

### Selecting the most informative network topology with NERINE

NERINE leverages the edge geometry of gene networks to improve inference of rare variant burden. In simulations with canonical pathways (NOTCH, WNT, protein export, and EGFR signaling) for an artificial binary phenotype, NERINE yielded greater power with ground-truth topologies than with randomized ones (Figure S10; STAR Methods). Additionally, it achieved higher LLRs and lower *p* values with real topologies than with random ones in simulations focusing on significant lipid-related pathways for binarized LDL-C and HDL-C phenotypes in the UKBB (Figure S11; STAR Methods).

**Figure 3:**
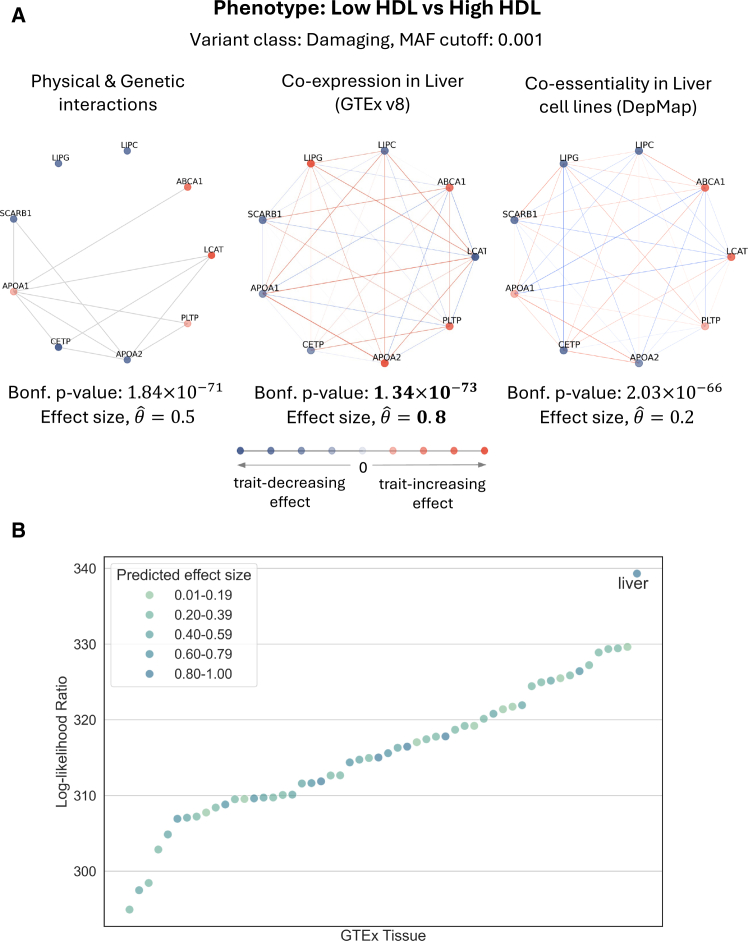
NERINE selects the most informative network topology (A) NERINE evaluates competing network topologies and selects the most informative one for a given gene set and phenotype. For core HDL-related genes, co-expression in liver (Bonf. *p* = 1.34 × 10^−73^; effect size, $\hat{\theta }=0.8$) outperforms PPI and liver co-essentiality networks when testing for rare damaging variant burden in low- vs. high-HDL-C individuals (*N*_*case*_ = 26,800; *N*_*control*_ = 27,178) in the UKBB (STAR Methods). Here, node colors denote the direction of gene effects (orange: trait increasing; purple: trait decreasing), with intensity reflecting magnitude. Co-expression and co-essentiality network edges indicate correlation (red: positive; blue: negative), with edge thickness reflecting the strength of the correlation. PPI network edges represent binary relationships and are colored gray. (B) Across 52 GTEx (v.8) tissues, NERINE identifies liver as the most informative context for core HDL-related genes when testing for rare damaging variant association with the binarized HDL-C phenotype in the UKBB, as in (A) (STAR Methods). NERINE yields $\hat{\theta }=0.8$, the highest LLR (339.32), and strongest significance (Bonf. *p* = 1.34 × 10^−73^) using the liver co-expression network.

NERINE does not uniformly select a single network source for all phenotypes and gene sets, as showcased using lipid-related gene networks for LDL-C and HDL-C in the UKBB (Table S3; STAR Methods). For example, liver co-expression best captured core HDL-related genes (Bonferroni [Bonf.] *p* = 1.34 × 10^−73^), physical/genetic interactions best described the VLDLR pathway (Bonf. *p* = 2.50 × 10^−21^), and liver co-essentiality best represented lipid metabolism (Bonf. *p* = 1.58 × 10^−46^) (Figures 3A and S12; Table S3).

For the binarized HDL-C phenotype in the UKBB, NERINE identified liver as the most relevant biological context for core HDL genes. Across 52 tissue-specific co-expression networks, liver had the strongest statistical support (*θ* = 0.8, Bonf. *p* = 1.34 × 10^−73^) (Figure 3B; Table S4). In this example, NERINE leveraged the variability observed in edge relationships to select the optimal tissue context with biological interpretability.

### Identifying rare variant associations in common diseases across canonical pathway networks

**Figure 4:**
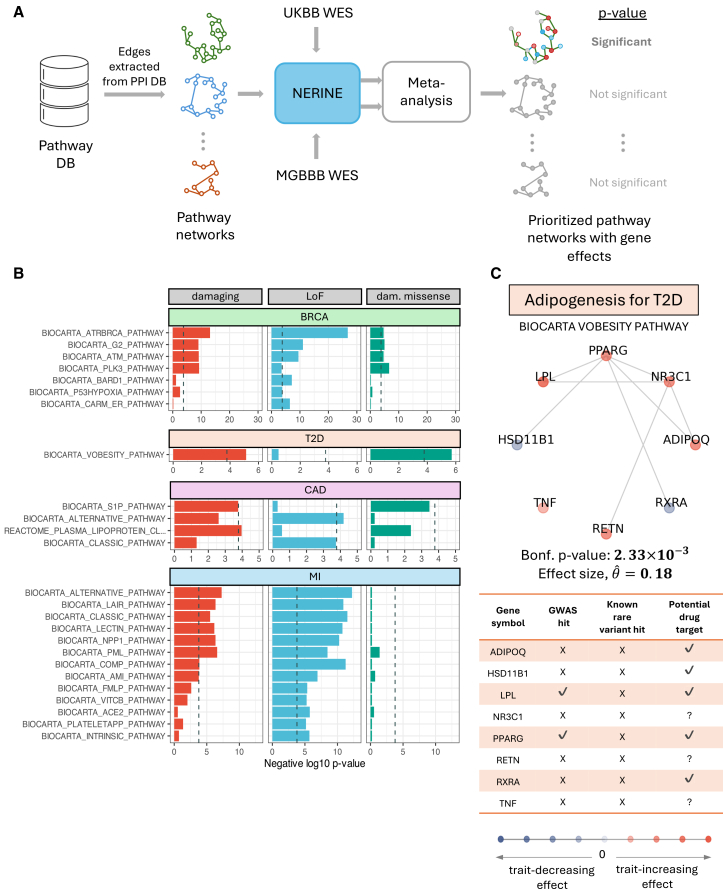
NERINE identifies rare variant burden in canonical pathway networks across common diseases in UKBB and MGBBB (A) NERINE’s application to case-control WES cohorts in UKBB and MGBBB for rare variant association across database pathway networks. NERINE tests 306 pathway networks (derived from curated physical/genetic interactions) and six variant classes (LoF, damaging missense, damaging, missense, synonymous, and neutral missense) (STAR Methods). Case-control cohort sizes (*N*_*case*_/*N*_*control*_): BRCA (UKBB: 10,648/91,886; MGBBB: 1,113/2,459), T2D (UKBB: 22,502/68,370; MGBBB: 747/2,188), CAD (UKBB: 4,561/12,321; MGBBB: 902/1,488), and MI (UKBB: 2,521/5,012; MGBBB: 326/2,068). Two-sided *p* values were meta-analyzed via Fisher’s combined test and Bonferroni corrected with Nyholt’s adjustment (STAR Methods). (B) Bonferroni-significant disease pathways with rare variant burden in LoF, damaging missense, and damaging categories are shown. Negative log-transformed Fisher’s combined *p* values are reported; the dashed gray line indicates the Bonferroni threshold of 0.05. (C) Adipogenesis pathway (BIOCARTA VOBESITY PATHWAY) shows significant rare damaging variant burden in T2D (avg. $\hat{\theta }$ = 0.18, Bonf. *p* = 2.33 × 10^−3^) and serves as a representative example of NERINE’s output. Top: NERINE-predicted gene effects (averaged across cohorts) across the network nodes (orange: trait increasing; blue: trait decreasing; intensity reflects magnitude). Bottom: information on gene-phenotype association in germline genetics from the GWAS catalog (https://www.ebi.ac.uk/gwas/), Genebass,^33^ SAIGE-GENE+,^19^ and disease-specific literature.^34^^,^^35^^,^^36^ Network genes, *RXRA*, *PPARG*, *ADIPOQ*, *TNF*, *NR3C1*, *LPL*, *RETN*, and *HSD11B1* were not identified in prior rare variant studies; only *PPARG* and *LPL* were identified in GWASs (bottom). Five of eight genes are under investigation as T2D drug targets,^37^^,^^38^^,^^39^^,^^40^^,^^41^^,^^42^^,^^43^ highlighting NERINE’s ability to identify therapeutically relevant pathways.

We applied NERINE to four diseases (BRCA, T2D, CAD, and early-onset MI), which impact 3%–13% of the population and have sufficient sample sizes in the UKBB and MGBBB (Figure 4A; STAR Methods). Prior rare variant association studies identified only a few associations^33^ (BRCA: 6, T2D: 3, CAD: 1, and MI: 1). We tested 306 canonical pathways with PPI-defined edges and six variant categories (STAR Methods). Significant associations were restricted to LoF and damaging missense variants, with no enrichment for benign missense or synonymous variants, as expected (Figure 4B; Table S5). Results were biologically coherent: BRCA was linked to DNA repair, cell cycle regulation, apoptosis, and hormone signaling pathways and cardiovascular diseases were linked to lipid metabolism, immune response, blood coagulation, and apoptosis (Figures S13–S15; Tables S6 and S7). NERINE-estimated gene effects in significant pathways per disease are provided in Tables S8, S9, S10, and S11.

As a representative example, we show the adipogenesis network (BIOCARTA VOBESITY PATHWAY) in T2D, where we detected a significant burden (average [avg.] $\hat{\theta }$ = 0.18, Bonf. *p* = 2.33 × 10^−3^) of rare damaging variants (i.e., LoFs and damaging missenses) (Figure 4C; Tables S5 and S9). We estimated non-zero effects in *RXRA*, *PPARG*, *ADIPOQ*, *TNF*, *NR3C1*, *LPL*, *RETN*, and *HSD11B1*—genes unrecognized in previous rare variant studies^19^^,^^33^ (Figure 4C; Table S9). *PPARG* and *LPL* were genome-wide association study (GWAS) hits.^34^^,^^35^^,^^36^ Sensitivity analysis showed that *LPL* was important for the database-wide significant network-level signal, whereas *PPARG* was not (Figure S16). Five of the genes had been studied as drug targets before^37^^,^^38^^,^^39^^,^^40^^,^^41^^,^^42^^,^^43^ (Figure 4C). Notably, NERINE’s findings aligned with known biology: *adiponectin* (*ADIPOQ*) deficiency reportedly increased insulin resistance and T2D risk.^39^ Damaging variants in *RXRA* and *HSD11B1* showed trait-decreasing effects, supporting their inhibition as potential therapeutic strategies for T2D,^40^^,^^41^^,^^42^^,^^43^ analogous to *PCSK9* in cardiovascular disease, where protective LoF and damaging missense variants guided effective therapy development.^44^^,^^45^

### Examples of new network-level associations in breast cancer and cardiovascular diseases

**Figure 5:**
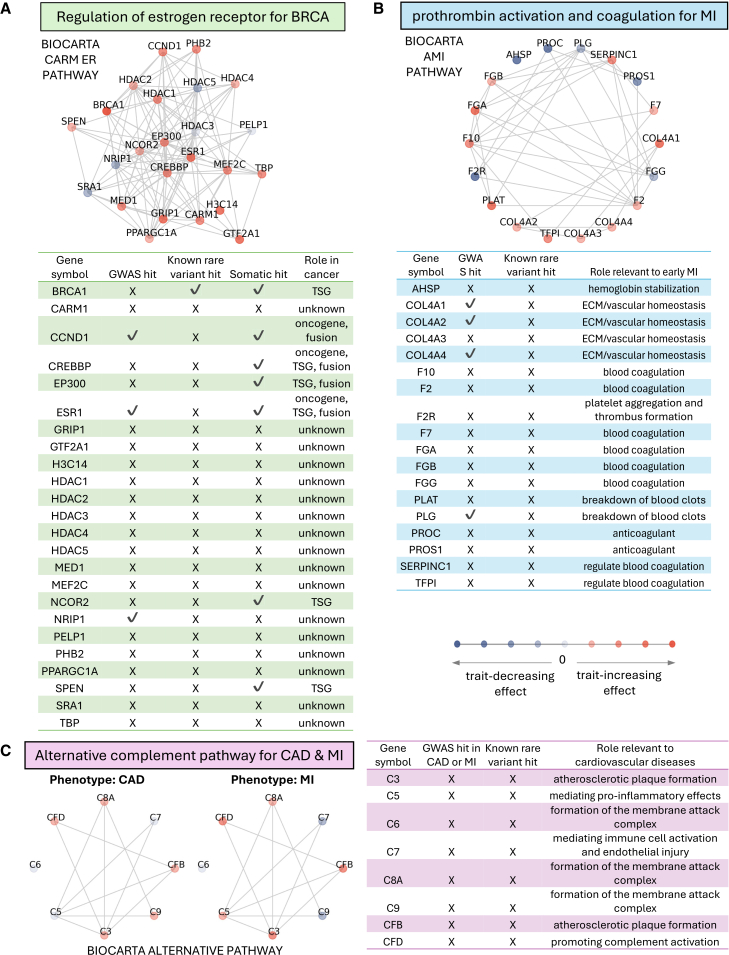
Examples of associations revealed by NERINE in breast cancer and cardiovascular diseases (A) Estrogen receptor regulation (BIOCARTA CARM ER PATHWAY) shows significant LoF burden in BRCA (avg. $\hat{\theta }$ = 0.33, Bonf. *p* = 1.04 × 10^−4^). Top: NERINE-predicted gene effects (averaged across biobanks). Bottom: information on gene-phenotype association from somatic and germline genetics with functional annotations. (B) Intrinsic prothrombin activation and coagulation network (BIOCARTA AMI PATHWAY) shows significant LoF burden in MI (avg. $\hat{\theta }$ = 0.18, Bonf. *p* = 3.50 × 10^−5^). Top: NERINE-predicted gene effects (averaged across biobanks). Bottom: information on gene-phenotype association from germline genetics with functional annotations. (C) Alternative complement system network (BIOCARTA ALTERNATIVE PATHWAY) shows significant LoF burden in CAD (left; avg. $\hat{\theta }$ = 0.03, Bonf. *p* = 1.94 × 10^−2^) and MI (middle; avg. $\hat{\theta }$ = 0.30, Bonf. *p* = 1.76 × 10^−10^). Right: information on gene-phenotype association from germline genetics with functional annotations. Across images, node color represents the direction of gene effect (orange: trait increasing; purple: trait decreasing; intensity reflects magnitude). Information sources: GWAS associations are from the GWAS catalog (https://www.ebi.ac.uk/gwas/); rare variant associations are from Genebass,^33^ SAIGE-GENE+,^19^ and disease-specific studies^46^^,^^53^^,^^54^; functional annotations for estrogen receptor regulation pathway members are from COSMIC (https://cancer.sanger.ac.uk/cosmic); and functional annotations of network genes are from SynGO (https://www.syngoportal.org/).

NERINE identified a significant burden in the estrogen receptor regulation pathway in BRCA (avg. $\hat{\theta }$ = 0.33, Bonf. *p* = 1.04 × 10^−4^; Figure 5A, top; Table S8). Among member genes, prior rare variant studies only implicated *BRCA1*.^46^^,^^47^ Sensitivity analysis showed that the network retained a nominally significant association after removing *BRCA1* variants (Figure S17). Beyond *BRCA1*, seven genes were previously linked to BRCA through GWAS or somatic cancer mutations (Figure 5A, bottom). Among the rest, *PHB2* (*prohibitin 2*), which showed a trait-increasing effect, recently emerged as a biomarker and therapeutic target.^48^^,^^49^^,^^50^ Furthermore, the predicted trait-decreasing effect of *HDAC5* LoF variants aligned with the observation that *HDAC5* inhibition induced intrinsic apoptosis in human BRCA cells and exerted an anti-neoplastic effect.^51^^,^^52^

Rare variant studies of cardiovascular diseases remained underpowered for uncovering mechanisms beyond lipid-related pathways despite sequencing over 100,000 subjects across multiple biobanks.^33^^,^^53^ NERINE identified network-level signals in several non-lipid pathways (Figures 4B, S14, and S15). Of note, the intrinsic prothrombin activation and coagulation cascade (BIOCARTA AMI PATHWAY), with a significant LoF burden (avg. $\hat{\theta }$ = 0.18, Bonf. *p* = 3.50 × 10^−5^), consists of genes involved in vascular basement membrane integrity (Figure 5B, top; Table S11). Among the members, several collagens (*COL4A1*, *COL4A2*, and *COL4A4*) and clotting factor *PLG* had previously been linked to MI by GWASs (Figure 5B, bottom) and murine studies^5^^,^^54^^,^^55^^,^^56^^,^^57^ rather than rare coding variants in humans.

Interestingly, we identified a significant LoF variant burden in the alternative complement pathway for both CAD (avg. $\hat{\theta }$ = 0.03, Bonf. *p* = 1.94 × 10^−2^) and MI (avg. $\hat{\theta }$ = 0.30, Bonf. *p* = 1.76 × 10^−10^), with no prior support from human genetics (Figure 5C). Functional studies in mice linked *C3* and *C5* to inflammation, endothelial dysfunction, and atherosclerosis.^58^^,^^59^^,^^60^ NERINE predicted trait-increasing effects for *C3* LoF variants in both CAD and MI and *C5* LoFs in MI. Additionally, rare LoF variants in *C8A*, *CFB*, and *CFD* showed trait-increasing effects on both conditions (Tables S10 and S11). These directionalities may help reconcile the growing experimental evidence that complement activation products in atherosclerotic plaques can exhibit both protective and pro-atherogenic properties.^60^ While the complement signal in cardiovascular diseases is intriguing, we observed differences between MGBBB and UKBB results, which may be due to MGBBB participants having a higher frequency of cardiac events, including thrombosis and inflammation, compared to those in the UKBB.

### Bridging genetics and experimental biology with NERINE in Parkinson's disease

PD, with expanding genomic and experimental datasets,^2^^,^^16^^,^^28^^,^^61^^,^^62^^,^^63^ is well suited for testing with NERINE. While GWASs^64^^,^^65^ and linkage analyses^66^^,^^67^^,^^68^ identified many PD-associated common variants and Mendelian rare variants in key genes, including *SNCA* (encoding *αS*), *GBA1*, and *LRRK2*, rare variant studies remained underpowered,^26^^,^^27^ especially in idiopathic PD (∼80%–85% PD cases^69^). To investigate the rare coding variant signal around known PD-associated loci, we first applied NERINE to Gene Ontology (GO) biological process modules significantly enriched for GWAS genes^64^^,^^65^^,^^70^ (STAR Methods; Figure S18A; Table S12; Methods S1). NERINE identified a significant rare LoF variant burden in the peptidyl-threonine modification module in two independent PD case-control cohorts from the UKBB and AMP-PD (avg. $\hat{\theta }$ = 0.9, Bonf. *p* = 4.34 × 10^−2^; Figure S18B; Table S13). Notably, NERINE predicted trait-increasing LoF burdens in *MCCC1* and *DYRK1A*, and trait-decreasing LoF effects in *FYN*, *USP8*, and *LRRK2* (Table S14; Methods S1).

**Figure 6:**
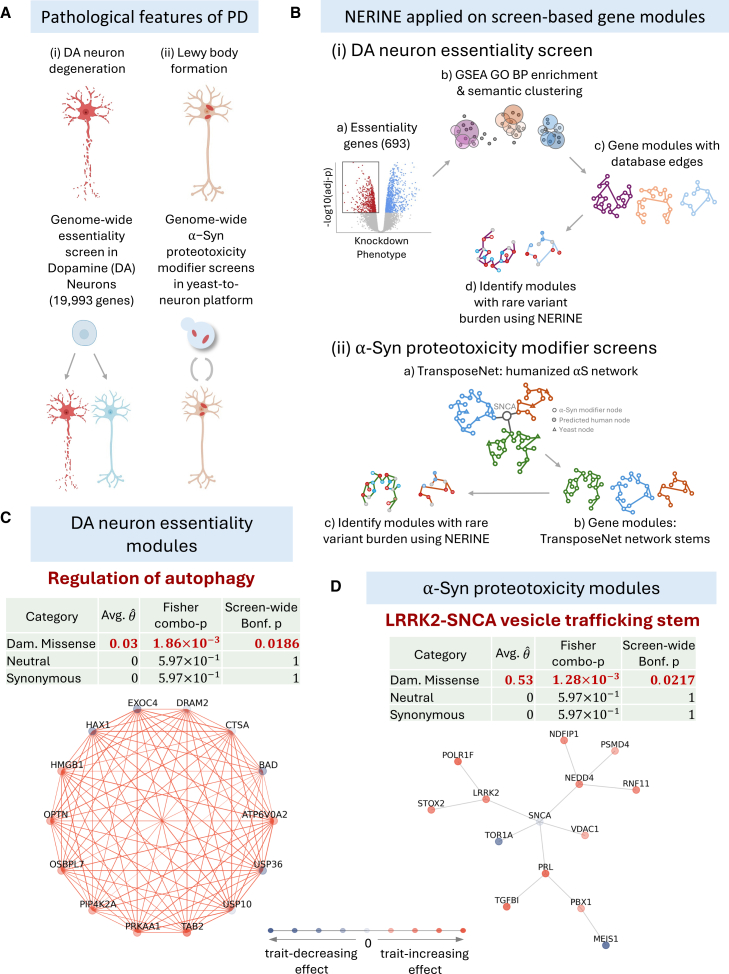
NERINE identifies rare variant burden in bespoke PD networks from model-system screens (A) Key pathological features of PD—Lewy body formation and dopaminergic (DA) neuron loss—targeted via (i) DA neuron essentiality and (ii) *α-synuclein* (*αS*) proteotoxicity screens. (B) Network hypotheses generation from model-system screens (STAR Methods). Network hypotheses were derived from (i) DA neuron essentiality genes (10 GO biological process [BP] modules) and (ii) *αS*-toxicity modifier genes (17 TransposeNet stems). Topologies for DA essentiality modules were generated using PPI, substantia nigra co-expression (GTEx v.8), and CNS co-essentiality (DepMap v.2023Q2) data. For *αS* proteotoxicity network stems, TransposeNet topologies^2^ were used. (C) NERINE identified screen-wide Bonferroni-significant burden of rare damaging missense variants across AMP-PD (2,117 cases and 1,095 controls) and UKBB-extreme (2,237 cases and 2,553 controls) cohorts in the *HMGB1-OPTN-*containing autophagy regulation module (avg. $\hat{\theta }$ = 0.03, Bonf. *p* = 1.86 × 10^−3^), with substantia nigra co-expression as the optimal topology. (D) The *LRRK2-SNCA-*containing vesicle trafficking and protein homeostasis stem showed screen-wide Bonferroni-significant burden of rare damaging missense variants (avg. $\hat{\theta }$ = 0.53, Bonf. *p* = 1.28 × 10^−3^) across AMP-PD and UKBB-sporadic cohorts (AMP-PD: 2,117/1,095; UKBB: 2,237/167,188). In (C) and (D), node color represents the direction of gene effect (orange: trait increasing; purple: trait decreasing; intensity reflects magnitude). In (C), edge color indicates the sign of the correlation (red: positive; blue: negative), while edge width reflects the correlation strength. In (D), TransposeNet edges represent binary relationships and are colored in gray.

As a complementary approach, we turned to biologically informed networks constructed from large-scale cellular and model-organism studies focused on PD pathology. Specifically, we applied NERINE on gene networks from screens targeting two pathological hallmarks of PD: *αS* aggregation in Lewy bodies and DA neuron loss in the mid-brain substantia nigra^71^^,^^72^ (Figure 6A; STAR Methods). We analyzed independent PD case-control datasets from the UKBB and AMP-PD.

#### Rare variant burden in DA neuron essentiality genes involved in autophagy regulation

An in-house genome-scale CRISPR screen of 19,993 genes identified 693 essential genes for DA neuron survival with significant enrichment in ten GO biological process modules, including autophagy regulation, mRNA processing, apoptosis, and cellular response to DNA damage (STAR Methods; Table S15). We generated networks from these ontological modules by imposing edge relationships from PPI, co-expression, and co-essentiality databases (Figure 6B, top; STAR Methods). NERINE was applied to two cohorts: AMP-PD sporadic and UKBB extreme (at recruitment, the median age of controls was ≥ that of cases). NERINE identified a significant damaging variant burden in the *HMGB1-OPTN-*containing autophagy regulation module (avg. $\hat{\theta }$ = 0.03, Bonf. *p* = 1.86 × 10^−2^; Figures 6C and S19; Tables S16 and S17). Intriguingly, *HMGB1* and *OPTN* showed trait-increasing effects for predicted damaging missense and damaging variants (Figures 6C and S19; Table S17). *HMGB1* impairment may inhibit autophagy and promote *αS* accumulation,^73^ while the disruption of *OPTN* function may cause improper clearance of damaged mitochondria (mitophagy), leading to neurodegeneration.^74^ Damaging variants in *USP10* also showed a trait-increasing effect (Figure S19; Table S17), consistent with the observation that *USP10* inactivation disrupted *αS*-containing aggresome formation, increasing toxicity.^75^

#### Rare variant burden in an *LRRK2-SNCA*-containing *αS* proteotoxicity module

NERINE was also applied to an *αS* proteotoxicity network assembled through “yeast-to-neuron” discovery screens^2^ targeting the “*αS* accumulation in Lewy bodies” feature of PD (Figure 6B, bottom). This network was generated by TransposeNet^2^ and validated in human induced pluripotent stem cell (iPSC) cortical and DA neurons.^2^^,^^16^ It converged with a proteome-scale proximity labeling screen for *αS* in neurons.^63^ The network stems spanned relevant pathways, including vesicle trafficking, mRNA metabolism and translation, mitophagy, oxidative metabolism, calcium/NFAT signaling, Toll-like receptor signaling, and purine metabolism, providing 17 network hypotheses to test with NERINE (Figure S20; Table S18).

NERINE identified a screen-wide significant burden of rare damaging missense variants in the *LRRK2-SNCA*-containing vesicle trafficking and protein homeostasis stem across AMP-PD and UKBB-sporadic cohorts (avg. $\hat{\theta }$ = 0.53, Bonf. *p* = 2.17 × 10^−2^; Figures 6D and S21; Tables S18 and S19). No member gene, except *LRRK2*, showed a significant association in prior studies.^26^^,^^33^ NERINE detected trait-increasing effects in several *SNCA* interactors, including *VDAC1*, *NEDD4*, and *TOR1A*, which were previously linked to PD pathology in experimental models.^61^^,^^62^^,^^76^^,^^77^^,^^78^^,^^79^^,^^80^ Interestingly, *PRL*, which showed the strongest trait-increasing effect, was not previously causally linked to PD. Beyond direct interactors of *SNCA* in the stem, *NDFIP1*, *PBX1*, and *RNF11* showed non-zero effects on PD risk, which had previously been linked to PD only in functional studies.^81^^,^^82^^,^^83^^,^^84^

### Convergence of an unbiased functional genomics screen in CRISPRi-induced synucleinopathy model with NERINE on *PRL-SNCA* interaction

**Figure 7:**
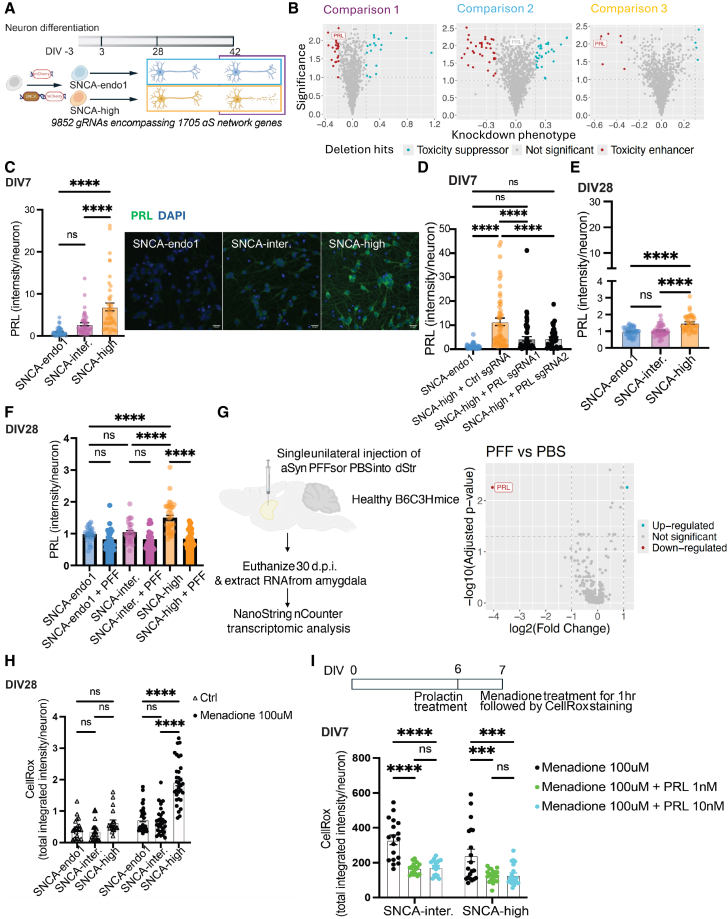
Unbiased functional genomics screens converge on an intraneuronal *SNCA-PRL* stress response (A) Top: timeline of CiS-CN differentiation. Bottom: iPSCs were transduced to overexpress either mCherry (control) or SNCA-mCherry, generating the *SNCA-*endo1 and *SNCA*-high lines, respectively. At DIV0, neurons were transduced with the sgRNA library. Neuronal samples were harvested and sequenced at DIV3, DIV28, and DIV42 using next-generation sequencing. Comparison 1 shows the comparison of the sgRNA frequencies between DIV42 *SNCA*-high neurons and DIV42 *SNCA-*endo1 neurons. Comparison 2 compares the sgRNA frequencies in *SNCA-*endo1 neurons between DIV42 and DIV28. Comparison 3 compares the sgRNA frequencies in *SNCA*-high neurons between DIV42 and DIV28. (B) Left: *PRL* sgRNA-containing neurons dropped out in comparison 1, indicating PRL knockdown was toxic to *SNCA*-high neurons compared to *SNCA-*endo1 neurons. Middle: no significant dropout in *PRL* sgRNA-containing neurons was observed in comparison 2, indicating PRL knockdown was non-toxic to DIV42 *SNCA*-endo1 neurons compared to DIV28 *SNCA-*endo1 neurons. Right: *PRL* sgRNA-containing neurons dropped out in comparison 3, indicating *PRL* knockdown was toxic to DIV42 *SNCA*-high neurons compared to DIV28 *SNCA*-high neurons. (C) Left: immunostaining data show PRL was upregulated in DIV7 *SNCA*-high neurons (*n* = 3, one-way ANOVA). Right: representative images acquired with a Nikon Eclipse Ti microscope, with solar power set to 10% and an exposure time of 50 ms for *PRL*. Scale bar: 20 μm. (D) *PRL* knockdown was validated with two different *PRL* sgRNAs from the screen library (*n* = 3, one-way ANOVA). DIV0 *SNCA*-high neurons were transduced with either control sgRNA or PRL sgRNA at MOI = 5. At DIV7, neurons were stained with *PRL* antibody and Hoechst. Images were captured with a Nikon Eclipse Ti microscope. *PRL* intensity per neuron is reported here. (E) Immunostaining data show *PRL* upregulation was diminished in DIV28 *SNCA-*high neurons (*n* = 3, one-way ANOVA). Images were captured with a Nikon Eclipse Ti microscope, with solar power set to 30% and a 30 ms exposure time for *PRL*. (F) Immunostaining data show *PRL* upregulation was diminished in DIV28 *SNCA*-high neurons treated with PFF (*n* = 3, one-way ANOVA). Images were captured with a Nikon Eclipse Ti microscope, with solar power set to 30% and a 30 ms exposure time for *PRL*. (G) Left: illustration of the PFF-induced mouse model. Amygdala tissue, micro-dissected from mice and injected with *αS* PFFs, was subjected to transcriptomic analysis using the NanoString Neuropathology panel (STAR Methods). Right: in comparison to PBS-injected control animals, which had no pS129-positive inclusions across all brain regions, PFF-injected mice showed a 16.6-fold downregulation of *Prl* mRNA expression in the amygdala region ipsilateral to the site of injection (*n* = 5 per group; 3 males/2 females). (H) CellRox assay shows that, with menadione treatment, oxidative stress in DIV28 *SNCA*-high neurons was significantly higher than in others of the same age (*n* = 3, two-way ANOVA). (I) Top: timeline of exogenous *PRL* assay. Bottom: CellRox assay shows that exogenous *PRL* significantly decreased menadione-triggered oxidative stress in DIV7 *SNCA*-OE neurons (*n* = 3, two-way ANOVA).

NERINE identified that genetic signals within the humanized TransposeNet *αS* proteinopathy network, which was originally derived from yeast-based screens^2^ and therefore lacked human cellular context. Since PD involves diverse cell types and peripheral systems, there are multiple avenues for mechanistic follow-up. To focus on neuronal mechanisms, we sought convergence with an independent genome-scale functional screen in a CiS-CN model^28^ designed to study genetic determinants of *αS* toxicity (STAR Methods). The CiS-CN model, comprising two *SNCA*-overexpression (*SNCA*-OE) clones (*SNCA-*high and *SNCA-*intermediate) and a control clone (*SNCA-*endo1), provided a tractable yet physiologically relevant system for studying *αS* aggregation and toxicity in cortical neurons^28^^,^^85^ (STAR Methods). Physical and genetic interactors of *αS* were targeted in the CiS-CN model, including all genes from the TransposeNet *αS* proteinopathy network, using a custom sgRNA library (STAR Methods). The full screen will be described in a forthcoming publication. We assessed sgRNA representation at start, day *in vitro* (DIV)28, and DIV42 and looked for the dropout of sgRNAs in three comparisons: (1) *SNCA*-high vs. *SNCA*-endo1 at DIV42, (2) DIV42 vs. DIV28 in *SNCA*-endo1, and (3) DIV42 vs. DIV28 in *SNCA*-high (Figures 7A and 7B). Genes showing significant sgRNA dropout in MAGeCK-iNC analysis (false discovery rate [FDR] < 0.1) were classified as *αS* toxicity enhancers (STAR Methods).

In these comparisons, *PRL* emerged as a particularly intriguing finding. NERINE had already pinpointed *PRL* within the *LRRK2-SNCA*-containing stem (Figure 6D), where damaging missense variants showed the strongest trait-increasing effect. In the CiS-CN CRISPRi screen, sgRNAs targeting *PRL* significantly dropped out in *SNCA*-high vs. *SNCA*-endo1 at DIV42 (comparison 1) and from DIV28 to DIV42 in *SNCA*-high clones (comparison 3). Thus, based on these convergent results, *PRL* was selected as a candidate for further experimental validation.

### An intraneuronal *SNCA-PRL* stress response linked to *αS* toxicity

While *Prl* is predominantly expressed in neuroendocrine tissue, its unexpected identification in our neuronal CRISPRi screen suggests a possible cell-autonomous role in neurodegeneration. Previous studies have reported *PRL* expression in rodent neurons under stress,^86^ possibly detectable only under such conditions. There is also evidence of post-translational regulation of *Prl* levels despite low mRNA expression.^87^ Overall, prevailing evidence attributes *Prl* effects mainly to its pituitary-derived secretion.

To investigate whether *Prl* is part of an intraneuronal *αS*-related stress response mechanism, we performed immunostaining in neurons of different ages (STAR Methods). *Prl* expression was significantly increased in *SNCA-*OE neurons, with ∼7-fold-higher expression in *SNCA-*high and 2.5-fold-higher expression in *SNCA-*intermediate than in *SNCA-*endo1 neurons at DIV7 (Figure 7C). This elevation was abrogated, as expected, by our sgRNAs directed to *PRL* (Figure 7D). Importantly, *Prl* levels were reduced over time, such that by DIV28, *Prl* levels in the *SNCA-*high lines were only ∼1.5-fold higher than in *SNCA-*endo1 neurons (Figure 7E). These data collectively suggest a potentially protective role of *αS*-induced *Prl* that diminishes over time.

We next tested whether inducing *αS* aggregation, thereby reducing soluble *αS* levels,^85^ in cellular and animal models via pre-formed *αS* fibrils (PFFs)^29^^,^^88^ led to reduced *Prl* levels. Our CiS-CN neurons, challenged with 7 days of exposure to PFFs, showed a reduction in *Prl* levels as indicated by immunofluorescence (Figure 7F). We further considered whether this was transcriptional or post-transcriptional. *PRL* mRNA expression levels were low in our models, possibly related to the immaturity of the iPSC-derived neurons. We thus turned to a well-established mouse PFF model.^29^ We unilaterally injected PBS vs. 5 μg of PFFs (2.5 μL volume) into the dorsal striatum of mice (Figure 7G, left; STAR Methods). Mice were aged for 30 days post-injection. By this stage, as previously described,^89^ the *αS* aggregation pathology “spreads” distally, reaching the cortex and the amygdala, two brain regions highly susceptible to *αS* pathology in later-stage PD. We harvested amygdala mRNAs and assessed gene expression using the Nanostring Neuropathology panel. In comparison to PBS-injected control animals, which showed no pS129-positive inclusions across all brain regions (data not shown), PFF-injected mice showed a 16.6-fold downregulation of *Prl* mRNA expression in the amygdala region ipsilateral to the site of injection (*n* = 5 per group; 3 males/2 females; Figure 7G, right), consistent and indeed stronger than our findings in the shorter-term human CiS-CN model. At least the intact rodent model data suggest that this effect is partly transcriptional.

Our data indicate that, in the context of aging and fibrillar *αS* pathologies, *Prl* levels drop intraneuronally and may sensitize neurons to *αS*-induced cytotoxicity. To determine whether *Prl* directly protects against *αS* toxicity, we assessed its neuroprotective effects against oxidative stress in our CiS-CN models. By DIV28, *SNCA-*high neurons exhibited heightened sensitivity to menadione-induced oxidative stress,^90^ measured with the fluorogenic probe CellRox (Figure 7H). In *SNCA-*high and *SNCA-*endo1 models, we pre-conditioned DIV6 CiS neurons with *Prl* for 24 h before treatment with menadione (Figure 7I, top). Importantly, exogenous *Prl* treatment significantly decreased oxidative stress in CiS neurons (Figure 7I, bottom), strongly suggesting a neuroprotective effect against *αS*-induced toxicity. These data imply that aged neurons in which *αS* aggregates are associated with reduced *Prl* levels and increased vulnerability to oxidative stress. Taken together, the convergence of our forward genetics iPSC screen with NERINE uncovered an unexpected *αS*-*Prl* intraneuronal stress response, suggesting that *Prl* may play a crucial role in neuronal resilience to *αS* pathology in PD.

## Discussion

NERINE bridges human genetics and experimental biology by embedding rare variant burden analysis within biological networks. Its ability to select among competing network topologies distinguishes it from existing methods. By leveraging edge geometry, NERINE improves statistical power, as demonstrated in our analyses, even when network structures are poorly defined.

These capabilities enable NERINE to identify significant rare variant burden in biologically plausible pathways across diseases, uncovering associations missed by single-gene tests. In cardiovascular diseases, we identify networks involving collagens and inflammatory response genes. The association of collagens with MI is consistent with their function in maintaining atherosclerotic plaque stability^91^ and in coagulation via platelet effects and interactions with clotting factors.^92^ Notably, NERINE implicates inflammatory response gene modules in CAD and MI, supporting anti-inflammatory therapies as potential alternatives to LDL-lowering drugs.^93^

Our results highlight two major contributions. First, NERINE provides human genetics support for findings from model system screens. For example, genes within the *LRRK2-SNCA*-containing vesicle trafficking and protein homeostasis stem (e.g., *NEDD4*, *NDFIP1*, *VDAC1*, *PBX1*, *TOR1A*, and *RNF11*), previously implicated in PD only experimentally,^61^^,^^62^^,^^76^^,^^77^^,^^78^^,^^79^^,^^80^^,^^81^^,^^82^^,^^83^^,^^84^^,^^94^^,^^95^^,^^96^ are now corroborated with rare variant evidence. Second, NERINE helps resolve conflicting hypotheses. For instance, within the PD GWAS peptidyl-threonine modification module, *DYRK1A* has previously been functionally implicated in PD in opposing ways: its haploinsufficiency reduces kinase activity and exacerbates DA neuron degeneration in mice,^97^ whereas other studies suggest an opposing role through phosphorylation of *αS*.^98^ NERINE’s findings support the latter direction, offering some resolution of the matter.

NERINE’s application to experimentally derived networks complements traditional pathway-based investigations in PD.^99^^,^^100^ Prior analyses based on pathway-level polygenic risk scores and rare variant tests (e.g., SKAT-O) have pointed to broad mechanisms involving hundreds of genes, including endolysosomal trafficking, GPCR signaling, neuronal transmission, and immune response,^99^^,^^100^ at the expense of resolution. By incorporating experimentally derived network topologies, NERINE adds biological specificity, facilitating the generation of mechanistic hypotheses for follow-up studies.

Among NERINE’s PD findings, the most intriguing one is *PRL*, supported by both NERINE and our neuronal CRISPRi screen. *PRL* encodes the pituitary hormone *Prl*, which TransposeNet unexpectedly introduced into the *αS* proteotoxicity network alongside *LRRK2* and *SNCA*.^2^ Mechanistically, a connection between *Prl* and *αS* has been unclear, as the neuronal expression of *Prl* remains speculative.^86^^,^^101^ The neuroprotective effect of *Prl* against oxidative stress has exclusively been attributed to exogenous pituitary secretion.^86^^,^^102^^,^^103^^,^^104^ It is thus remarkable that *Prl* LoF is implicated by both human genetics and functional screening of *αS* proteotoxicity modifiers: NERINE associates rare, predicted-deleterious missense mutations with PD risk and *Prl* knockdown enhances *αS* toxicity in neurons.

Our data suggest an early, most likely post-translational, intraneuronal response in which *Prl* is upregulated with *αS* OE but declines over time and with *αS* aggregation—partly via transcriptional changes (shown in *αS* PFF mouse models). This decrease leads to increased sensitization to *PRL* knockdown and to exogenous stress, as demonstrated by the oxidative stressor menadione. Although pituitary function in *PRL* variant-carriers in our study cohorts could not be directly assessed, the literature provides additional context. Recent proteomic studies have shown altered *Prl* levels in cerebrospinal fluid (CSF) and plasma in patients with PD^105^^,^^106^; notably, CSF *Prl* was the top feature in a PD vs. control classifier model for the Harvard Biomarkers Study cohort (a subset of the MGBBB),^105^ independent of levodopa effect. Together, these data support a previously unrecognized neuron-intrinsic role for *Prl* in PD under *αS* stress.

### Limitations of the study

NERINE is limited to dichotomous traits; continuous traits need to be binarized before testing. Covariate correction is not a part of the methodology. For continuous traits, covariates were residualized via regression as a preprocessing step. For binary traits, NERINE performs ancestry-stratified analysis with post hoc *p* value combination using Fisher’s method. For large networks (>50 genes), NERINE’s test statistic may deviate from its asymptotic distribution, limiting its reliable application to smaller networks. Finally, NERINE performs inference at the network level and does not provide gene-level *p* values. It provides only maximum likelihood estimates of gene-level effect sizes and directions. While protective effects of LoF variants are of special therapeutic interest (e.g., *PCSK9* inhibitors in hypercholesterolemia treatment^44^^,^^45^), their detection in binary traits is severely power limited. Moreover, the potential presence of gain-of-function variants can further complicate interpretation. Given the lack of gene-level significance evaluation, our inference of protective effects is not definitive.

In sum, NERINE provides a robust framework for attributing putative disease-causing factors to experimentally derived molecular networks. Such a method is valuable for complex diseases, such as PD, where traditional approaches are underpowered due to the limited availability of well-phenotyped genomic data. As large-scale genetic and molecular datasets become commonplace,^107^^,^^108^ we anticipate that research programs in which human genetics and experimental biology mutually reinforce each other, such as those enabled by NERINE, will play an increasingly important role in identifying disease-relevant signals, resolving conflicting evidence, and uncovering druggable targets.

## Resource availability

### Lead contact

Requests for further information and resources should be directed to and will be fulfilled by the lead contact, Shamil R. Sunyaev (ssunyaev@hms.harvard.edu).

### Materials availability

This study did not generate new materials.

### Data and code availability

Canonical pathway gene sets were obtained from MSigDB^109^ (v.7.3; https://www.gsea-msigdb.org/gsea/msigdb/human/collections.jsp). Human physical PPIs were downloaded from STRING^110^ (v.11.5; https://string-db.org/cgi/download), HuRI (http://www.interactome-atlas.org/download: last accessed in January 2022), and inBio Map^111^ (https://www.intomics.com/inbio/map: last accessed in January 2022) databases. Additional genetic interactions were obtained from the Megchelenbrink et al. study.^112^ TransposeNet’s humanized *α-synuclein-*, *β-amyloid-*, and *TDP-43*-modifier networks were obtained from our prior study.^2^ Bulk expression data in TPM format from different human tissues were downloaded from the GTEx^113^ (v.8; https://www.gtexportal.org/), and gene dependency data in different cell lines were downloaded from DepMap^114^ (release: 2023Q2; https://depmap.org/portal/data_page/?tab=allData).

WES and phenotypic data from the UKBB,^115^ available through https://ams.ukbiobank.ac.uk, were accessed via application 41250 and processed on the DNAnexus platform (https://ukbiobank.dnanexus.com/landing). MGBBB^116^ WES and phenotypic data were accessed via https://biobankportal.partners.org/ (PI: V.K.) and were restricted to affiliated investigators. AMP-PD^117^ whole-genome sequencing (WGS) and phenotypic data (v.2.5; release 2022) were accessed through the AMP-PD Knowledge Platform (https://www.amp-pd.org).

NERINE’s source code is available on GitHub (https://github.com/snz20/NERINE) and Zenodo (https://doi.org/10.5281/zenodo.19209293). RVTT was run by adapting the code from https://github.com/snz20/RVTT (Zenodo, https://doi.org/10.5281/zenodo.10627549). CMC-Fisher, Fisher’s combined test, and SKAT-O were run on R (v.4.3.2) using *stats* (v.4.3.2), *poolr* (v.1.2.0), and *SKAT* (v.2.2.5) packages, respectively. *MAGeCK-iNC* analysis was performed using the *MAGeCK*^118^ (v.0.5.9.2) package on python (v.2.7), and GO gene set enrichment analysis was performed using *GSEApy* (v.1.1.3) on python (v.3.12.4). Comprehensive information on study-related resources is provided in the key resources table.

## Acknowledgments

We thank Drs. Matthew Stevens, Richard Sherwood, Benjamin Neale, and Isabel Lam for their valuable insights. S.N. is supported by the 10.13039/100000002NIH grant R35GM127131, the Sudarsky Scholar Award (10.13039/100005292Brigham and Women's Hospital, Movement Disorders Division), and the Australian Parkinson’s Mission. S.R.S. is supported by NIH grants U01HG012009, R35GM127131, and R01MH101244. V.K., X.W., L.S., and experiments in PD neuronal models are supported by Aligning Science Across Parkinson’s Initiative (ASAP) award ASAP-000472 (principal investigator [PI]: L.S., co-PI: V.K.). V.K. also acknowledges support from an APDA Center for Advanced Research grant, NIH grant R01NS109209, the 10.13039/100003194New York Stem Cell Foundation Robertson Investigator award (NYSCF-R-I49), Mrs. Nancy Black Simches, and the Ocko family Parkinson’s Disease Innovation Award. X.W. also acknowledges support from the NIH grant T32AG000222 (PI: Bruce A. Yankner). We analyzed WES data from the UKBB (application 41250, PI: C.S.C.; Mass General Brigham IRB 2020P002093; NIH R01HG010372, PI: S.R.S.), a global biomedical resource supported by the Wellcome Trust, UK Medical Research Council, the Department of Health, the Scottish government, the Northwest Regional Development Agency, British Heart Foundation, and Cancer Research UK. We also used WES and phenotypic data from ∼50,000 participants from the MGBBB, a biorepository of consented patient samples at Mass General Brigham (parent organization of Massachusetts General Hospital and Brigham and Women’s Hospital), and WGS data from AMP-PD, a public-private partnership managed by FNIH and funded by Celgene, GSK, the Michael J. Fox Foundation, NINDS, Pfizer, and Verily. AMP-PD investigators did not review this work. We thank all the participants, clinical investigators, and research teams who contributed to UKBB, MGBBB, and AMP-PD.

## Author contributions

S.N., V.K., and S.R.S. conceived the project and interpreted the results; S.N., X.W., V.K., and S.R.S. wrote the manuscript; S.N. and S.R.S. developed NERINE; S.N. analyzed all cohorts; A.R.M. assisted with QC and preprocessing; C.S.C. assisted with UKBB analyses; S.N., S.R.S., N.O.S., and R.M.G. interpreted lipid and cardiac phenotypes; X.W. and V.K. designed the CiS-CN model experiments; X.W., R.S., and E.E. generated CiS neurons and performed experiments; X.W. and S.N. analyzed experimental data and interpreted results with V.K.; D.R., A.B.H.H., J.A., and L.S. performed the CRISPR-Cas9 experiments in DA neurons and identified essential genes and GO modules; K.C.L. performed the mouse PFF model experiments, analyzed the data, and interpreted the results with V.K., X.W., and S.N.; and all authors read and helped edit the manuscript.

## Declaration of interests

V.K. is a cofounder of and senior advisor to DaCapo Brainscience and Yumanity Therapeutics, companies focused on CNS diseases.

## STAR★Methods

### Key resources table

**Table:** 

REAGENT or RESOURCE	SOURCE	IDENTIFIER
**Deposited data**		

UK Biobank WES and phenotypic data	UK Biobank; Backman et al.^115^	https://www.ukbiobank.ac.uk/
MassGeneral Brigham Biobank WES and phenotypic data	MassGeneral Brigham Biobank; Boutin et al.^116^	https://www.massgeneralbrigham.org/en/research-and-innovation/participate-in-research/biobank
AMP-PD WGS and phenotypic data	AMP-PD knowledge platform; Iwaki et al.^117^	https://amp-pd.org/
MSigDB v7.3 curated gene sets (C2) from canonical pathways (CP)	Liberzon et al.^109^	https://www.gsea-msigdb.org/gsea/msigdb/human/genesets.jsp?collection=CP
STRING v11.5 physical human protein-protein interaction data	Szklarczyk et al.^110^^,^^119^	https://string-db.org/cgi/download
HuRI protein interaction data (last accessed Jan 2022)	Luck et al.^6^	http://www.interactome-atlas.org/download
inBio Map (last accessed Jan 2022)	Li et al.^111^	https://www.intomics.com/inbio/map
Genetic interactions from Megchelenbrink et al., 2015	Megchelenbrink et al.^112^	https://www.pnas.org/doi/full/10.1073/pnas.1508573112#supplementary-materials
TransposeNet humanized α-synuclein-, β-amyloid-, and TDP-43-modifier networks	Khurana et al.^2^	https://www.cell.com/cell-systems/fulltext/S2405-4712(16)30445-8#supplementary-material
GTEx v8 bulk expression data in TPM format across human tissues	GTEx Consortium^113^	https://www.gtexportal.org/
DepMap (release 2023Q2) gene dependency data	DepMap portal; Tsherniak et al.^34^	https://depmap.org/portal/data_page/?tab=allData
dbNSFP v4.3a	Liu et al.^120^	https://sites.google.com/site/jpopgen/dbNSFP
AlphaMissense hg38 scores v1	Cheng et al.^121^	https://zenodo.org/records/8208688

**Software and algorithms**		

NERINE v1.0.0	This paper	https://github.com/snz20/NERINE/releases/tag/v1.0.0; https://doi.org/10.5281/zenodo.19209293
RVTT v1.1	Bendapudi et al.^15^; Hallacli et al.^16^	https://github.com/snz20/RVTT/releases/tag/RVTT_version1.1
Python v3.12.4	Python Software Foundation	https://www.python.org
Python v2.7	Python Software Foundation	https://www.python.org
R v4.3.2	The R Foundation for Statistical Computing	https://www.r-project.org
CMC-Fisher test from stats package from base R v4.3.2	Fisher^122^	https://www.rdocumentation.org/packages/stats/versions/3.6.2/topics/fisher.test
Fisher’s combined test from poolr package (v1.2.0) in R	Fisher^122^	https://cran.r-project.org/web/packages/poolr/index.html
SKAT-O test from SKAT package (v2.2.5) in R	Lee et al.^18^	https://cran.r-project.org/web/packages/SKAT/index.html
Nearest positive definite matrix calculation using *nearPD* function from the Matrix package v1.6-5	Higham^123^	https://www.rdocumentation.org/packages/Matrix/versions/1.6-5/topics/nearPD
VEP v109	Ensembl; McLaren et al.^124^	https://www.ensembl.org/vep
REVEL plugin from VEP v109 (GRCh38 scores from version May 2021)	Ioannidis et al.^125^	https://github.com/Ensembl/VEP_plugins/blob/release/109/REVEL.pm
MAGeCK-MLE from the MAGeCKFlute (v2.6.0) R (v4.3.2) package	Wang et al.^126^	https://www.bioconductor.org/packages/3.11/bioc/vignettes/MAGeCKFlute/inst/doc/MAGeCKFlute.html
Gene set enrichment analysis using the Enrichr API from GSEApy v1.1.3	Xie et al.^127^	https://gseapy.readthedocs.io/en/latest/introduction.html#gseapy-enrichr-module
REVIGO	Supek et al.^128^	http://revigo.irb.hr/
MAGeCK-iNC from MAGeCK python (v2.7) package v0.5.9.2	Li et al.^118^	https://kampmannlab.ucsf.edu/mageck-inc
ImageJ2 Macro Software	Schneider et al.^129^	https://imagej.net/
Live-Cell Imaging and Analysis System: Incucyte® S3	Sartorius	https://www.sartorius.com/en/products/live-cell-imaging-analysis/live-cell-analysis-instruments/s3-live-cell-analysis-instrument
One- and two-sided ANOVA from PRISM v10.6.1	PRISM	https://www.graphpad.com/features
NanoString differential expression analysis using ROSALIND’s nCounter module	ROSALIND	https://www.rosalind.bio/en/knowledge/methods#nanostring-ncounter-gene-expression

**Experimental models: Cell lines**		

H9 Human embryonic stem cells	MSKCC stem cell core facility	WA-09
GM29371∗C Human induced pluripotent stem cells	Coriell Institute for Medical Research	GM29371∗C

**Reagents**		

Essential 8 Medium	Gibco/Thermo Fisher Scientific	A1517001
Vitronectin	Thermo Fisher Scientific	A14700
Dulbecco’s PBS (DPBS)	Thermo Fisher Scientific; Invitrogen; Thermo Fisher Scientific	14040182; 14200075; 14190136
StemPro Accutase Cell Dissociation Reagent	Gibco/Thermo Fisher Scientific	A11105-01
Neurobasal medium	Gibco/Thermo Fisher Scientific	21103–049
N2 supplement	Life Technologies; Gibco/Thermo Fisher Scientific	17502048; 17502-048
B27 supplement	Life Technologies; Gibco/Thermo Fisher Scientific	17504044; 17504-044
L-glutamine	Gibco/Thermo Fisher Scientific	25030–081
Penicillin-Streptomycin	Gibco/Thermo Fisher Scientific	15140122
LDN193189	Reprocell	04–0074
SB431542	R&D Systems	1614
CHIR99021	R&D Systems	4432
SHH	R&D Systems	464-SH
Y-27632 ROCK inhibitor	Bio-Techne	HY-10583
DMEM/F12	Gibco/Thermo Fisher Scientific	11320033
Geltrex	Life Technologies	A1413201
Ascorbic Acid	Sigma-Aldrich	A4034
GDNF	Gibco/Thermo Fisher Scientific	450–10
TGFβ3	Gibco/Thermo Fisher Scientific	100-36E
BDNF	R&D Systems; PeproTech	248-BDB; 450-02
dbcAMP	Sigma-Aldrich	4043
Fibronectin	Gibco/Thermo Fisher Scientific	356008
Laminin	R&D Systems	3400-010-03
IWP-2	Tocris Bioscience	3533
FGF-18	PeproTech	100–28
Doxycycline hydrochloride	Sigma-Aldrich	D3447-500MG
DAPT	R&D Systems	2634
Human CRISPR Gattinara pooled knockout library	Addgene; DeWeirdt et al.^130^	Pooled Library #136986
Stemflex	Gibco/Thermo Fisher Scientific	A33493
Matrigel Matrix	Corning	356231
Knockout DMEM	Gibco/Thermo Fisher Scientific	10829–018
Knockout DMEM/F12	Gibco/Thermo Fisher Scientific	12660–012
MEM Non-Essential Amino Acids	Gibco/Thermo Fisher Scientific	11140–050
NT-3	PeproTech	450–03
Mouse Laminin	Thermo Fisher Scientific	23017–015
ROCK inhibitor	PeproTech	1293823
DMEM/F12	Gibco/Thermo Fisher Scientific	11320–033
Neurobasal-A	Gibco/Thermo Fisher Scientific	10888–022
GlutaMAX Supplement	Gibco/Thermo Fisher Scientific	35050–061
BioCoat Poly-D-Lysine coated plates	Corning	356470
PFA	EM Sciences	15710
Triton X-100	Sigma-Aldrich	T8787
BSA	Sigma-Aldrich	A7906
Poly-L-Ornithine solution	Sigma-Aldrich; Sigma-Aldrich	P3655; P4957
Human *Prolactin* Recombinant Protein	Thermo Fisher Scientific	100-07-10UG
Menadione	Thermo Fisher Scientific	ICN10225925
CELLROX™ Green Reagent	Thermo Fisher Scientific	C10444
Terrific Broth	Sigma-Aldrich	T9179
Ampicillin	Fisher Scientific	BP1760
NaCl	Fisher Scientific	S271
Tris	Fisher Scientific	BP152
EDTA	Sigma-Aldrich	E5134
Amicon Ultra-15 centrifugal filter unit	Merck Millipore	UFC901008
Superdex 200 column	Cytiva	17517501
HiTrapQ HP column	Cytiva	645932
TRI Reagent	Sigma-Aldrich	T9424
TissueRuptor II and probe	Qiagen	9002755; 990890
Direct-zol RNA MiniPrep kit	Zymo	R2050
4200 TapeStation	Agilent	G2991AA

**Antibodies**		

Anti-PRL	Thermo Fisher Scientific	MA1-10597
Hoechst 33342	Thermo Fisher Scientific	H3570

**Experimental models: Organisms/strains**		

Wildtype B6C3F1 mouse	The Jackson Laboratories	Stock 100010
BL21 (DE3) RIL-competent *E. coli* cells	Agilent Technologies	230245

### Experimental model and study participant details

#### Cohort selection for lipid phenotypes in UKBB

For positive control experiments, we utilized jointly genotyped variant calls from the whole exomes of 469,589 individuals in the UK biobank (UKBB). We focused on two lipid related phenotypes: direct LDL cholesterol (LDL-C; data field: 30780) and HDL cholesterol (HDL-C; data field: 30760) (Figures S6–S9). We created two dichotomous phenotypes: (i) high LDL vs. low LDL and (ii) low HDL vs. high HDL, by selecting individuals belonging to the top and bottom deciles of the distributions for LDL-C and HDL-C measurements. European ancestry groups had the largest sample sizes: LDL-C (30,007 cases and 28,673 controls), and HDL-C (26,800 cases and 27,178 controls).

#### Study cohorts for common disease phenotypes

Our study included the analyses of four representative, high-prevalence complex diseases of public health relevance: breast cancer (BRCA), type II diabetes mellitus (T2D), coronary artery disease (CAD), and early-onset myocardial infarction (MI). These phenotypes were chosen from the UK Biobank because large sample sizes were available, and replication cohorts existed in the MGB Biobank. They served to showcase NERINE’s applicability in comprehensive database searches for rare variant network burden. These analyses included whole-exome sequencing (WES) data from the UK Biobank (UKBB) and the Mass General Brigham Biobank (MGBBB).

Cohorts for BRCA, T2D, CAD, and MI were selected primarily based on the summary diagnoses recorded in the data field 41270 as ICD-10 codes. For BRCA, the case group consisted of unrelated females of European ancestry with ICD-10 code C50 and the control group consisted of unrelated European females of age 60 or above with no history of neoplasms (ICD-10 codes: C00-C97 and D00-D48). The resulting cohort had 10,648 cases and 91,886 controls. For T2D, we included unrelated European individuals with ICD-10 code E11 in the case group and unrelated European individuals with no endocrine, nutritional and metabolic diseases (ICD10 codes: E00-E90) in the control group. The resulting cohort had 22,502 cases and 68,370 controls. We created an age-stratified case-control cohort for the CAD phenotype where cases consisted of unrelated European individuals of age ≤65 with ICD-10 code I25 and controls consisted of unrelated European individuals of age >65 with no diseases of the circulatory system (ICD-10 codes: I00-I99). This left us with 4,561 cases and 12,321 controls. Finally, for the MI phenotype, our cohort consisted of 2,521 cases and 5,012 controls. Cases included unrelated European individuals with ICD-10 code I 21. Only males with age ≤55 and females with age ≤65 were included in the case group. Whereas controls consisted of unrelated European individuals of age ≥ 69 who have no history of any disease of the circulatory system (ICD-10 codes: I00-I99).

Parkinson’s disease (PD) was chosen as a case study to demonstrate NERINE’s ability to bridge human genetics with model-system screens. UKBB 500K WES data and whole-genome sequencing (WGS) data from the AMP-PD consortium were utilized for this analysis. Two genome-scale screens, targeting the main pathological features of the disease, were accessible to our lab, and provided experimental gene networks for testing. We focused on sporadic disease cases as they constitute ∼85% of the PD population.

For sporadic PD phenotype, we created a discovery cohort from UKBB individuals by including unrelated European individuals with ICD-10 code G20 and no family history of PD as cases. The control group consisted of unrelated European individuals with no history of PD and other nervous system diseases (ICD-10 codes: G00-G99). In the original UKBB cohort, we observed notable differences in the incidence of type 2 diabetes (T2D) between PD cases (17.5%) and controls (7.1%), which could bias downstream analyses; therefore, we excluded all individuals with a history of T2D from both groups. We termed this cohort the “UKBB-Sporadic” cohort with 2,237 cases and 167,188 controls. Controls were on average younger than cases at recruitment in this cohort (Table S20), which could introduce bias when investigating signatures of dopamine (DA) neuron degeneration. Thus, for the DA neuron essentiality screen analysis, we employed a super-control design by selecting older controls with a higher median age at recruitment than the cases (Table S20), which are more likely to capture naturally occurring signatures of neurodegeneration and less likely to have sporadic PD later in life. We termed this cohort with super controls as UKBB-Extreme (Ncase = 2,237 and Ncontrol = 2,553).

For the database-wide investigations of BRCA, T2D, CAD, and MI, we created replication cohorts using the jointly genotyped WES data from 53,343 individuals in MGBBB, a biorepository of consented patient samples at Mass General Brigham (parent organization of Massachusetts General Hospital and Brigham and Women’s Hospital). Same inclusion/exclusion criteria were used for each phenotype to ensure consistency between the biobanks. The resulting cohort sizes are as follows: BRCA (Ncase = 1,113; Ncontrol = 2,459), T2D (Ncase = 747; Ncontrol = 2,188), CAD (Ncase = 902; Ncontrol = 1,488), and MI (Ncase = 326; Ncontrol = 2,068). For the BRCA cohort, we used an MAF cutoff of 0.001 to select rare variants. For the other three phenotypes, variants with MAF <0.03 were considered to be “rare”. Since the cohort sizes in MGBBB were smaller compared to UKBB for T2D, CAD, and MI, we did this adjustment to the MAF cutoff and performed tests in the synonymous and neutral missense categories for each pathway to make sure that there was no LD leakage.

For PD, we created a replication cohort using the WGS data from 10,418 individuals from AMP-PD (Accelerating Medicines Partnership: Parkinson’s Disease) v3 release (2022), which encompasses study participants at Mass General Brigham (i.e., Harvard Biomarkers Study 2.0). Any individual belonging to the genetic registry and genetic cohort group, as well as subjects without evidence of dopamine deficit (SWEDD) and subjects belonging to the prodromal categories and the AMP-LBD cohort were excluded from the analysis. As the AMP-PD cohort predominantly consists of individuals of European ancestry, we retained only unrelated individuals of the same ancestry group. We called the resulting cohort “AMP-PD-sporadic,” which consisted of 2,117 sporadic PD cases and 1,095 neurotypical controls. Both case and control groups had similar median ages in this cohort (Table S20).

We retained only unrelated European samples for our analyses because, at the time, individuals of European ancestry constituted the largest group in our study cohorts. Concentrated efforts in building large biobanks with diverse participants are already underway^131^ and will enable NERINE to overcome this limitation and provide more insight into the contribution of rare variants to common disease etiology across populations.

#### H9 ESC culture for DA neuron differentiation

Human H9 (MSKCC stem cell core facility; WA-09) ESCs were cultured in Essential 8 (E8) medium (Gibco/Thermo Fisher Scientific; Cat. No. A1517001) on 10cm plates coated with Vitronectin (Thermo Fisher Scientific; Cat. No. A14700) diluted 1:100 in Dulbecco’s PBS (DPBS; Thermo Fisher Scientific; Cat. No. 14190136) and passaged at ∼80% confluence using StemPro Accutase Cell Dissociation Reagent (Gibco/Thermo Fisher Scientific; Cat. No. A11105-01). E8 medium was replaced every day.

#### Human GM29371 iPSCs culture for CiS-CN differentiation

Human GM29371 iPSCs (Coriell Institute; GM29371∗C) were cultured in Stemflex (Gibco/Thermo Fisher Scientific; Cat. No. A33493) on 6-well plates coated with Matrigel Matrix (Corning; Cat. No. 356231) diluted 1:100 in Knockout DMEM (Gibco/Thermo Fisher Scientific; Cat. No. 10829-018). Essential 8 Medium (Gibco/Thermo Fisher Scientific; Cat. No. A1517001) was replaced every day. When 80% confluent, cells were passaged with StemPro Accutase Cell Dissociation Reagent (Gibco/Thermo Fisher Scientific; Cat. No. A11105-01).

#### Mouse model

Wildtype B6C3F1 mice (The Jackson Laboratories; Stock 100010) were used for the stereotactic injection studies described. Animals were maintained on a 12-h light/dark schedule and provided with food *ad libitum*. All housing, breeding, and procedures were performed according to the NIH Guide for the Care and Use of Experimental Animals and approved by the University of Pennsylvania Institutional Animal Care and Use Committee.

### Method details

#### Overview of the NERINE methodology

NERINE models the “variant-gene-network” hierarchy as follows: it encodes gene-gene relationships in a network of *m* genes by a positive semidefinite matrix, Σ. We assume that phenotypic effects of genes within a network, represented as vector $\vec{\alpha }$, are drawn from a multivariate skew-normal distribution $\vec{\alpha }\;∼\;MSN\left(0,\theta \cdot \Sigma ,\nu \right)$. Here, *θ* is a parameter reflecting the cumulative effect of the gene network on a phenotype and is the object of inference (Figure 1). The skewness parameter *ν* = *f*(*N*_*case*_, *N*_*control*_) adjusts for case-control imbalance. Here, N_case_ and N_control_ represent the case- and control-group sizes. For balanced cohorts (i.e., *ν* = 0), the model reduces to $\vec{\alpha }\;∼\;MVN\left(0,\theta \cdot \Sigma \right)$, where marginals are normally distributed with zero mean. Under this model, an edge between two genes in the network implies that they have either correlated non-zero effect sizes or correlated chances of having no phenotypic effects.

The network-effect, *θ* on a dichotomous phenotype is estimated using the maximum likelihood estimation (MLE) framework, where the likelihood is modeled as an integral over two components: (i) the product of conditional probabilities of observed case mutation counts given total mutation counts in network-genes, and (ii) the probability of gene effects given the network parameters:

$$L\left(\theta |X,Y,\vec{\alpha },\Sigma ,N_{case},N_{control}\right)=\int \left(\prod_{i}P\left(X_{i}|X_{i}+Y_{i},\alpha_{i}\right)\right)P\left(\vec{\alpha }|\theta ;\Sigma ,\nu \right)d\vec{\alpha }$$

We approximate this integral as a weighted sum over multivariate quadrature points from the domain of integration. The weight of each multivariate quadrature point is given by the product of the corresponding univariate weights. To make the computation of the integrand tractable, NERINE makes several design choices and employs a lookup table approach with pruning (Methods S2-S4).

NERINE models rare variant counts in genes for cases and controls as two independent Poisson random variables as follows,

Allele counts in cases in gene *i*, $X_{i}\;∼\;Poisson\left(N_{case},\lambda_{case}^{i}\right)$

Allele counts in controls in gene *i*, $Y_{i}\;∼\;Poisson\left(N_{control},\lambda_{control}^{i}\right)$

Here, $\lambda_{case}^{i}$ and $\lambda_{control}^{i}$ denote the rate parameters for case- and control-allele count distributions in gene *i*, and correspond to population allele frequencies renormalized between cases and controls by transformed network-gene effect, *α*_*i*_. This implies that the conditional probability of observed rare variant count in each gene in cases, given the total rare variant count in the cohort, follows a Binomial distribution (Methods S2).

$$P\left(X_{i}=k|X_{i}+Y_{i}=n\right)=\left(\begin{array}{c} n\\ k \end{array}\right){\left(\frac{\lambda_{case}^{i}}{\lambda_{case}^{i}+\lambda_{control}^{i}}\right)}^{k}{\left(\frac{\lambda_{control}^{i}}{\lambda_{case}^{i}+\lambda_{control}^{i}}\right)}^{n-k}\approx Binom\left(n,p\right)$$

where *p* = *ϕ*(*α*_*i*_).

Since each *α*_*i*_ represents unbounded univariate skew normal distributions, they cannot be directly used as Binomial success probabilities. Hence, we apply a custom variable transformation to map *α*_*i*_s to Beta distributions so that they fall on the interval [0, 1] (Methods S3). This transformation ensures that the mean gene effect is centered around *N*_*case*_/(*N*_*case*_+*N*_*control*_), and the shape of the transformed distribution adapts with *θ*: small *θ*s (close to 0) concentrate density near the mean, while large *θ*s shift density toward the extremes. Case-control skew is incorporated using the shape parameters of the Beta distribution. The transformed gene-effects within the network are denoted $\vec{\alpha^{\prime }}$ (Methods S3).

Thus, NERINE’s approximate likelihood takes the form:

$$L\left(\theta |X,Y,\vec{\alpha },\Sigma ,N_{case},N_{control}\right)\approx \underset{\vec{\alpha^{'}}}{\sum }\left(\prod_{i=1}^{m}P\left(X_{i}|X_{i}+Y_{i},\alpha_{i}^{'}\right)\right)\cdot P\left(\vec{\alpha^{'}}|\theta ;\Sigma ,\nu \right)$$

The conditional probability term is calculated using the probability density function of a standard Binomial distribution, which makes the likelihood computation fast and tractable. To calculate the probability of network-gene effects ($\vec{\alpha^{\prime }}$) for a given network topology (Σ) and network effect (*θ*), we use a lookup table, where highly improbable $\vec{\alpha^{\prime }}$ s are pruned (Methods S4).

NERINE performs nested hypothesis testing; the null hypothesis being *H*_0_:*θ* = 0 (i.e., the gene network does not affect the trait and expected ratio of variant counts between cases and controls is proportional to the ratio of sample sizes in each gene) and the alternative hypothesis being *H*_1_:*θ* > 0 (i.e., the gene network has an overall effect on the trait and the ratio of variant counts between cases and controls may deviate from the null expectation in relevant member genes). The test statistic of NERINE is the log likelihood ratio (LLR):

$$LLR=2\times \left(\log\left(L\left(H_{1}\right)\right)-\log\left(L\left(H_{0}\right)\right)\right)$$

We denote the maximum-likelihood estimate of network effect with $\hat{\theta }=\underset{\theta }{\mathrm{argmax}}\left(LLR\right)$. Since *θ* = 0 is on the boundary of the parameter space, the test statistic asymptotically follows the distribution of a weighted mixture of a point mass at zero and a chi-square distribution with a degree of freedom of one (*dof* = 1).^30^^,^^31^ NERINE draws its asymptotic *p*-values from this distribution. For significant networks, NERINE calculates the maximum likelihood gene-specific effects ($\hat{\vec{\alpha^{\prime }}}$) under the estimated $\hat{\theta }$ as follows:

$$\hat{\vec{\alpha^{'}}}=\underset{\vec{\alpha^{'}}}{\mathrm{argmax}}L_{\hat{\theta }}\;\approx \;\underset{\vec{\alpha^{'}}}{\mathrm{argmax}}\left(\left(\prod_{i=1}^{m}P\left(X_{i}|X_{i}+Y_{i},\alpha_{i}^{'}\right)\right)P\left(\vec{\alpha^{'}}|\hat{\theta };\Sigma ,\nu \right)\right)$$

The search space for possible network-gene effects ($\vec{\alpha^{\prime }}$) is determined by the estimated $\hat{\theta }$, the network structure (Σ), and the lookup table entries.

#### Simulations under the null model

To evaluate the performance of NERINE under the null model (*θ* = 0), we performed extensive simulations with well-studied biological pathways, namely, NOTCH pathway (m = 6), WNT pathway (m = 24), protein export (m = 24), and EGFR signaling (m = 50), from the canonical pathways database (Figures S1 and S2) and different simulated network architectures—(i) clique: complete graph with all nodes connected to each other; (ii) path: each node connected to two other nodes except the first and the last nodes; (iii) random: randomly generated scale-free graph of *m* nodes; and (iv) isolated genes: nodes not connected with each other (Figure S3).

For the canonical pathways, gene lists were extracted from MSigDB (v7.3) and high confidence physical and genetic interactions from protein-protein interaction (PPI) databases were used as network edges between pathway genes. We simulated three different scenarios – (i) equal sized case/control groups (*N*_*case*_ = 1,000;*N*_*control*_ = 1,000) (ii) case group is larger (*N*_*case*_ = 3,000;*N*_*control*_ = 1,000), and (iii) control group is larger (*N*_*case*_ = 1,000;*N*_*control*_ = 3,000).

Under different network architectures and case-control skews, we simulated allele counts in cases and controls using independent Binomial distributions under the null model. For each gene, we assumed the presence of up to five qualifying loci each with minor allele frequency (MAF) of 0.001. The binomial probabilities for case- and control-groups are adjusted according to the group sizes and MAF of variants assuming no gene-specific effects under the null model (*θ* = 0). For each scenario, we performed 1,000 iterations to generate the QQ-plots.

We used the *pchibarsq* function from the *emdbook* (v1.3.13) in R (v4.3.2) to calculate the *p*-values from the mixture of chi-square distribution with 1° of freedom (*dof* = 1) and the delta function at zero (0). We calculated 95% bootstrap confidence intervals around NERINE’s test-statistic for visualization.

For evaluating the null behavior of NERINE’s test statistic in simulated networks of different sizes (*m* = 5, 10, and 25 genes) and different topological architectures (i.e., clique, path, random, and isolated nodes), we used equal sized case- and control-groups (Figure S3). For each scenario, we performed 1,000 iterations to generate the QQ-plots. The allele counts in cases and controls were generated from independent Binomial distributions following the same procedure as above.

#### Performance benchmarking with simulated data

We evaluated the performance of NERINE under the alternative hypothesis (*θ* > 0) in two sets of simulations—(i) when genes have only trait-increasing effects, and (ii) when genes have both trait-increasing and trait-decreasing effects using the same database pathway network topologies used for the null simulations (Figure 2; Figure S4). For each scenario, we simulated different noise profiles, i.e., varying proportions of genes within the network with effects on the trait given the network topology. This mimics situations from having a very noisy network (i.e., ∼10–30% genes with an effect on the trait) to a highly relevant network (i.e., ∼70–90% genes with an effect on the trait). We simulated allele counts from cases and controls using independent Binomial distributions under the alternative model with *θ* = 0.2. For each gene, we assumed the presence of up to five (5) qualifying loci per gene with minor allele frequency (MAF) of 0.001. The Binomial probabilities for case- and control-groups are adjusted according to the group sizes and MAF of variants assuming possible gene-specific effect configurations under the alternative model (i.e., $\vec{\alpha }$ given the network topology (Σ) and network effect, *θ* = 0.2). Using this setup, we simulated a cohort of 2,000 cases and 2,000 controls.

Currently, there are no existing rare variant association tests that take gene network topology into account. Thus, we compared the performance of NERINE with existing gene-level rare variant association tests adapted to the pathway level, namely, CMC-Fisher test,^132^ Fisher minimum *p*-value test,^133^ Fisher combined test,^133^ SKAT-O,^18^ and pathway-based rare variant trend test (RVTT).^15^^,^^16^ For each noise profile, we performed 250 iterations, resulting in 1,000 iterations per network. Empirical power of each method was measured as the positive predictive value (PPV) across iterations using different *p*-value cutoffs (*c*): 1 × 10^−2^, 5 × 10^−3^, 1 × 10^−3^, 5 × 10^−4^, 1 × 10^−4^, 5 × 10^−5^, and 1 × 10^−5^. Here,

$$PPV=\frac{\#\;\text{Positive}\;\text{findings}\;\text{with}\;p-\text{value}<c\;}{\text{total}\;\#\;\text{of}\;\text{test}\;\text{cases}}$$

Additionally, we conducted performance benchmarking for NERINE on different simulated network architectures for 25 genes (Figure S5). As before, we performed two sets of simulations—(i) when genes have only trait-increasing effects, and (ii) when genes have both trait-increasing and trait-decreasing effects for four network topologies—clique, path, random graph, and isolated nodes. We simulated allele counts in 1,000 cases and 1,000 controls using independent binomial distributions under the alternative model with a different network effect, *θ* = 0.5. We simulated various network-noise profiles ranging from 0 to 90% following the same procedure as above. Empirical power of each method was calculated as PPV across 250 iterations per noise profile per network topology, resulting in 1,250 iterations per network. Since RVTT, by design, assumes that all qualifying rare variants in a pathway have the same direction of effects, we compared RVTT’s performance with NERINE in simulations with genes having only trait-increasing effects. Moreover, RVTT computes a permutation-based *p*-value, and for 10,000 iterations its *p*-values cannot be less than 1e−4. Hence, we compared RVTT’s performance only at cutoff values ≥ 1e−4.

Furthermore, we evaluated NERINE’s performance with true versus randomized network architectures in simulations with the same four well-studied pathways (Figure S10). We constructed the “true” network topologies for these pathways using the high-confidence protein–protein and genetic interactions from PPI databases as described above. For each gene set, we also generated 100 randomized networks by randomly introducing edges among the member genes. A binary trait was simulated in 2,000 cases and 2,000 controls with varying trait-increasing and trait-decreasing effects under the alternative model with a network effect, *θ* = 0.1. We simulated different noise profiles (i.e., 10–30%, 30–50%, 50–70%, and 70–90%) and performed 250 iterations per noise profile. We measured NERINE’s PPV at different *p*-value thresholds (1 × 10^−2^, 5 × 10^−3^, 1 × 10^−3^, 5 × 10^−4^, 1 × 10^−4^, 5 × 10^−5^, and 1 × 10^−5^).

#### Pathway database construction

We created a pathway database with all canonical pathways of five to fifty genes from the BIOCARTA database along with all lipid-, DNA replication-, DNA damage repair-, and cell cycle-related pathways from the REACTOME, KEGG, PID, and Wiki pathways databases. The lists of member genes for these pathways were extracted from the Molecular Signatures Database (MSigDB v7.3; https://www.gsea-msigdb.org/gsea/msigdb). The database contained 306 pathways with a median pathway length of 25 genes (Table S21).

#### Gene-network topology extraction

NERINE treats the gene-gene network as an input and can flexibly handle any symmetric pairwise relationship matrix that is positive-semidefinite. For a screen that provided the gene network topology, we used that network as is. For example, for the *α*-synuclein proteotoxicity screen in PD, we directly applied NERINE on the published TransposeNet humanized *α*-synuclein-modifier network^2^ stems. In absence of the true network topology for a particular gene set, we adopted the following approach to construct one.

For canonical database pathway gene sets, we constructed network topologies by extracting physical and genetic interactions from several sources: (i) high confidence physical interactions (weight ≥ 0.7) from STRING v11.5,^119^ (ii) InWeb inBio Map database,^111^ (iii) HuRI,^6^ (iv) genetic interactions from the Megchelenbrink et al. study,^112^ and (v) humanized *α*-synuclein-, *β*-amyloid-, and TDP-43-modifier networks.^2^ Using a heuristic approach, we first generated adjacency matrices for the genes, where edges were represented as binary values (1 = presence of an edge; 0 = absence of an edge), corresponding to the non-zero off-diagonal entries of the network. The diagonal was set to two (2) to indicate an equal prior on each gene. Because these modified adjacency matrices were not always positive semidefinite, we applied the *nearPD* function^123^ from the *Matrix* package (v1.6-5) in R (v4.3.2) to compute the nearest positive-definite matrix. This matrix was input to NERINE as an approximate covariance matrix, Σ.

We also constructed co-expression networks in relevant tissue types for different phenotypes using the bulk tissue expression data from the Genotype Tissue Expression database^113^ (GTEx v8). We computed a real-valued gene-gene co-expression networks in a specific tissue where the edges between two genes represented the Pearson correlation of their expression profiles in that tissue types. For lipid-related phenotypes, we constructed co-expression networks using the bulk expression data from liver tissue. For Parkinson’s disease (PD), we used bulk expression data from the substantia nigra region of the mid-brain.

To construct co-essentiality networks in relevant cell lines for different phenotypes, we used the gene dependency data from CRISPR knockout screens from project Achilles, as well as genomic characterization data from the Cancer Cell Lines Encyclopedia (CCLE) project from the DepMap portal^114^^,^^134^ (v2023Q2). We computed a gene-gene co-essentiality networks in relevant cell lines where the edges between two genes represent the Pearson correlation of their dependency profiles in those cell lines. While for lipid-related phenotypes, we constructed co-essentiality networks using data from liver cell lines, for PD, we used data from cell lines pertaining to the central nervous system (CNS). The cell lines used in this study are listed in the Table S22.

#### Performance benchmark on UKBB lipid phenotypes

NERINE was competitively applied on two binarized LDL-C and HDL-C phenotypes in UKBB across networks from our canonical pathways database. We extracted the edge relationships of genes from high-confidence physical and genetic interactions from protein interaction databases for this exercise. This analysis served as an ideal positive control because the genetic determinants of these phenotypes are well-annotated. Variants with minor allele frequency (MAF) < 0.001 were considered rare for the test to not be influenced by artificial signal from common variant space propagated through linkage disequilibrium (LD). We stratified variants into six functional categories: LoF (i.e., frameshifts, insertions, deletions, and splice variants), damaging missense (i.e., missenses predicted to be damaging by in-silico tools), damaging (i.e., LoF and damaging missenses), missense, neutral (i.e., missenses predicted to be benign by in-silico tools), and synonymous. We used neutral and synonymous variants in a pathway as control to safeguard against technical biases and LD leakage. Bonferroni correction, accommodating the presence of correlated hypotheses in the database,^135^ was used to control for Type I error.

To ensure our results for LDL-C phenotype were not driven by *LDLR* and *PCSK9*, which have large individual effect sizes on LDL-C levels, we performed sensitivity analysis by removing *LDLR* and *PCSK9* from the significant networks for the LDL phenotype. Even without *LDLR* and *PCSK9*, the module of core LDL-related genes, along with the LDL clearance and chylomicron clearance pathways remained significant for the LDL phenotype after Bonferroni correction (Figure S22).

For many disease phenotypes, the large sample sizes available for the UKBB lipid phenotypes, are simply not attainable. Thus, we performed a down-sampling experiment to evaluate the consistency of NERINE’s performance across different sample sizes and the robustness of NERINE’s performance with small sample sizes. We downsampled the high LDL vs. low LDL cohort at different case-control ratios (1/3, 1/10, and 1/60) and competitively applied NERINE across the pathway database, demonstrating NERINE’s effectiveness even in a cohort with as few as 500 cases and 500 controls (Figure S23).

Since rare variants are more susceptible to subtle effects of population stratification than common variants, we performed stratified analysis of the LDL-C phenotype in all five major ancestry groups (i.e., European, American, African American, South Asian, and East Asian) and meta-analyzed the results using Fisher’s combined test (Figure S9; Table S2). Bonferroni correction was applied on the combined *p*-values.

We further evaluated NERINE on real versus randomized network topologies for binarized LDL-C and HDL-C phenotypes (Figure S11). For each phenotype, we selected database-wide significant pathways with rare damaging variant burden and used the most significant topologies for each pathway shown in Table S3 as “real” network topologies. We also generated 100 randomized networks per pathway by introducing random binary edges among member genes. We applied NERINE to both real and randomized networks and assessed its performance by comparing LLRs and *p*-values; higher LLRs and lower *p*-values indicated superior performance.

#### Variant and sample quality control in cohorts

We performed both variant- and sample-level quality control (QC) steps on each sequencing dataset to ensure the study cohorts are free from technical biases as much as possible (see Methods S5 for detailed steps). We retained only high-quality biallelic variants passing GATK best practices filters and having maximum 10% missingness for our analysis. We removed sample outliers based on Ts/Tv, Het/Hom ratios, and per-haploid SNV counts, where outliers were defined as samples that were three standard deviations away from the mean.

We annotated variants with their functional consequences and gnomAD allele frequencies with VEP (v109) and dbNSFP (v4.3a) database. We used six masks to group variants into functional categories: (i) *Damaging missense*: missense variants predicted to be either ‘‘P’’ or ‘‘D’’ by PolyPhen2 (v2.2.3) or ‘‘deleterious’’ by SIFT (v6.2.1), (ii) *LoF*: variants labeled as splice donors, splice acceptors, splice region variants, stop-gained, stop-lost, start-lost, frameshifts, in-frame insertions, and in-frame deletions; (iii) *Damaging*: LoFs and damaging missenses, (iv) *Missense*, (v) *Neutral*: missense variants predicted to be either ‘‘B’’ by PolyPhen2 or ‘‘tolerated’’ by SIFT, and (vi) *Synonymous*. These masks were used in our analysis of binarized cholesterol phenotypes as well as complex diseases.

Notably, NERINE is agnostic to the variant annotation tool and can test any user-defined categories of rare variants. To assess robustness, we reclassified variants using REVEL^125^ (version May 2021) and AlphaMissense^121^ scores (hg38 v1) within the VEP (v109) plugin and custom annotation tools in a positive control experiment with the LDL-C phenotype in UKBB. We defined three categories: (i) *pathogenic missense* (REVEL >0.649 or AlphaMissense >0.564), (ii) *pathogenic* (pathogenic missense plus LoF), and (iii) *benign missense* (REVEL <0.29 and AlphaMissense <0.34). Results were highly concordant with those obtained using PolyPhen2 and SIFT (Figure S24).

#### DA neuron differentiation

Human WA-09 ESCs harboring a doxycycline-inducible Cas9 cassette in the AAVS1 safe harbor locus were differentiated following a previously published protocol.^136^ Briefly, ESCs were dissociated and plated at high density (600,000 cells/cm^2^) in Neurobasal medium (Gibco/Thermo Fisher Scientific; Cat. No. 21103-049) supplemented with 1X N2 supplement (Life Technologies; Cat. No. 17502048), 1X B27 supplement (Life Technologies; Cat. No. 17504044), 2 mM L-glutamine (Gibco/Thermo Fisher Scientific; Cat. No. 25030-081), penicillin-streptomycin (Gibco/Thermo Fisher Scientific; Cat. No. 15140122), 250 nM LDN193189 (Reprocell; Cat. No. 04–0074), 10 μM SB431542 (R&D Systems; Cat. No. 1614), 1 μM CHIR99021 (R&D Systems; Cat. No. 4432), 500 ng/mL SHH (R&D Systems; Cat. No. 464-SH), and 10 nM Y-27632 ROCK inhibitor (Bio-Techne; Cat. No. HY-10583). Cells were counted and plated on DMEM/F12 (Gibco/Thermo Fisher Scientific; Cat. No. 11320033) and Geltrex-coated plates (Life Technologies; Cat. No. A1413201) at high density (600,000 cells/cm^2^), while being maintained in this medium from DIV 0 (day of plating) to DIV 3. Y-27632 ROCK inhibitor was added only at plating. Medium change was performed on DIV 4 and every three days till DIV 9. On DIV 4, the medium was supplemented with 6μM CHIR99021. On DIV 7, the medium was composition was changed to exclude SB431542 and SHH. From DIV 10, cells were transitioned to maturation medium with the following composition: Neurobasal Medium as the base, 1X B27 Supplement, 2mM L-glutamine, penicillin-streptomycin, 3μM CHIR99021, 0.2mM Ascorbic Acid (Sigma-Aldrich; Cat. No. A4034), 20ng/mL GDNF (Gibco/Thermo Fisher Scientific; Cat. No. 450-10), 1ng/mL TGFβ3 (Gibco/Thermo Fisher Scientific; Cat. No. 100-36E), 20ng/mL BDNF (R&D Systems; Cat. No. 248-BDB), and 0.2mM dbcAMP (Sigma-Aldrich; Cat. No. 4043). For DIVs 10 and 11, cells were maintained in this medium.

On DIV 11, partially differentiated cells were released and centrifuged as above, and pelleted cells were resuspended in the same medium from DIVs 10–11 and plated at high density (800,000 cells/cm^2^) on 15μg/mL Poly-L-Ornithine (Sigma-Aldrich; Cat. No. P3655), 2μg/mL Fibronectin (Gibco/Thermo Fisher Scientific; Cat. No. 356008), and 1μg/mL Laminin (R&D Systems; Cat. No. 3400-010-03)-coated plates in DPBS. From DIVs 12 to 15, cell were placed into maturation medium with a composition similar to DIVs 10–11 medium supplemented with 1 μM IWP-2 (Tocris Bioscience; Cat. No. 3533) and 100 ng/mL FGF-18 (PeproTech; Cat. No. 100-28). Doxycycline hydrochloride (2 μg/mL; Sigma-Aldrich; Cat. No. D3447) was added on DIVs 14 and 15 to induce Cas9 expression. On DIV 16, partially differentiated cells were released and centrifuged as above, and pelleted cells were replated at 1,200,000 cells/cm^2^ and resuspended in maturation medium with a composition similar to DIVs 10–11 medium supplemented with 10 μM DAPT (R&D Systems; Cat. No. 2634) for ten days. On DIV 25, neurons were dissociated and replated at low density (200,000 cells/cm^2^) and maintained in maturation medium containing 15μg/mL Poly-L-Ornithine, 2μg/mL Fibronectin, and 1μg/mL Laminin-coated plates in DPBS.

#### Genome-wide CRISPR-Cas9 screen in DA neurons

We performed genome-wide CRISPR-Cas9 screen in DA neurons differentiated from human WA-09 (H9) embryonic stem cells as described above. The Gattinara human CRISPR pooled knockout library^130^ (Addgene; pooled library #136986) was used for this screen; this library includes two gRNAs for 19,993 genes as well as 500 non-targeting controls and 500 controls targeting one intergenic. Guide RNAs (gRNAs) representing 19,993 genes were transduced into H9 hESC lines at an MOI of 0.3–0.5, maintaining ∼1000x library coverage. Transduced stem cells were selected by puromycin and differentiated toward DA neurons until reaching the neural progenitor stage as described by Kim and colleagues.^137^ Briefly, at DIVs 14–16 of the differentiation, we induced iCas9 expression by doxycycline addition using the AAVS1 safe-harbor locus as previously described,^138^ while cells were neural progenitors. Then, we waited until DIV25 when they differentiated into DA neurons to take our initial sample to obtain gRNA representation through next-generation sequencing (DIV26). The remaining neurons were allowed to stay in the dish until our final collection time (DIV42) when neuronal cell death began. Samples were processed for library preparation and sequenced and sequencing reads were aligned to the screened library. The complete screen will be described in a forthcoming publication.

#### Constructing ontology-based network topologies

For PD GWAS genes, we identified six GO biological process modules (Table S12) and for DA neuron essentiality genes, we identified 10 such modules (Table S15) using gene over-representation analysis (see quantification and statistical analysis). To impose network topology on these gene modules, we extracted edge relationships of genes in each group from three different data sources as described above: (i) high-confidence physical and genetics interactions from protein interaction databases, (ii) co-expression in the substantia nigra region of the mid-brain from GTEx v8, and (iii) co-essentiality in CNS cell types in DepMap (v2023Q2).

We also explored an alternative approach from a recent study^65^ for constructing gene modules from PD GWAS loci, which involved running MAGMA^139^ analysis on GWAS genes, followed by GO BP enrichment. This approach identified 21 significant conditionally independent GO terms^65^ (Table S23). We imposed network topology on the genes within each GO module by extracting gene-gene relationships from the sources described above.

#### Induced neuron (CiS-CN) differentiation

Human GM29371 iPSCs engineered to express *NGN2* under a doxycycline-inducible system in the AAVS1 safe harbor locus were differentiated following previously published protocol.^140^

Briefly, iPSCs were released, centrifuged, and resuspended in N2 Pre-Differentiation Medium containing the following: Knockout DMEM/F12 (Gibco/Thermo Fisher Scientific; Cat. No. 12660-012) as the base, 1X MEM Non-Essential Amino Acids (Gibco/Thermo Fisher Scientific; Cat. No. 11140-050), 1X N2 Supplement (Gibco/Thermo Fisher Scientific; Cat. No. 17502-048), 10ng/mL NT-3 (PeproTech; Cat. No. 450-03), 10ng/mL BDNF (PeproTech; Cat. No. 450-02), 1 μg/mL Mouse Laminin (Thermo Fisher Scientific; Cat. No. 23017-015), 10nM ROCK inhibitor (Peprotech; Cat. No. 1293823), and 2μg/mL doxycycline hydrochloride (Sigma-Aldrich; Cat. No. D3447-500MG) to induce expression of mNGN2. iPSCs were counted and plated on Matrigel-coated plates in N2 Pre-Differentiation Medium for three days.

After three days, hereafter DIV 0, pre-differentiated cells were released and centrifuged as above, and pelleted cells were resuspended in Classic Neuronal Medium containing the following: half DMEM/F12 (Gibco/Thermo Fisher Scientific; Cat. No. 11320-033) and half Neurobasal-A (Gibco/Thermo Fisher Scientific; Cat. No. 10888-022) as the base, 1X MEM Non-Essential Amino Acids, 0.5X GlutaMAX Supplement (Gibco/Thermo Fisher Scientific; Cat. No. 35050-061), 0.5X N2 Supplement, 0.5X B27 Supplement (Gibco/Thermo Fisher Scientific; Cat. No. 17504-044), 10ng/mL NT-3, 10ng/mL BDNF, 1μg/mL Mouse Laminin, and 2μg/mL doxycycline hydrochloride. Pre-differentiated cells were subsequently counted and plated on BioCoat Poly-D-Lysine coated plates (Corning; Cat. No. 356470) in Classic Neuronal Medium. On DIV 7 and each week after, medium change was performed without doxycycline added. In the PFF exposure experiment, a complete medium change to the medium containing 10 μg/mL synthetic PFF was performed on DIV 21 neurons, and the neurons were fixed at DIV28 for immunostaining.

#### CRISPRi screen in the CiS-CN model

A customized CRISPRi library comprising 9,852 sgRNAs targeting 1,705 physical and genetic interactors of α-synuclein (*αS*)—including genes from the TransposeNet *αS* proteinopathy network—along with negative controls, was constructed. The library was packaged into lentivirus by the Virus Core at Boston Children’s Hospital. Neurons were transduced with the viral library and cultured until the harvesting date (DIVs 3, 28, and 42). Genomic DNA was extracted from harvested neurons, sgRNA-encoding regions were PCR-amplified, and sgRNA abundance was quantified by next-generation sequencing based on previously described protocols.^140^^,^^141^^,^^142^ Sequencing reads were aligned to the reference sgRNA library. Sequencing and annotation were conducted at Memorial Sloan Kettering Cancer Center. Details of the functional screen in its entirety will be described in a forthcoming publication from our group.

#### Immunostaining and microscopy imaging

Neurons are fixed with 4% PFA (EM Sciences; Cat. No. 15710) for 15 min at room temperature, and then permeabilized with 0.5% Triton X-100 (Sigma-Aldrich; Cat. No. T8787) and blocked with 0.05% Triton X-100 and 5% BSA (Sigma-Aldrich; Cat. No. A7906) in DPBS (Thermo Fisher Scientific; Cat. No. 14040182) for 1h at room temperature. Samples were incubated with primary antibodies (PRL: Thermo Fisher Scientific; Cat. No. MA1-10597; 1:200 dilution) at 4°C overnight, followed by incubation with secondary antibody and Hoechst 33342 (Thermo Fisher Scientific; Cat. No. H3570; 1:2000 dilution) for 1h at room temperature. Images were captured with identical settings for parallel cultures using Nikon Eclipse Ti microscope or Nikon TiE/C2 confocal microscope. Image analysis was performed with ImageJ2^129^ Macro Software (Methods S6). *Prl* level was determined by D (D = total *prolactin* intensity/total DAPI number in a given image).

#### Oxidative stress assay

DIV0 CiS neurons were seeded at a density of 40,000 cells/well of poly-L-ornithine-coated 96-well plate (solution: Sigma-Aldrich; P4957). At DIV6, the neuron media was fully changed for 100uL *Prolactin*-containing (Thermo Fisher Scientific; Cat. No. 100-07-10UG) media at a concentration of 0,1 or 10 nM. 24h post-treatment, the neuron media was fully changed for 100uL of Menadione-containing (Thermo Fisher Scientific; Cat. No. ICN10225925) media at a concentration of 0 or 100uM and incubated for an hour at 37°C. After an hour, CELLROX Green Reagent (Thermo Fisher Scientific; Cat. No. C10444) was added on top of the Menadione-treated media to a final concentration of 5 μM CELLROX for 30 min at 37°C. All media was then removed, and wells were washed 3 times with PBS. After the third wash, the PBS was replaced with neuron media. The plates were then taken to the Incucyte S3 live-cell analysis system (Sartorius) for imaging. Incucyte analysis was perform with S3 software, and CellRox = total integrated intensity/neuron was reported.

#### *αS* and PFF preparation

Full-length mouse ɑS was expressed in BL21 (DE3) RIL-competent E. coli cells (Agilent Technologies; Cat. No. 230245) transformed with pRK172/mSyn containing ɑS cDNA.^29^ Protein purification was previously described.^29^^,^^143^ Cultures expanded in Terrific Broth (Sigma-Aldrich; Cat. No. T9179, composition: 12 g/L of Bacto-tryptone, 24 g/L of yeast extract 4% (vol/vol) glycerol, 17 mM KH2PO4 and 72 mM K2HPO4) containing ampicillin (Fisher Scientific; Cat. No. BP1760) were harvested and sonicated in high salt buffer (750 mM NaCl in 10 mM Tris, pH 7.6; NaCl: Fisher Scientific, Cat. No. S271; Tris: Fisher Scientific, Cat. No. BP152). After boiling for 15 min, the supernatant was dialyzed against 10 mM Tris, pH 7.6, 50 mM NaCl, 1 mM EDTA (Sigma-Aldrich; Cat. No. E5134) overnight at 4°C, filtered and concentrated using Amicon Ultra-15 centrifugal filter units (Merck Millipore; Cat. No. UFC901008). Gel filtration using a Superdex 200 column (Cytiva; Cat. No. 17517501) was performed and fractions containing ɑS pooled and dialyzed in 10 mM Tris, pH 7.6, 50 mM NaCl, 1 mM EDTA overnight. The product was polished using a HiTrapQ HP column (Cytiva; Cat. No. 645932) and eluted over an ionic gradient (25–1,000 mM NaCl). Fractions containing ɑS were combined and dialyzed into DPBS (Invitrogen; Cat. No. 14200075), sterile filtered and concentrated to 5 mg/mL and frozen at −80°C until used. PFFs were assembled by shaking monomer at 5 mg/mL using a Thermomixer C (Eppendorf) set at 1,000 rpm for 7 days at 37°C. Fibril content was validated by sedimentation at 100,000 x g for 30 min and Thioflavin T fluorimetry.

#### Stereotaxic administration of PFFs

Prior to injection, PFFs were diluted to 2 mg/mL in DPBS and sonicated using a bath sonicator (Diagenode; Biorupter UCD-300) on high power for 10 cycles (30 s on; 30 s off) at 10°C. Each mouse received a single unilateral injection of PFFs (5 μg of PFFs in 2.5 μL volume) into the dorsal striatum using a Hamilton syringe (33 gauge) using the following co-ordinates: AP +0.2 mm relative to bregma; ML +2.0 mm; depth 2.6 mm beneath the dura. DPBS injected into the same region was used as a negative control. Mice were perfused transcardially with heparinized PBS at 30 d.p.i. and brains flash frozen at −80°C until use.

#### RNA isolation and NanoString analysis

The amygdala region ipsilateral to PFF- or PBS injection was microdissected from each brain and homogenized in 1 mL of TRI Reagent (Sigma-Aldrich, Cat. No. T9424) using TissueRuptor II (Qiagen, Cat. No. 9002755) with a disposable probe (Qiagen, Cat. No. 990890). RNA was then isolated using Direct-zol RNA MiniPrep kit with in-column DNaseI treatment (Zymo; Cat. No. R2050). Samples were quantitated with a NanoDrop 1000 Spectrophotometer (Thermo Fisher Scientific) and assayed for RNA integrity on a 4200 TapeStation (Agilent; Cat. No. G2991AA). NanoString hybridization of the resultant RNA was carried out for a constant 18 h at 65°C on the *Mus musculus* Neuropathology panel (v1.0). Post-hybridization processing in the nCounter Prep Station used the High Sensitivity settings. The cartridge scanning parameter was set at high (555 FOV). RNA isolation and NanoString studies were performed at the Wistar Genomics core facility.

### Quantification and statistical analysis

#### Multiple hypotheses correction for pathways

Due to the pleiotropy of genes, many biological pathways tend to overlap significantly with each other. Thus, we determined the effective number of independent hypotheses (*t*_*eff*_) in our pathway database of *t* pathways by adapting Nyholt’s approach.^135^

First, we calculated the pairwise Jaccard similarity of pathways, and encode it in a matrix, *M*_*t*×*t*_, which is symmetric but not positive semi-definite. We then converted it to its nearest positive definite matrix using the *nearPD* function^123^ from the *Matrix* package (v1.6-5) in R (v4.3.2). Next, we determined the eigenvalues ($\vec{\lambda }$) of this approximate covariance matrix. The effective number of hypotheses/pathways was computed using the following formula^135^:

$$t_{eff}=1+\left(t-1\right)\left(1-\frac{Var\left(\vec{\lambda }\right)}{t}\right)$$

For our pathway database, we found the effective number of independent hypotheses, *t*_*eff*_ to be 300, which was used for Bonferroni correction to determine database-wide significance.

#### Data analysis for DA neuron essentiality screen

Sequencing reads were aligned to the screened library, and the CRISPR screen data were analyzed using the *MAGeCK-MLE* pipeline from the *MAGeCKFlute*^126^ (v2.6.0) package in R (v4.3.2). Genes with Wald test FDR-adjusted *p*-value <0.05, and beta < −0.58 were classified as DA neuron essentiality genes. Here, beta represents the effect size (positive: sgRNA enriched, negative: sgRNA depleted). After removing broadly essential genes^126^ that are not specific to DA neurons, the screen identified 693 genes essential for DA neuron survival. These genes were used to construct the GO modules, which were tested as network hypotheses in the PD analysis.

#### GO over-representation analysis of gene sets

For PD GWAS genes as well essential genes for DA neuron survival, we first performed gene set over-representation analysis using the *enrichr* function from the *GSEApy* package (v 1.1.3) in python (v 3.12.4) and identified all GO biological processes with nominal significance (*p*-value <0.05). We then grouped semantically similar GO terms using REVIGO^128^ (http://revigo.irb.hr/) to identify GO biological process (BP) modules with minimal overlap. We only kept modules of 10 or more genes for our analysis. For PD GWAS genes, we identified six such modules (Table S12) and for DA neuron essentiality genes, we identified 10 such modules (Table S15).

#### Data analysis for CiS-CN CRISPRi-screen

Screen data was analyzed using the *MAGeCK-iNC* pipeline^140^ from the *MAGeCK* package (v0.5.9.2) using python (v2.7). Hits were classified as having an FDR-adjusted *p*-value <0.1, and the gene product cutoff was selected by the *MAGeCK-iNC* pipeline dynamically for each comparison. Hits with significant sgRNA dropout were termed “toxicity enhancers.” The complete list of hits will be described in a forthcoming publication from our lab.

#### Differential expression analysis in mouse model

Raw data from the PFF vs. PBS mouse model experiment were analyzed using Nanostring’s nCounter module provided by Rosalind (Rosalind.bio). Genes with hybridization counts ≥20 were normalized to the geometric mean of the positive controls of the *Mus musculus* neuropathology panel as recommended by the manufacturer. Differential expression analysis was performed using a generalized linear model in the *nCounter* module, which assumes a negative binomial distribution and estimates noise and dispersion across all samples from raw data. Adjusted *p*-values were calculated using the Benjamini-Hochberg method using treatment (i.e., PFF vs. PBS).

## Supplemental information

Document S1. Figures S1–S26, Tables S1, S4, S6, S12, S15, and S20, and Methods S1–S6

Table S2. Network-level rare variant burden across our pathway database for the binarized LDL-C phenotype (high vs. low) across five major ancestry groups in the UK Biobank, related to STAR Methods and Figure S9 Fisher’s combined test was used to meta-analyze the *p* values, and Bonferroni correcting with *t*_*eff*_ = 300 was used for multiple hypotheses correction.

Table S3. Competitively selecting network topologies from different data sources with NERINE, related to Figure 3 and STAR Methods Network topologies were constructed from three data sources: (1) high-confidence experimentally derived physical and genetic interactions of proteins from canonical databases, (2) gene-gene co-expression in liver tissue in GTEx (v8), and (3) co-essentiality of genes in liver cell lines in DepMap (v2023Q2) for lipid-related pathways (STAR Methods). Each topology was tested with NERINE for two UKBB phenotypes: (1) high LDL-C vs. low LDL-C, and (2) low HDL-C vs. high HDL-C. For each pathway, the most significant *p* value is highlighted in boldface.

Table S5. Database-wide significant pathways identified by NERINE in the meta-analysis of UKBB and MGBBB cohorts for breast cancer, type 2 diabetes, coronary artery disease, and early-onset myocardial infarction, related to Figure 4 Results for variant categories—LoF, damaging, and damaging missense are shown. Bonferroni correction was applied on the Fisher’s combined test *p* values.

Table S7. Pairwise Jaccard similarity and overlap between NERINE-identified database-wide significant pathways for early-onset myocardial infarction, related to Figure 4 The upper triangle shows the number of overlapping genes for each pair of pathways. The lower triangle shows the corresponding Jaccard indices. Number of genes per pathway is shown in parentheses.

Table S8. NERINE-predicted gene effects across each significant pathway for breast cancer in UKBB and MGBBB, related to Figure 4 Relative gene effects range from −4 and 4. Here, 0 indicates no effect on the trait. Positive values indicate varying degrees of trait-increasing effects, and negative values indicate varying degrees of trait-decreasing effects. For each pathway, gene effects in only the categories where significant network-level *p* values were observed are shown. Average effects are used for visualizing the networks. Relative proportions of cases and controls carrying the qualifying mutations in the genes within each cohort are also shown. Relative proportions of cases with variant alleles for gene *x* are calculated as (*k*_*case*_/*N*_*case*_)/(*k*_*case*_/*N*_*case*_+*k*_*control*_/*N*_*control*_), where *k*_*g*_ denotes the number of individuals carrying qualifying variant alleles in group *g*. Relative proportions in controls are calculated analogously. Information sources: GWAS catalog (https://www.ebi.ac.uk/gwas/); COSMIC (https://cancer.sanger.ac.uk/cosmic); and DGIdb (https://dgidb.org).

Table S9. NERINE-predicted gene effects across the adipogenesis pathway (BIOCARTA VOBESITY PATHWAY) for type 2 diabetes in UKBB and MGBBB, related to Figure 4 Relative gene effects range from −4 and 4. Here, 0 indicates no effect on the trait. Positive values indicate varying degrees of trait-increasing effects, and negative values indicate varying degrees of trait-decreasing effects. Gene effects in only the categories where significant network-level *p* values were observed are shown. Average effects are used for visualizing the networks. Relative proportions of cases and controls carrying the qualifying mutations in the genes within each cohort are also shown. Relative proportions of cases with variant alleles for gene *x* are calculated as (*k*_*case*_/*N*_*case*_)/(*k*_*case*_/*N*_*case*_+*k*_*control*_/*N*_*control*_), where *k*_*g*_ denotes the number of individuals carrying qualifying variant alleles in group *g*. Relative proportions in controls are calculated analogously. Information on whether a gene is a GWAS hit is derived from the GWAS catalog (https://www.ebi.ac.uk/gwas/).

Table S10. NERINE-predicted gene effects across significant and suggestive pathways for coronary artery disease in UKBB and MGBBB, related to Figure 4 Relative gene effects range from −4 and 4. Here, 0 indicates no effect on the trait. Positive values indicate varying degrees of trait-increasing effects, and negative values indicate varying degrees of trait-decreasing effects. Gene effects in only the categories where significant network-level *p* values were observed are shown. Average effects are used for visualizing the networks. Relative proportions of cases and controls carrying the qualifying mutations in the genes within each cohort are also shown. Relative proportions of cases with variant alleles for gene *x* are calculated as (*k*_*case*_/*N*_*case*_)/(*k*_*case*_/*N*_*case*_+*k*_*control*_/*N*_*control*_), where *k*_*g*_ denotes the number of individuals carrying qualifying variant alleles in group *g*. Relative proportions in controls are calculated analogously. Information on whether a gene is a GWAS hit is derived from the GWAS catalog (https://www.ebi.ac.uk/gwas/).

Table S11. NERINE-predicted gene effects across significant pathways for early-onset myocardial infarction in UKBB and MGBBB, related to Figure 4 Relative gene effects range from −4 and 4. Here, 0 indicates no effect on the trait. Positive values indicate varying degrees of trait-increasing effects, and negative values indicate varying degrees of trait-decreasing effects. Gene effects in only the categories where significant network-level *p* values were observed are shown. Average effects are used for visualizing the networks. Relative proportions of cases and controls carrying the qualifying mutations in the genes within each cohort are also shown. Relative proportions of cases with variant alleles for gene *x* are calculated as (*k*_*case*_/*N*_*case*_)/(*k*_*case*_/*N*_*case*_+*k*_*control*_/*N*_*control*_), where *k*_*g*_ denotes the number of individuals carrying qualifying variant alleles in group *g*. Relative proportions in controls are calculated analogously. Information on whether a gene is a GWAS hit is derived from the GWAS catalog (https://www.ebi.ac.uk/gwas/).

Table S13. NERINE identifies screen-wide significant rare LoF variant burden in peptidyl-threonine modification module enriched for GWAS genes in Parkinson’s disease, related to STAR Methods, Figure S18, and Methods S1 NERINE was applied on six GO biological process (BP) modules enriched for PD GWAS genes (STAR Methods). For each module, we competitively tested network topologies built with (1) physical and genetic interactions, (2) co-expression in the substantia nigra region of the mid-brain, and (3) co-essentiality in CNS cell lines. Across UKBB-sporadic and AMP-PD cohorts, we tested rare variants (MAF <0.001) in six categories—(1) LoF, (2) damaging missense, (3) damaging, (4) missense, (5) neutral, and (6) synonymous (STAR Methods). For each type of network topology, *p* values were meta-analyzed using Fisher’s combined test. Bonferroni correction was applied on the combined *p* values.

Table S14. NERINE-predicted gene effects in the peptidyl-threonine modification module enriched for PD GWAS genes across UKBB-sporadic and AMP-PD cohorts, related to STAR Methods, Figure S18, and Methods S1 Relative gene effects range from −4 and 4. Here, 0 indicates no effect on the trait. Positive values indicate varying degrees of trait-increasing effects, and negative values indicate varying degrees of trait-decreasing effects. Gene effects in only the categories where significant network-level *p* values were observed are shown. Average effects are used for visualizing the networks. Relative proportions of cases and controls carrying the qualifying mutations in the genes within each cohort are also shown. Relative proportions of cases with variant alleles for gene *x* are calculated as (*k*_*case*_/*N*_*case*_)/(*k*_*case*_/*N*_*case*_+*k*_*control*_/*N*_*control*_), where *k*_*g*_ denotes the number of individuals carrying qualifying variant alleles in group *g*. Relative proportions in controls are calculated analogously.

Table S16. NERINE identifies screen-wide significant rare damaging and damaging missense variant burden in the regulation of autophagy module enriched for dopaminergic neuron essentiality genes in Parkinson’s disease, related to Figure 6 NERINE was applied on ten GO biological process (BP) modules from the significant hits of genome-wide essentiality screen for DA neurons (STAR Methods). For each module, we competitively tested network topologies built with (1) physical and genetic interactions, (2) co-expression in the substantia nigra of the mid-brain, and (3) co-essentiality in CNS cell lines. Across UKBB-extreme and AMP-PD cohorts, we tested rare variants (MAF < 0.01) in six categories—(1) LoF, (2) damaging missense, (3) damaging, (4) missense, (5) neutral, and (6) synonymous (STAR Methods). For each type of network topology, *p* values were meta-analyzed across both cohorts using the Fisher’s combined test. Bonferroni correction was applied on the combined *p* values.

Table S17. NERINE-predicted gene effects in the regulation of autophagy module enriched for DA neuron essentiality genes in UKBB-extreme and AMP-PD cohorts, related to Figure 6 Relative gene effects range from −4 and 4. Here, 0 indicates no effect on the trait. Positive values indicate varying degrees of trait-increasing effects, and negative values indicate varying degrees of trait-decreasing effects. Gene effects in only the categories where significant network-level *p* values were observed are shown. Average effects are used for visualizing the networks. Relative proportions of cases and controls carrying the qualifying mutations in the genes within each cohort are also shown. Relative proportions of cases with variant alleles for gene *x* are calculated as (*k*_*case*_/*N*_*case*_)/(*k*_*case*_/*N*_*case*_+*k*_*control*_/*N*_*control*_), where *k*_*g*_ denotes the number of individuals carrying qualifying variant alleles in group *g*. Relative proportions in controls are calculated analogously.

Table S18. NERINE identifies screen-wide significant rare damaging missense variant burden in the SNCA-LRRK2-containing vesicle trafficking and protein homeostasis stem of the ɑS proteotoxicity network in Parkinson’s disease, related to Figure 6 For each subnetwork, NERINE was with the TransposeNet network topology. Across UKBB-sporadic and AMP-PD cohorts, we tested rare variants (MAF < 0.001) in six categories—(1) LoF, (2) damaging missense, (3) damaging, (4) missense, (5) neutral, and (6) synonymous (STAR Methods). For each stem, *p* values were meta-analyzed using the Fisher’s combined test. Bonferroni correction was applied on the combined *p* values.

Table S19. NERINE-predicted gene effects across the SNCA-LRRK2-containing vesicle trafficking and protein homeostasis stem of the ɑS proteotoxicity network in UKBB-sporadic and AMP-PD cohorts, related to Figure 6 Relative gene effects range from −4 and 4. Here, 0 indicates no effect on the trait. Positive values indicate varying degrees of trait-increasing effects, and negative values indicate varying degrees of trait-decreasing effects. Gene effects in only the damaging missense category are shown, as rare variants only in this category yielded network-level significance. Average effects are used for visualizing the networks. Relative proportions of cases and controls carrying the qualifying mutations in the genes within each cohort are also shown. Relative proportions of cases with variant alleles for gene *x* are calculated as (*k*_*case*_/*N*_*case*_)/(*k*_*case*_/*N*_*case*_+*k*_*control*_/*N*_*control*_), where *k*_*g*_ denotes the number of individuals carrying qualifying variant alleles in group *g*. Relative proportions in controls are calculated analogously.

Table S21. List of canonical pathways in the pathway database used in the study, related to Figure 4 and STAR Methods

Table S22. List of DepMap (v.2023Q2) cell lines from liver and the central nervous system, related to Figures 3 and 6 and STAR Methods

Table S23. NERINE identifies nominally significant rare variant burden in MAGMA GO modules enriched for GWAS loci in Parkinson’s disease, related to STAR Methods We tested 21 significant, conditionally independent GO biological process (BP) modules identified by the Kim et al., 2024 study^65^ using MAGMA and GO term enrichment. For each module, NERINE was competitively applied with network topologies created from (1) physical and genetic interactions, (2) co-expression in the substantia nigra of the mid-brain, and (3) co-essentiality in CNS cell lines. Across UKBB-sporadic and AMP-PD cohorts, we tested rare variants (MAF < 0.001) in six categories—(1) LoF, (2) damaging missense, (3) damaging, (4) missense, (5) neutral, and (6) synonymous (STAR Methods). For each type of network topology, *p* values were meta-analyzed using Fisher’s combined test. Bonferroni correction was applied on the combined *p* values. Results from functionally important categories—LoF, damaging, and damaging missense—are shown.

Document S2. Article plus supplemental information
