# Supplementary material for: NERINE reveals rare variant associations in gene networks across phenotypes and implicates an SNCA-PRL-LRRK2 subnetwork in Parkinson’s disease
Source: Cell Genom. 2026 Jun 22;6(7):101284. doi: 10.1016/j.xgen.2026.101284 (PMC13347950; doi:10.1016/j.xgen.2026.101284)
Supplement: Document S1. Figures S1–S26, Tables S1, S4, S6, S12, S15, and S20, and Methods S1–S6 [file mmc1.pdf]

Cell Genomics, Volume *6*

Supplemental information

NERINE reveals rare variant associations in gene networks across phenotypes and implicates an *SNCA-PRL-LRRK2* subnetwork in Parkinson's disease

Sumaiya Nazeen, Xinyuan Wang, Autumn R. Morrow, Ronya Strom, Elizabeth Ethier, Dylan Ritter, Alexander B.H. Henderson, Jalwa Afroz, Christopher S. Cassa, Nathan O. Stitziel, Rajat M. Gupta, Kelvin C. Luk, Lorenz Studer, Vikram Khurana, and Shamil R. Sunyaev

Supplementary Figures

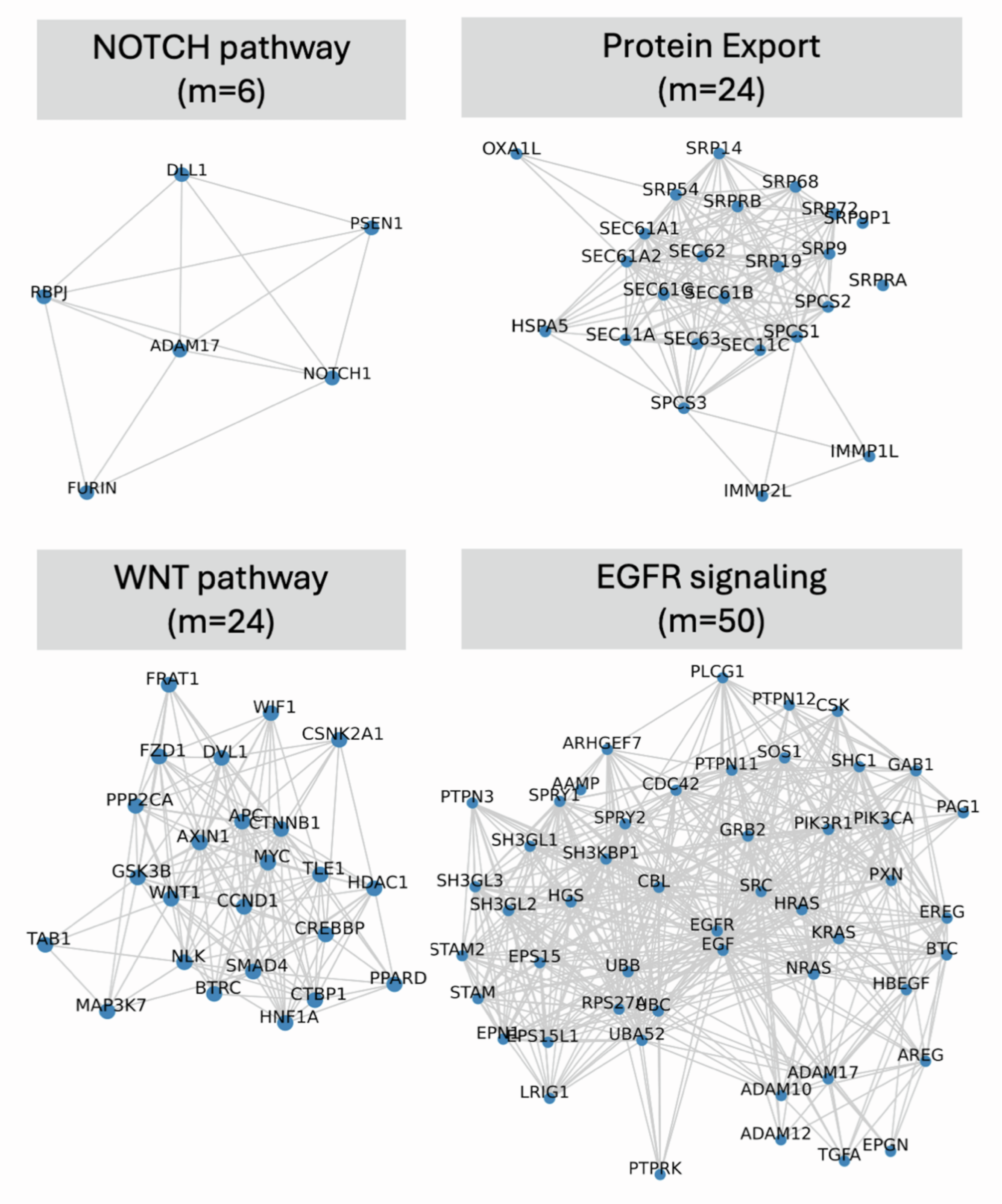

Figure S1. Canonical pathway networks used for testing NERINE's performance in simulations, related to Figure 2 and STAR Methods.

Four well-studied pathways of different sizes: NOTCH pathway (m = 6), WNT pathway (m = 24), protein export (m = 24), and EGFR signaling (m = 50) were used for simulations. Canonical pathway gene lists were extracted from MSigDB (v7.3), and high-confidence physical and genetic interactions from protein-protein interaction (PPI) databases were used as network edges between pathway genes (STAR Methods).

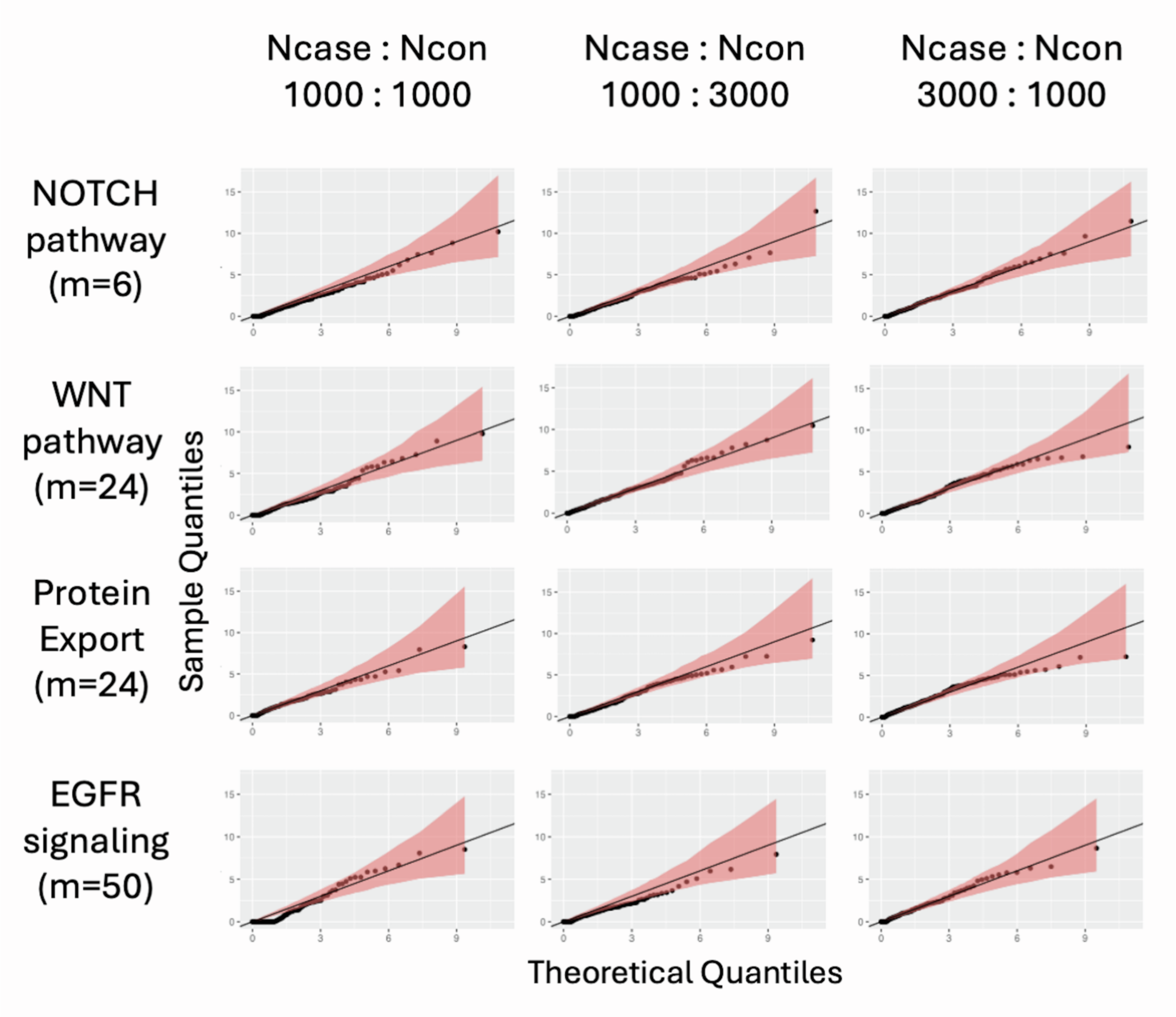

Figure S2. NERINE's performance in simulations at null with canonical pathway networks, related to Figure 1 and STAR Methods.

NERINE's test statistic asymptotically follows the theoretical distribution of a mixture of the delta function (point-mass at zero) and a chi-square distribution with one degree of freedom. Simulations were performed with different network architectures for four canonical pathways of different sizes: NOTCH pathway (m = 6), WNT pathway (m = 24), protein export (m = 24), and EGFR signaling (m = 50). Pathway gene lists were extracted from MSigDB (v7.3), and high-confidence physical and genetic interactions from protein-protein interaction (PPI) databases were used as network edges between pathway genes (STAR Methods). The allele counts in cases and controls were generated from independent binomial distributions. Simulations were performed in cohorts with different case-control skews (STAR Methods). For each scenario, 1,000 iterations were performed to create the QQ plots. Confidence bands in the QQ plots represent 95% bootstrap confidence intervals around NERINE's test statistic.

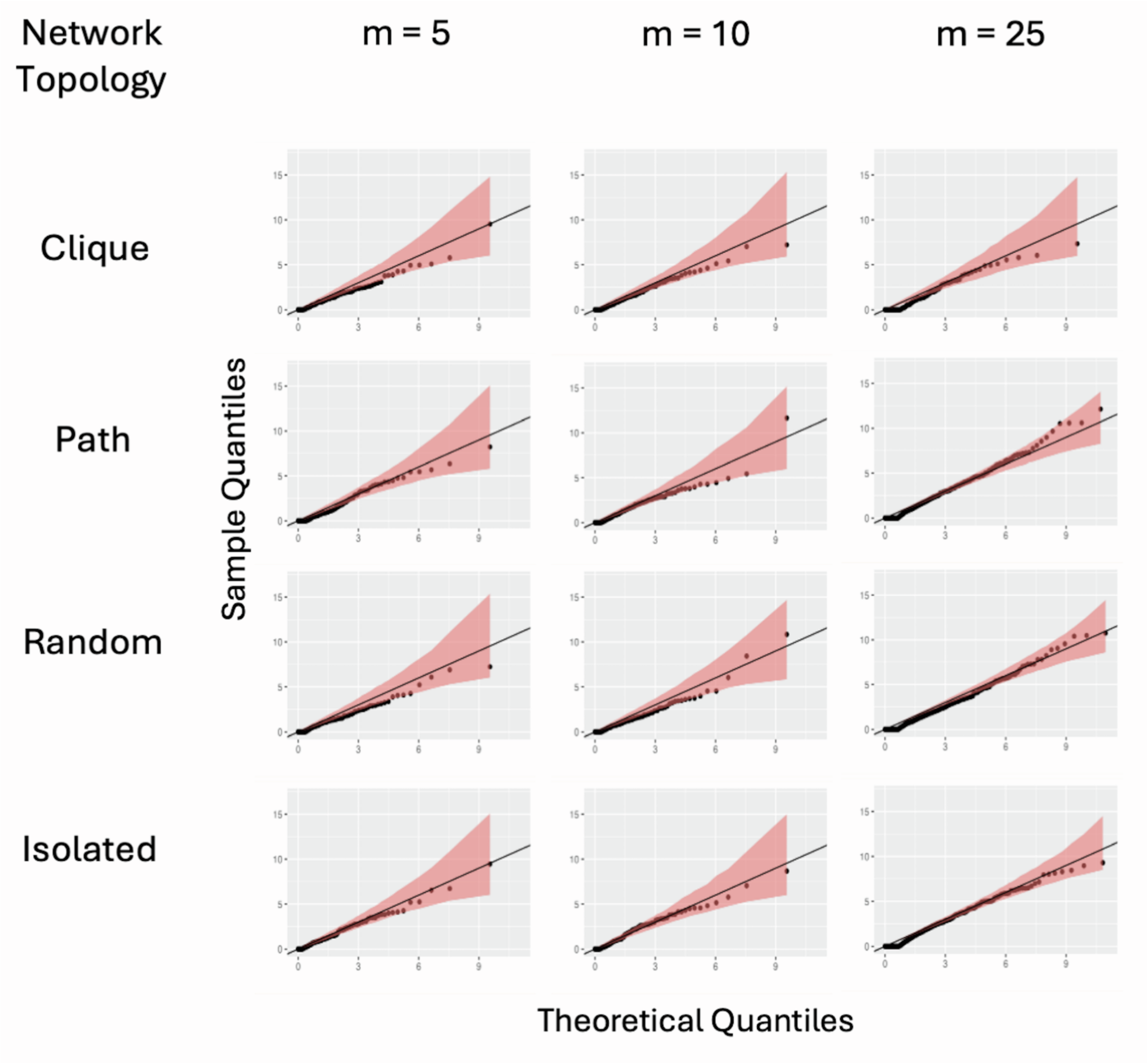

Figure S3. NERINE's performance in simulations at null with artificial network topologies, related to Figure 1 and STAR Methods.

Simulations were performed using networks of different sizes (i.e., 5, 10, and 25 genes) and different topological architectures (i.e., clique, path, random, and isolated nodes) for equal-sized case- and control-groups (STAR Methods). For each scenario, 1,000 iterations were performed to generate the QQ plots. The allele counts in cases and controls were generated from independent binomial distributions (STAR Methods). Confidence bands in the QQ plots represent 95% bootstrap confidence intervals around NERINE's test statistic. In each scenario, NERINE's test statistic asymptotically follows the theoretical distribution of a mixture of the delta function (point-mass at zero) and a chi-square distribution with one degree of freedom.

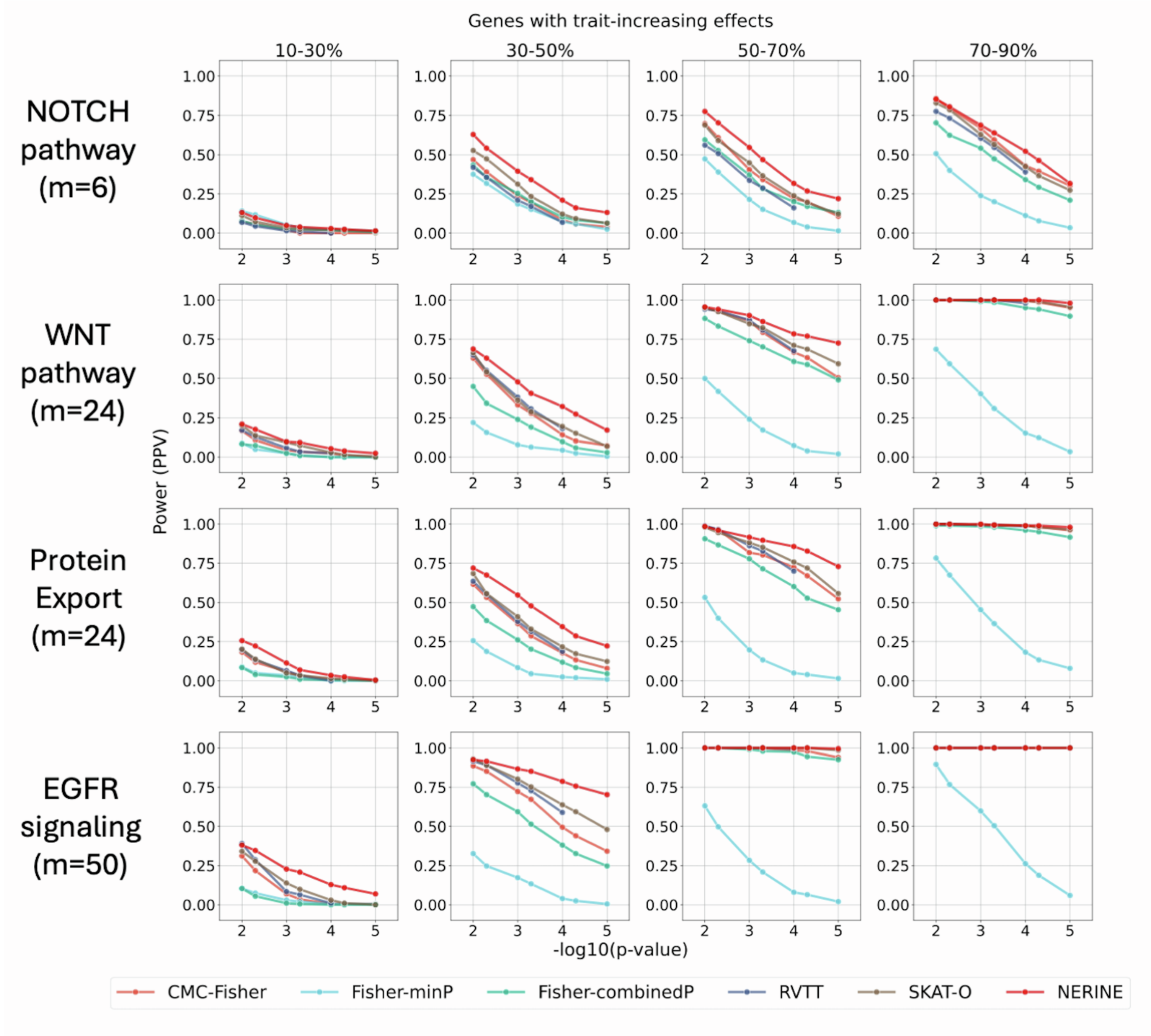

Figure S4. NERINE outperforms existing rare variant association tests in power simulations when relevant genes have only trait-increasing effects, related to Figure 2 and STAR Methods.

Power was evaluated using a simulated binary trait (2,000 cases and 2,000 controls) across four canonical pathways (NOTCH pathway, WNT pathway, protein export, and EGFR signaling) with non-zero network effect ($\theta = 0.2$). Noise was varied by the proportion of genes with trait-increasing effects (10–90%), spanning highly noisy (only 10–30% genes with effect) to highly informative (70–90% genes with effect) networks. For each noise profile, 250 iterations were performed per network, and power was measured as the positive predictive value (PPV) at different significance cutoffs ($1 \times 10^{-2}, 5 \times 10^{-3}, 1 \times 10^{-3}, 5 \times 10^{-4}, 1 \times 10^{-4}, 5 \times 10^{-5}$, and 1×10^{-5}) (STAR Methods). NERINE consistently outperformed existing rare-variant association tests, with the largest gains in noisy settings. All tests were two-sided.

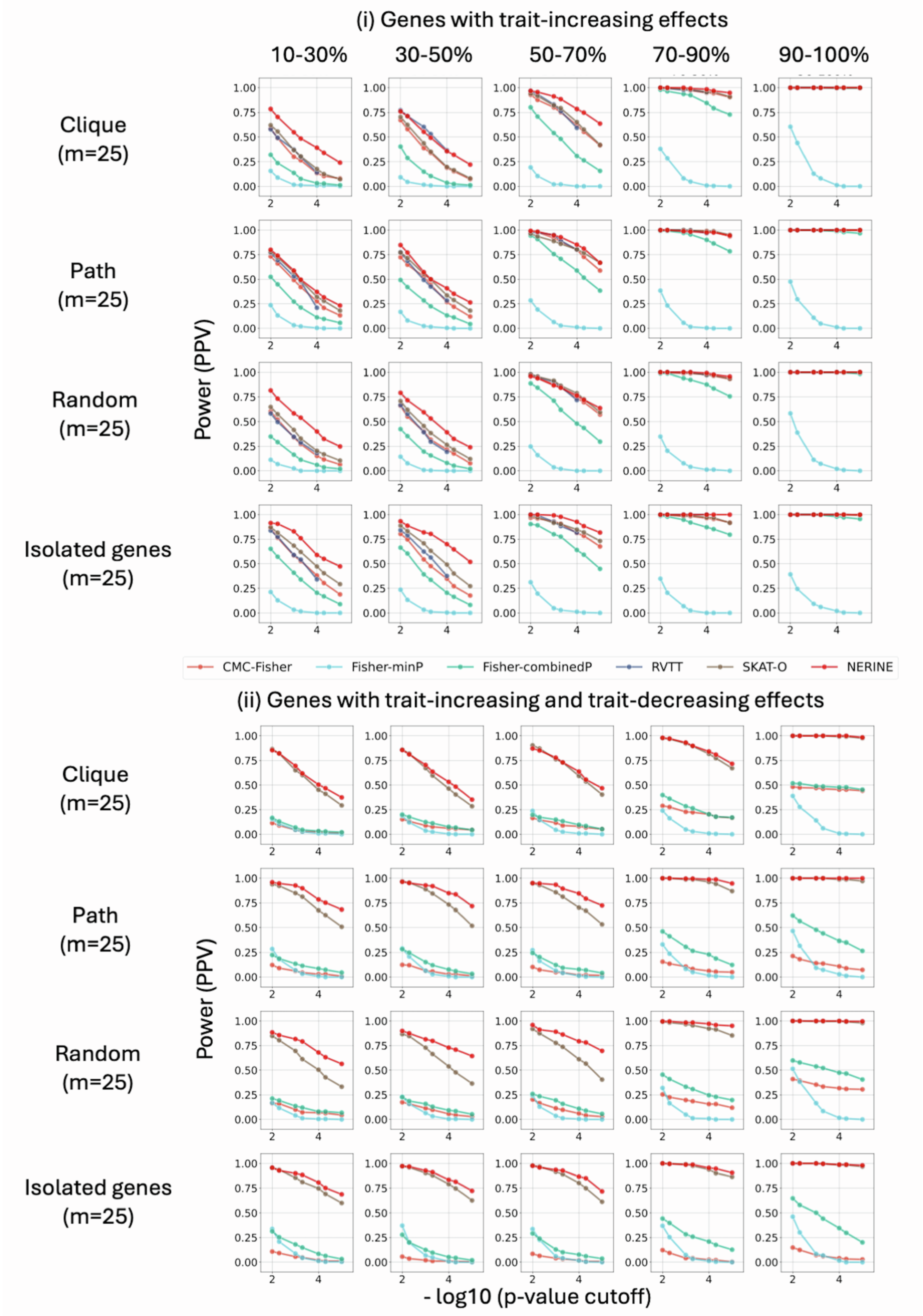

(i) Genes with trait-increasing effects
10-30%
30-50%
50-70%
70-90%
90-100%
Clique (m=25)
Path (m=25)
Random (m=25)
Isolated genes (m=25)
Power (PPV)
CMC-Fisher
Fisher-minP
Fisher-combinedP
RVTT
SKAT-O
NERINE
(ii) Genes with trait-increasing and trait-decreasing effects
Clique (m=25)
Path (m=25)
Random (m=25)
Isolated genes (m=25)
Power (PPV)
- log10 (p-value cutoff)

Figure S5. NERINE outperforms existing rare variant association tests in power simulations with artificial network topologies, related to Figure 2 and STAR Methods.

Power was evaluated under the alternative hypothesis ($\theta = 0.5$) using a simulated binary trait (1,000 cases and 1,000 controls) across networks with different topological architectures (i.e., clique, path, random, and isolated nodes) in two scenarios: (i) genes in the network having only trait-increasing effects, and (ii) genes in the network having both trait-increasing and trait-decreasing effects. Noise was varied by the proportion of genes with trait-increasing effects (10–100%), spanning highly noisy (only 10–30% genes with effect) to highly informative (90–100% genes with effect) networks. For each noise profile, 250 iterations were performed per network, and power was measured as the positive predictive value (PPV) at different significance cutoffs ($1 \times 10^{-2}, 5 \times 10^{-3}, 1 \times 10^{-3}, 5 \times 10^{-4}, 1 \times 10^{-4}, 5 \times 10^{-5}$, and 1×10^{-5}) (STAR Methods). NERINE consistently outperformed existing rare-variant association tests, with the largest gains in noisy settings. NERINE's performance is comparable to SKAT-O when networks contain very little noise. Note that, RVTT *p*-values were calculated from 10,000 permutations. Hence, we don't report RVTT's power for the cutoffs below 1×10^{-4}. Also, RVTT is a test for monotonic trends in rare variant occurrences within a pathway. Hence, it was excluded from the comparison in scenario (ii). All tests were two-sided.

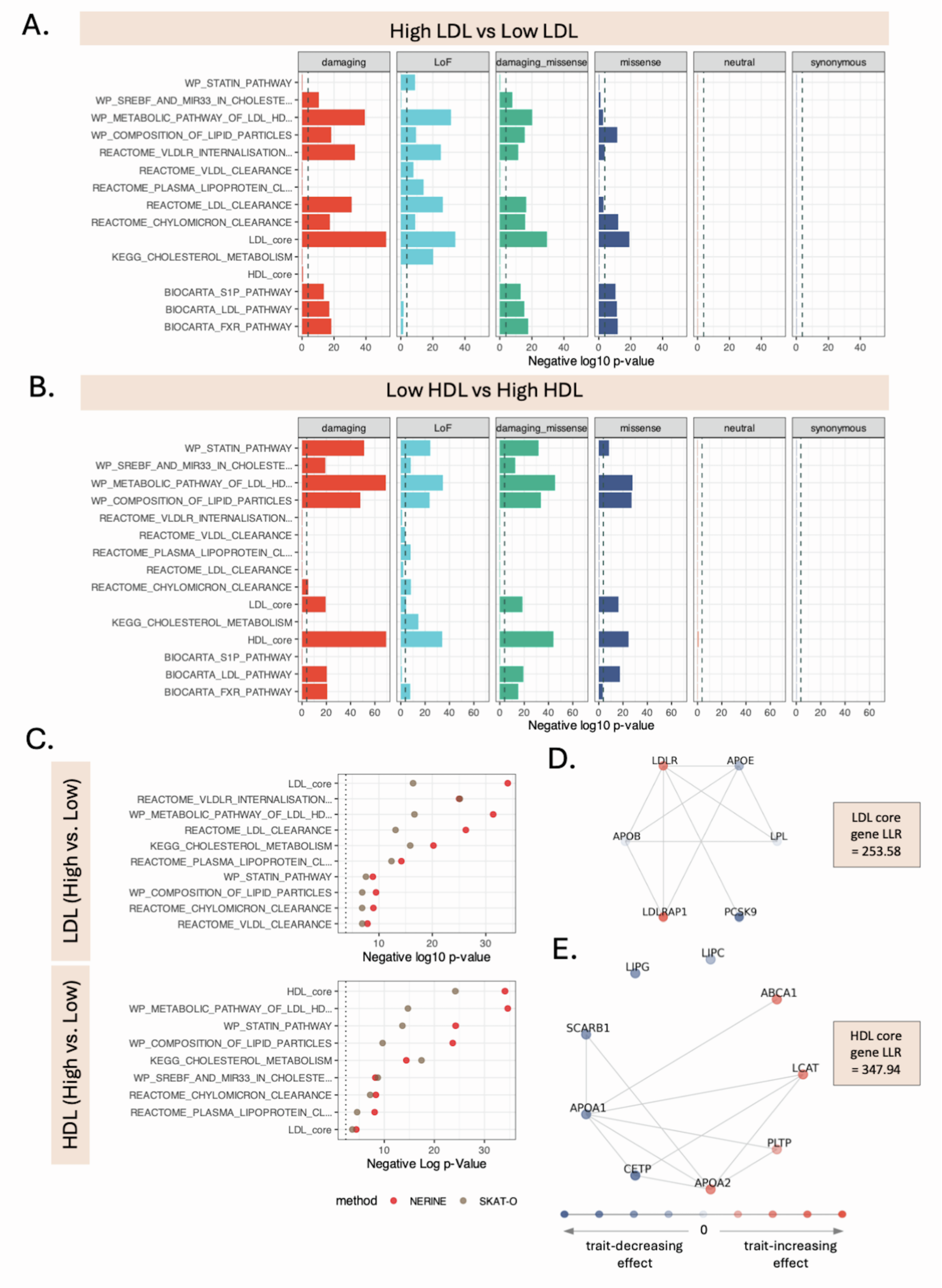

A.
High LDL vs Low LDL
B.
Low HDL vs High HDL
damaging
LoF
damaging_missense
missense
neutral
synonymous
WP_STATIN_PATHWAY
WP_SREBF_AND_MIR33_IN_CHOLESTE...
WP_METABOLIC_PATHWAY_OF_LDL_HD...
WP_COMPOSITION_OF_LIPID_PARTICLES
REACTOME_VLDLR_INTERNALISATION...
REACTOME_VLDL_CLEARANCE
REACTOME_PLASMA_LIPOPROTEIN_CL...
REACTOME_LDL_CLEARANCE
REACTOME_CHYLOMICRON_CLEARANCE
LDL_core
KEGG_CHOLESTEROL_METABOLISM
HDL_core
BIOCARTA_S1P_PATHWAY
BIOCARTA_LDL_PATHWAY
BIOCARTA_FXR_PATHWAY
Negative log10 p-value
C.
LDL (High vs. Low)
HDL (High vs. Low)
Negative log10 p-value
Negative Log p-Value
method
NERINE
SKAT-O
D.
LDLR
APOE
APOB
LPL
LDLRAP1
PCSK9
LDL core gene LLR = 253.58
E.
LIPG
LIPC
ABCA1
SCARB1
LCAT
APOA1
PLTP
CETP
APOA2
HDL core gene LLR = 347.94
0
trait-decreasing effect
trait-increasing effect

Figure S6. Performance of NERINE on binarized LDL-C and HDL-C phenotypes in the UK Biobank, related to Results and STAR Methods.

A. While comparing individuals with high LDL-C with the ones with low LDL-C in UKBB, NERINE identifies significant cumulative effect of rare (MAF < 0.001) variants in LoF (i.e., frameshifts, insertions, deletions, and splice variants), damaging missense (i.e., missenses predicted to be damaging by in-silico tools), damaging (i.e., damaging missense and LoF), and missense categories in key lipid-related pathways. No significant burden of neutral missense and synonymous variants was observed. The tests were performed across our canonical pathway database of 306 pathways. Pathway gene lists were extracted from MSigDB (v7.3), and high-confidence physical and genetic interactions from protein-protein interaction (PPI) databases were used as network edges between pathway genes (STAR Methods). The dashed grey line represents the Bonferroni-corrected p-value threshold of 0.05. The core module of LDL genes, which contains *LDLR* and *PCSK9*, was identified as the most significant hit, which serves as a positive control.

B. While comparing individuals with low HDL-C with the ones with high HDL-C in UKBB, NERINE identifies a significant cumulative effect of rare (MAF < 0.001) variants in LoF, damaging missense, damaging, and missense categories in key lipid-related pathways. Notably, the module of core HDL-related genes containing *ABCA1*, *CETP*, *LIPC*, and *LIPG* was the most significant hit, serving as a positive control. No significant burden of neutral missense and synonymous variants was observed. The tests were performed across the same canonical pathway database. The dashed grey line represents the Bonferroni-corrected p-value threshold of 0.05.

C. Comparison of SKAT-O p-values against NERINE p-values for rare LoF variant burden across the significant pathways in the LDL-C (high vs low) and HDL-C (low vs high) phenotypes. For most of the pathways in both phenotypes, NERINE provides a lower p-value than SKAT-O. SKAT-O was applied at the pathway level, aggregating the allele counts from member genes.

D. NERINE's estimates of gene effects in the most significant pathway (LDL core module) with rare damaging variant burden in the LDL-C (high vs low) phenotype. *PCSK9* and *APOB* show trait-decreasing effects, and *LDLR* shows a trait-increasing effect on LDL-C (high vs low) phenotype.

E. NERINE's estimates of gene effects in the most significant pathway (HDL core module) with rare damaging variant burden in the HDL-C (low vs high) phenotype. *ABCA1* and *LCAT* show trait-increasing effects, while *LIPC* and *LIPG* show trait-decreasing effects for the HDL-C (low vs high) phenotype.

In **D** and **E**, trait-increasing effects are represented by shades of orange, and trait-decreasing effects are represented by shades of purple, as shown on the scale. A darker color represents a more pronounced effect. Findings agree with known lipid biology. Note that NERINE's predicted gene effects represent the "most likely scenario" with the observed allele counts per gene and the gene-gene network topology under the estimated network effect. NERINE does not provide p-values on per-gene predictions.

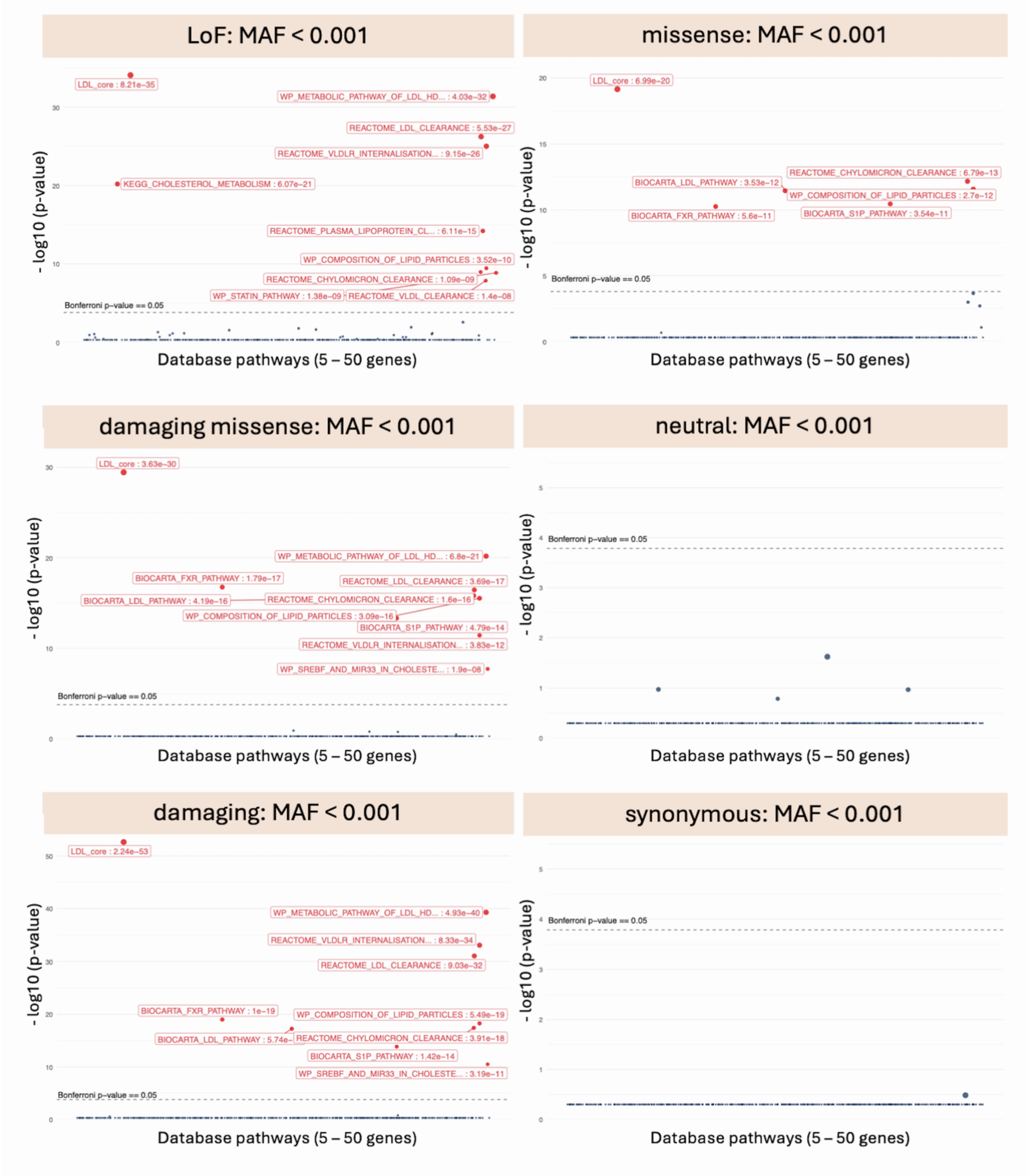

Figure S7. Database pathway gene networks with significant rare variant burden identified by NERINE for the LDL-C phenotype in UKBB, related to Results and STAR Methods.

Pathway Manhattan plots showing NERINE's results in six functional categories of variants: LoF (i.e., frameshifts, insertions, deletions, and splice variants), damaging missense (i.e., missenses predicted to be damaging by in-silico tools), damaging (i.e., LoF and damaging missenses), missense, neutral (i.e.,

missenses predicted to be benign by in-silico tools), and synonymous. Applying NERINE across a database of 306 canonical pathways to compare individuals with high LDL cholesterol with the ones with low LDL cholesterol in UKBB, we identified a significant cumulative effect of rare (MAF < 0.001) variants in LoF, damaging missense, damaging, and missense categories in key lipid-related pathways, such as the core module of LDL-related genes. No significant burden of neutral missense or synonymous variants was observed. The horizontal dashed line represents the Bonferroni-corrected p-value cutoff of 0.05.

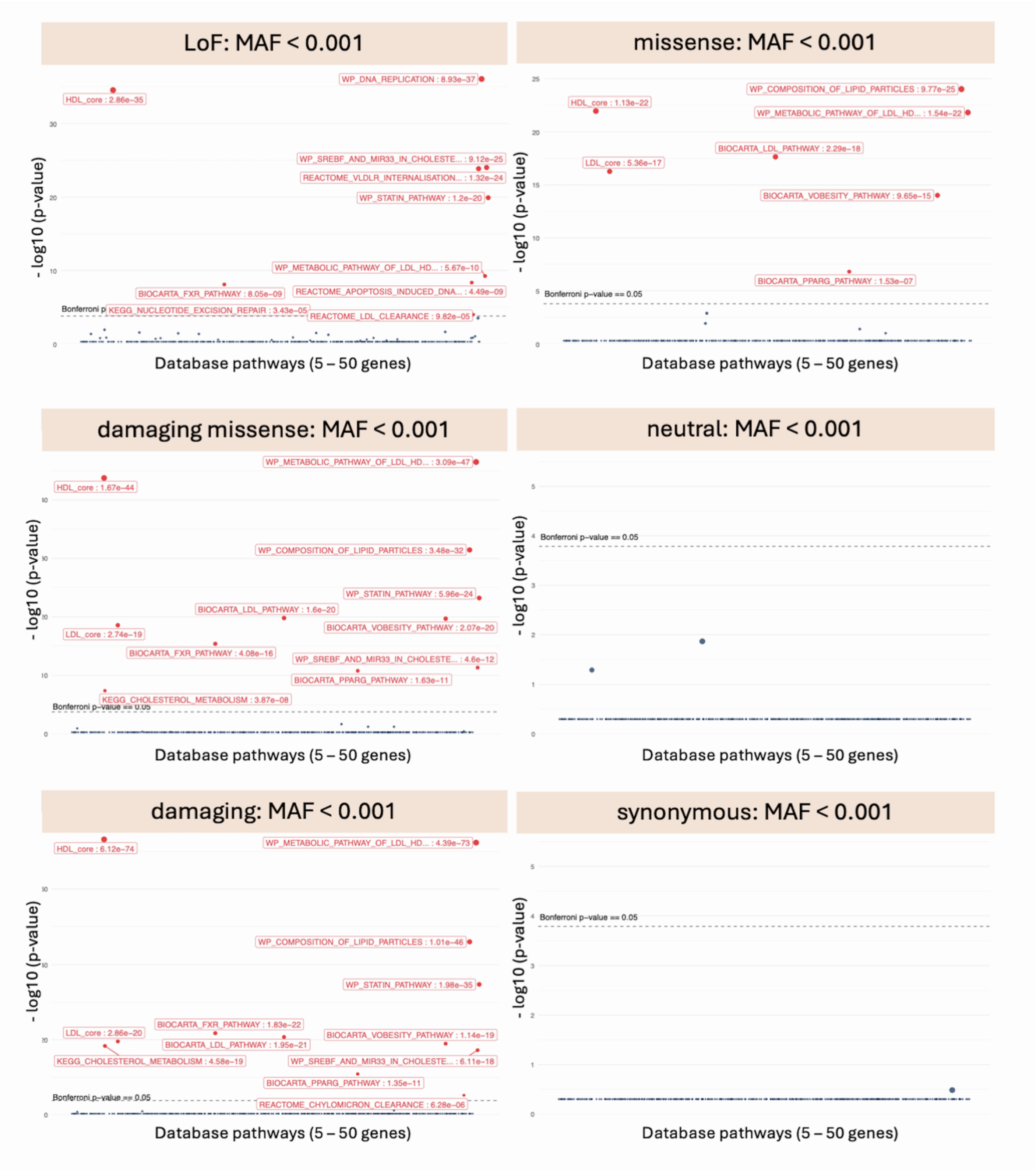

Figure S8. Database pathway gene networks with significant rare variant burden identified by NERINE for the HDL-C phenotype in UKBB, related to Results and STAR Methods.

Pathway Manhattan plots showing NERINE's results in six functional categories of variants: LoF (i.e., frameshifts, insertions, deletions, and splice variants), damaging missense (i.e., missenses predicted to be damaging by in-silico tools), damaging (i.e., LoF and damaging missenses), missense, neutral (i.e., missenses predicted to be benign by in-silico tools), and synonymous. Applying NERINE across a

database of 306 canonical pathways to compare individuals with low HDL cholesterol with the ones with high HDL cholesterol in UKBB, we identified a significant cumulative effect of rare (MAF < 0.001) variants in LoF, damaging missense, damaging, and missense categories in key lipid-related pathways, such as the core module of HDL-related genes. No significant burden of neutral missense or synonymous variants was observed. The horizontal dashed line represents the Bonferroni-corrected p-value cutoff of 0.05.

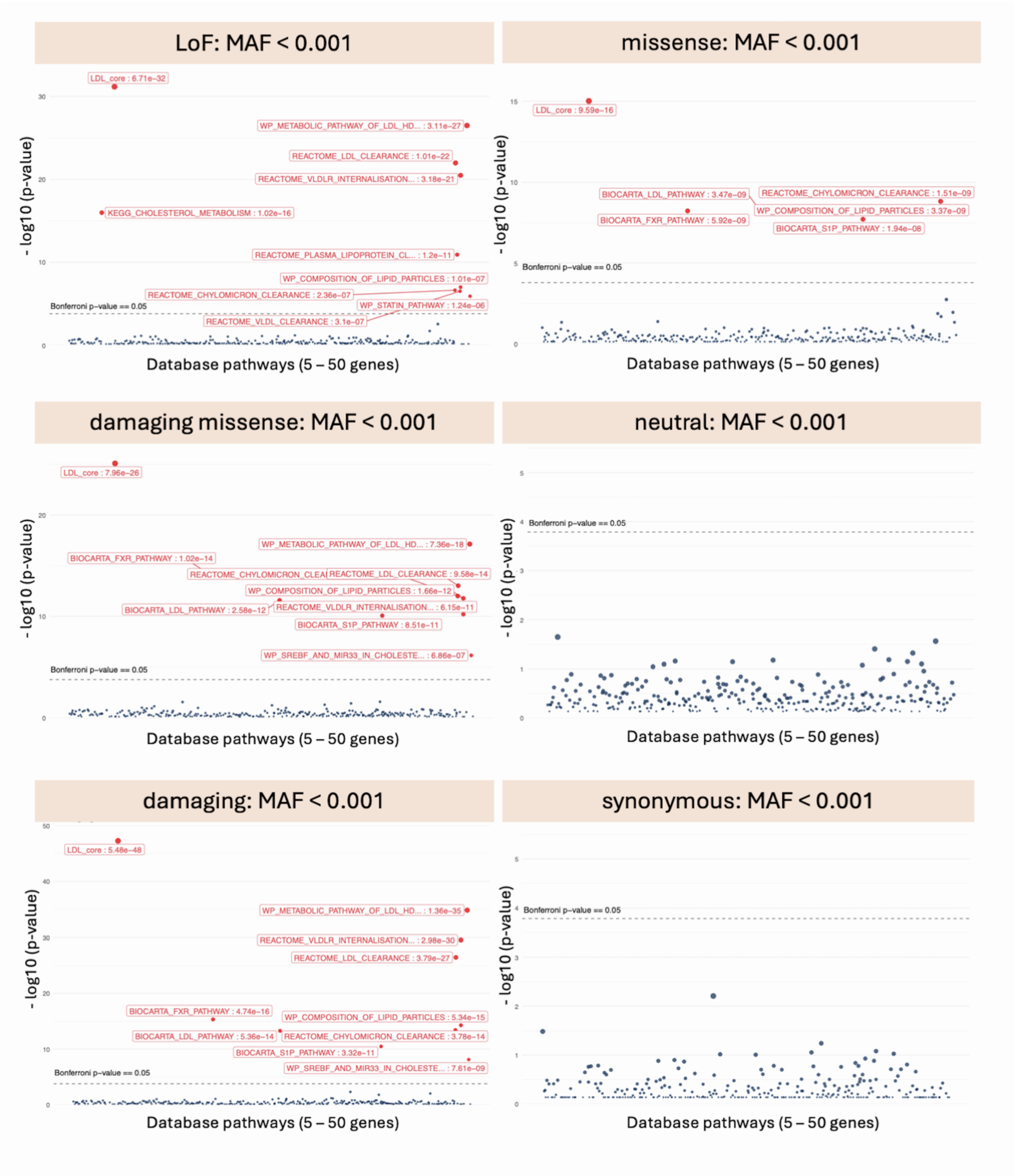

Figure S9. Stratified rare variant burden analysis with NERINE using database pathway modules across multiple ancestries in UKBB for the binarized LDL-C phenotype, related to Results and STAR Methods.

Pathway Manhattan plots showing Fisher's combined p-values across different ancestries in six functional categories of variants: LoF (i.e., frameshifts, insertions, deletions, and splice variants),

damaging missense (i.e., missenses predicted to be damaging by in-silico tools), damaging (i.e., LoF and damaging missenses), missense, neutral (i.e., missenses predicted to be benign by in-silico tools), and synonymous. Applying NERINE across a database of 306 canonical pathways to compare individuals with high LDL cholesterol with the ones with low LDL cholesterol from five major ancestry groups: EUR, AFR, AMR, SAS, and EAS in UKBB, we identified a significant cumulative effect of rare (MAF < 0.001) variants in LoF, damaging missense, damaging, and missense categories in key lipid-related pathways, such as the core module of LDL-related genes. No significant burden of neutral missense or synonymous variants was observed. The horizontal dashed line represents the Bonferroni-corrected combined p-value cutoff of 0.05.

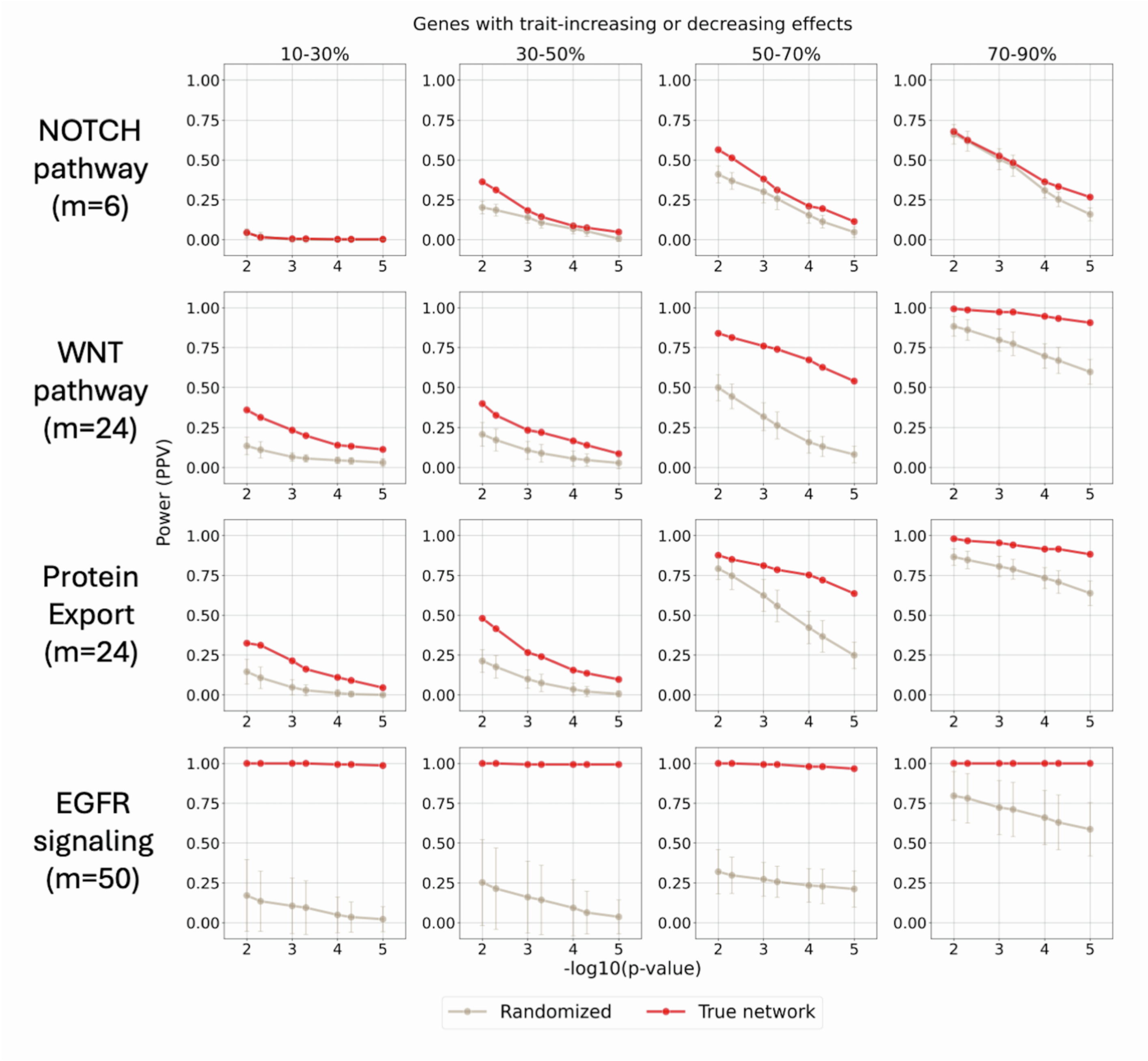

Figure S10. NERINE utilizes the true network topology to achieve greater statistical power in simulations with canonical pathways, related to STAR Methods.

Simulations were performed with different network architectures for four canonical pathways of different sizes: NOTCH pathway (m = 6), WNT pathway (m = 24), protein export (m = 24), and EGFR signaling (m = 50). Pathway gene lists were extracted from MSigDB (v7.3), and high-confidence physical and genetic interactions from protein-protein interaction (PPI) databases were used as true network edges between pathway genes (STAR Methods). For each network gene set, 100 random networks were generated by randomly assigning edges between the genes. The allele counts in cases and controls were generated from independent binomial distributions. The empirical power of the methods was measured for a simulated binary trait in a cohort of 2,000 cases and 2,000 controls, with different proportions of genes in the network having both trait-increasing and trait-decreasing effects simulated with a network effect of $\theta = 0.1$. From left to right, the power plots show networks with different noise profiles,

mimicking situations from having a very noisy network to a highly relevant one. For each noise profile, 250 iterations were performed per network, and power was measured as the positive predictive value (PPV) at different significance cutoffs ($1 \times 10^{-2}, 5 \times 10^{-3}, 1 \times 10^{-3}, 5 \times 10^{-4}, 1 \times 10^{-4}, 5 \times 10^{-5}$, and 1×10^{-5}) (STAR Methods). Error bars represent the standard deviation from the mean PPV across different iterations.

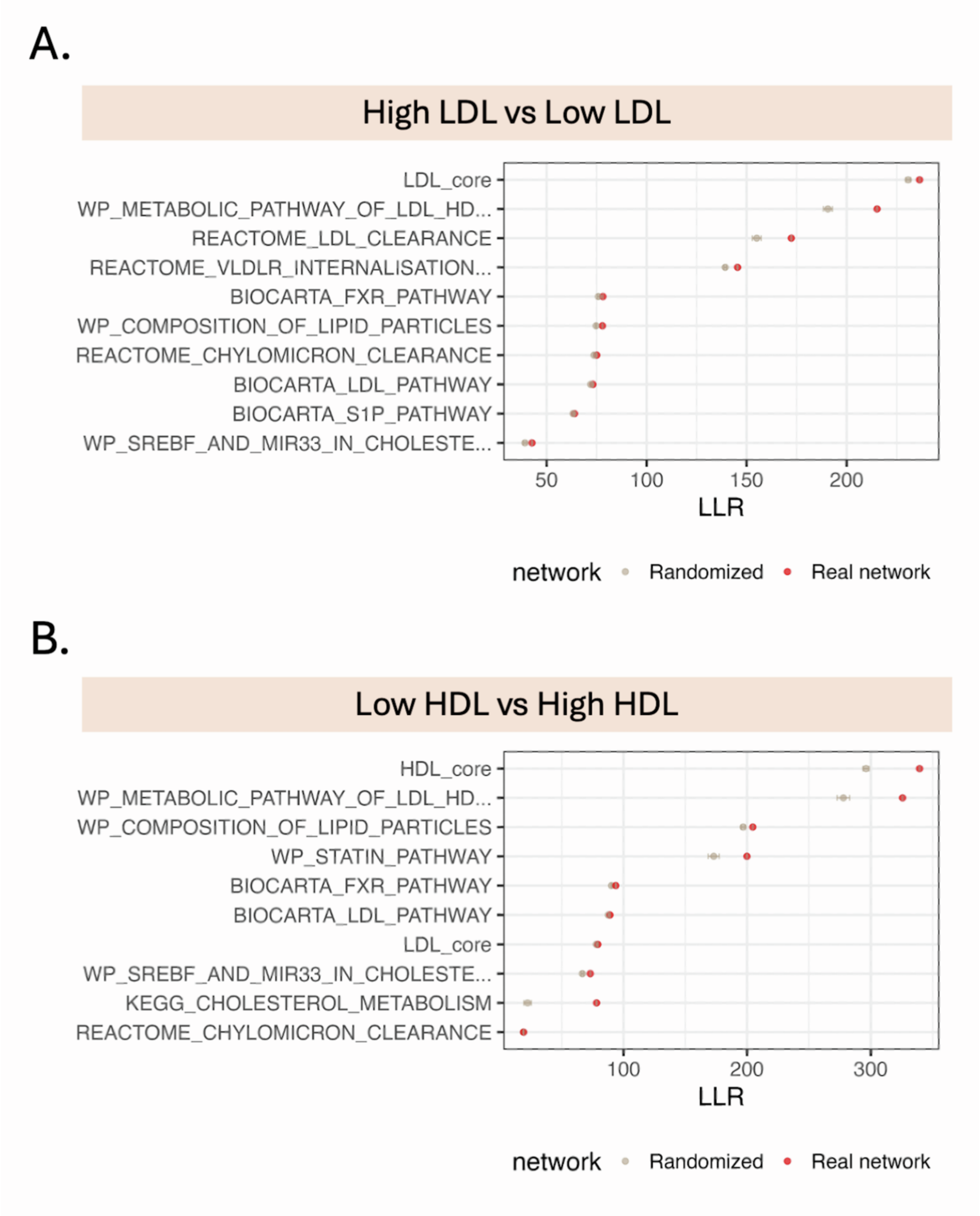

Figure S11. NERINE's performance on real vs. randomized network topologies on binarized LDL-C and HDL-C phenotypes in the UK biobank, related to STAR Methods.

We tested real vs randomized networks in two comparisons in the UKBB cohort: **A)** high LDL-C vs low LDL-C individuals, and **B)** low HDL-C vs high HDL-C individuals. We focused on the database-wide significant pathways in each case and extracted database edges for the member genes to form "real" network topologies (STAR Methods). For each pathway, 100 randomized networks were created by introducing random edges among the member genes. NERINE was applied on both real and randomized network topologies for these phenotypes. For each network in both phenotypes, NERINE achieves a higher log-likelihood ratio (LLR) and lower p-value with real topology than with random edges. Here, the error bars indicate standard error around LLR over 100 random networks.

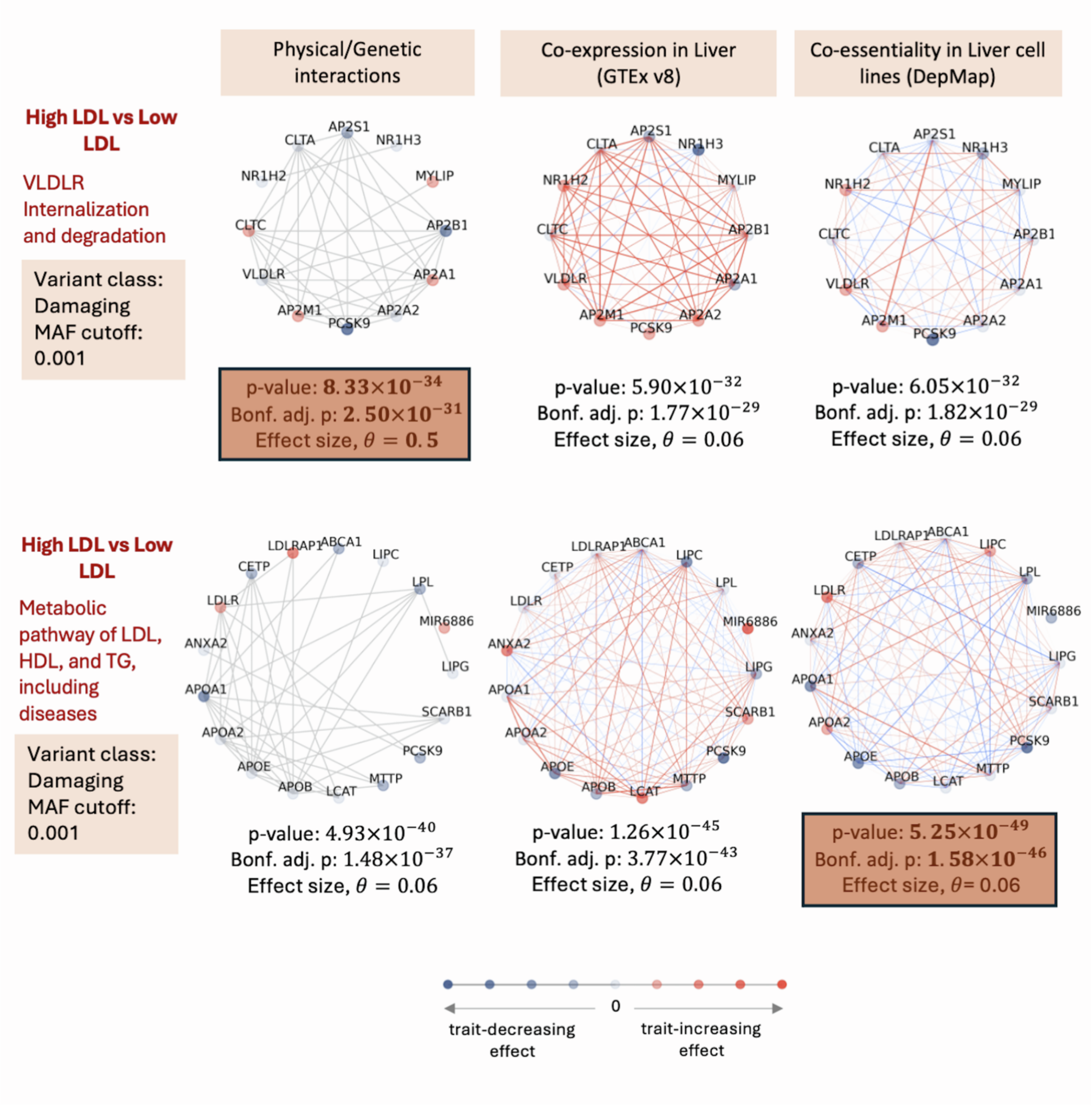

Figure S12. Example scenarios where NERINE selects physical and genetic interactions and co-essentiality relationships as the most informative source of topology, related to Figure 3A.

To describe the edge relationship of selected genes, we used three data sources: high-confidence physical or genetic interactions from protein-protein interaction (PPI) databases, co-expression in liver tissue from GTEx (v8), coessentiality from DepMap (v2023Q2). The top row shows the enrichment of rare damaging variants in the *VLDLR Internalization and degradation* pathway in the binarized LDL-C (high vs low) phenotype in UKBB. In this case, NERINE achieved the most significant p-value and the highest effect size with a network defined by PPI edges. The bottom row shows the enrichment of rare damaging variants in the *metabolic pathway of LDL, HDL, and TG, including diseases* pathway in the binarized LDL-C (high vs low) phenotype in UKBB. The co-essentiality of genes across liver cell lines best describes the relationship of these genes, enabling NERINE to achieve the most significant p-value.

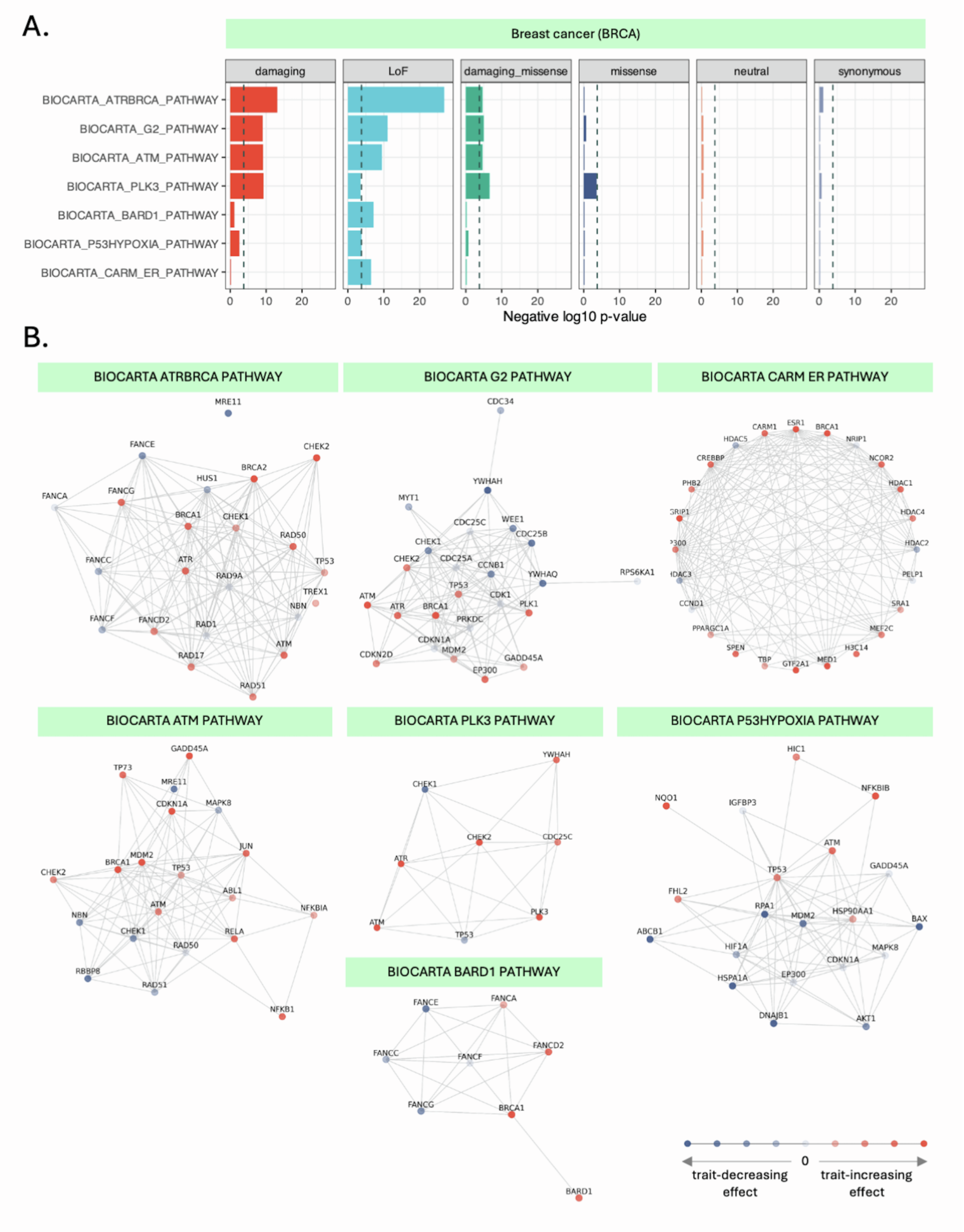

Figure S13. NERINE identifies significant rare variant burden in seven pathway gene modules in breast cancer (BRCA) in the UK and MGB biobanks, related to Figure 4.

A. Bonferroni-significant findings for BRCA across our pathway database of 306 pathways in six functional categories of rare variants—(i) LoF, (ii) damaging missense, (iii) damaging, (iv) missense, (v) neutral missense, and (vi) synonymous (STAR Methods). No inflation was observed in the neutral missense or synonymous variant categories. For each variant category, pathways were tested individually in UKBB and MGBBB cohorts, and p-values were meta-analyzed using Fisher's combined test. Negative log-transformed Fisher's combined p-values for significant pathways are shown. The dashed grey line represents the Bonferroni threshold of 0.05.

B. Network topologies for the pathways with significant rare LoF variant-burden in BRCA. Node color represents NERINE-predicted gene effect, averaged across cohorts (orange: trait-increasing; blue: trait-decreasing; intensity reflects magnitude).

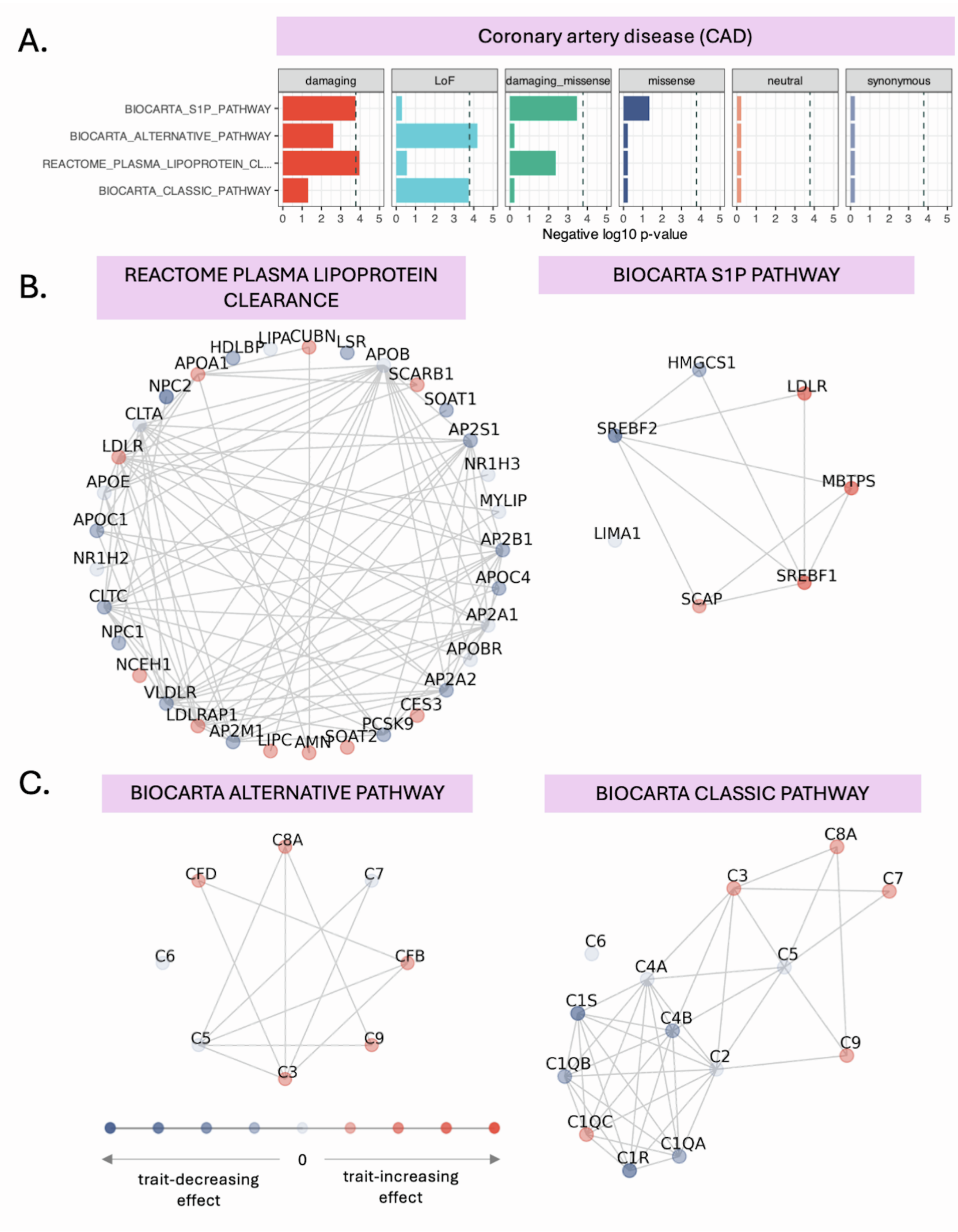

Figure S14. NERINE identifies significant rare variant burden in four pathway gene modules in coronary artery disease (CAD) in the UK and MGB biobanks, related to Figure 4.

A. Bonferroni-significant findings for CAD across our pathway database of 306 pathways in six functional categories of rare variants—(i) LoF, (ii) damaging missense, (iii) damaging, (iv) missense, (v) neutral missense, and (vi) synonymous (STAR Methods). No inflation was observed in neutral missense or synonymous variant categories. Pathways were tested individually in UKBB and MGBBB cohorts for each variant category, and p-values were meta-analyzed using Fisher's combined test. Negative log-transformed Fisher's combined p-values for significant pathways are shown. The dashed grey line represents the Bonferroni threshold of 0.05.

B. Network topologies for the pathways with significant rare damaging variant-burden in CAD with NERINE-predicted gene-level effects. Here, damaging variants refer to LoF and predicted damaging missense variants.

C. Network topologies for the pathways with significant rare LoF variant burden in CAD with NERINE-predicted gene-level effects.

NERINE-predicted gene effects are averaged across cohorts for both **B** and **C**. Node color represents the direction of effect (orange: trait-increasing; blue: trait-decreasing; intensity reflects magnitude).

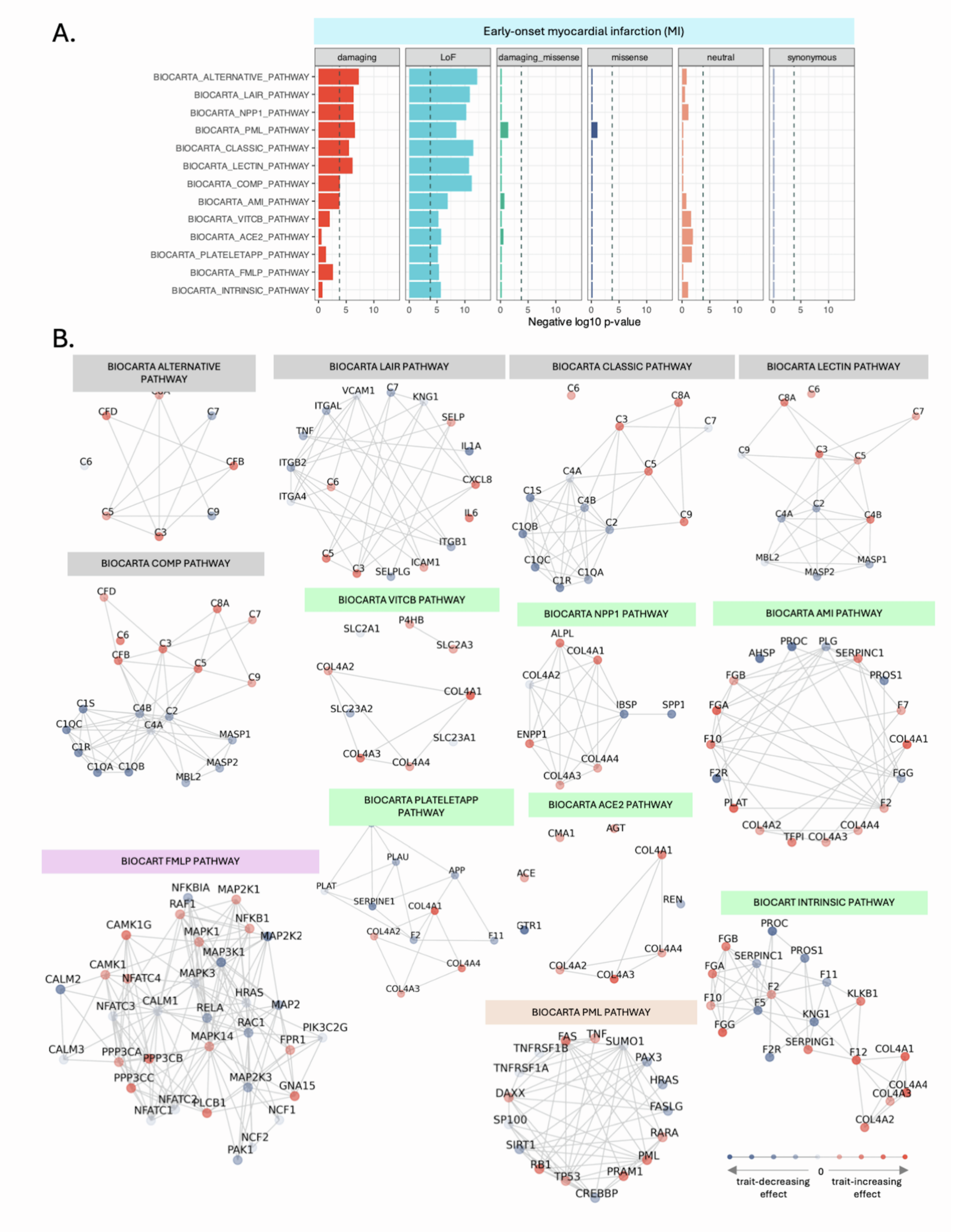
A.
Early-onset myocardial infarction (MI)
damaging
LoF
damaging_missense
missense
neutral
synonymous
BIOCARTA_ALTERNATIVE_PATHWAY
BIOCARTA_LAIR_PATHWAY
BIOCARTA_NPP1_PATHWAY
BIOCARTA_PML_PATHWAY
BIOCARTA_CLASSIC_PATHWAY
BIOCARTA_LECTIN_PATHWAY
BIOCARTA_COMP_PATHWAY
BIOCARTA_AMI_PATHWAY
BIOCARTA_VITCB_PATHWAY
BIOCARTA_ACE2_PATHWAY
BIOCARTA_PLATELETAPP_PATHWAY
BIOCARTA_FMLP_PATHWAY
BIOCARTA_INTRINSIC_PATHWAY
0
5
10
Negative log10 p-value
B.
BIOCARTA ALTERNATIVE PATHWAY
CFD
C7
C6
CFB
C5
C9
C3
BIOCARTA LAIR PATHWAY
VCAM1
C7
KNG1
ITGAL
SELP
TNF
IL1A
ITGB2
CXCL8
C6
ITGA4
IL6
ITGB1
C5
C3
SELPLG
ICAM1
BIOCARTA CLASSIC PATHWAY
C6
C8A
C3
C7
C4A
C5
C1S
C4B
C1QB
C2
C9
C1QC
C1QA
C1R
BIOCARTA LECTIN PATHWAY
C6
C8A
C7
C9
C3
C5
C4A
C2
C4B
MBL2
MASP2
MASP1
BIOCARTA COMP PATHWAY
CFD
C8A
C7
C6
C3
CFB
C5
C9
C1S
C4B
C2
C1QC
C4A
MASP1
C1R
C1QA
C1QB
MASP2
MBL2
BIOCARTA VITCB PATHWAY
P4HB
SLC2A1
SLC2A3
COL4A2
COL4A1
SLC23A2
SLC23A1
COL4A3
COL4A4
BIOCARTA NPP1 PATHWAY
ALPL
COL4A1
COL4A2
IBSP
SPP1
ENPP1
COL4A4
COL4A3
BIOCARTA AMI PATHWAY
PROC
PLG
AHSP
SERPINC1
FGB
PROS1
FGA
F7
F10
COL4A1
F2R
FGG
PLAT
F2
COL4A2
COL4A4
TFPI
COL4A3
BIOCARTA PLATELETAPP PATHWAY
PLAU
APP
PLAT
SERPINE1
COL4A1
COL4A2
F2
F11
COL4A4
COL4A3
BIOCARTA ACE2 PATHWAY
CMA1
AGT
COL4A1
ACE
REN
AGTR1
COL4A4
COL4A2
COL4A3
BIOCART FMLP PATHWAY
NFKBIA
MAP2K1
RAF1
NFKB1
CAMK1G
MAPK1
MAP2K2
MAP3K1
CAMK1
CALM2
NFATC4
MAPK3
NFATC3
CALM1
HRAS
RELA
MAP2
RAC1
PIK3C2G
MAPK14
FPR1
CALM3
PPP3CA
PPP3CB
PPP3CC
MAP2K3
GNA15
NFATC2
PLCB1
NFATC1
NCF1
NCF2
PAK1
BIOCART INTRINSIC PATHWAY
PROC
FGB
SERPINC1
PROS1
FGA
F11
F2
F10
F5
KLKB1
KNG1
FGG
SERPING1
F2R
F12
COL4A1
COL4A4
COL4A3
COL4A2
BIOCARTA PML PATHWAY
FAS
TNF
SUMO1
TNFRSF1B
PAX3
TNFRSF1A
HRAS
DAXX
FASLG
SP100
RARA
SIRT1
PML
RB1
TP53
PRAM1
CREBBP
0
trait-decreasing effect
trait-increasing effect

Figure S15. NERINE identifies significant rare variant burden in thirteen pathway gene modules in early-onset myocardial infarction (MI) in the UK and MGB biobanks, related to Figure 4.

A. Bonferroni-significant findings for MI across our pathway database of 306 pathways in six functional categories of rare variants—(i) LoF, (ii) damaging missense, (iii) damaging, (iv) missense, (v) neutral missense, and (vi) synonymous (STAR Methods). No inflation was observed in neutral missense or synonymous variant categories. For each variant category, pathways were tested individually in UKBB and MGBBB cohorts, and p-values were meta-analyzed using Fisher's combined test. Negative log-transformed Fisher's combined p-values for significant pathways are shown. The dashed line represents the Bonferroni threshold of 0.05.

B. Network topologies for the pathways with significant rare LoF variant burden in MI with NERINE-predicted gene-level effects, averaged across cohorts. Node color represents effect sizes and directions (orange: trait-increasing; blue: trait-decreasing; intensity reflects magnitude). Pathways are color-coded by the broad groups they belong to (light grey: inflammatory response, light green: extracellular matrix proteins and coagulation, light orange: regulation of transcriptional activity, and light purple: MAPK signaling cascade). We recognize several caveats: the findings around complement system pathways showed disparity among the UKBB and MGBBB. Also, the finding around the MAPK signaling pathway might be due to either true biology or the CHIP (Clonal Hematopoiesis of Indeterminate Potential) effect because the original bio-samples were primarily from blood in MGBBB.

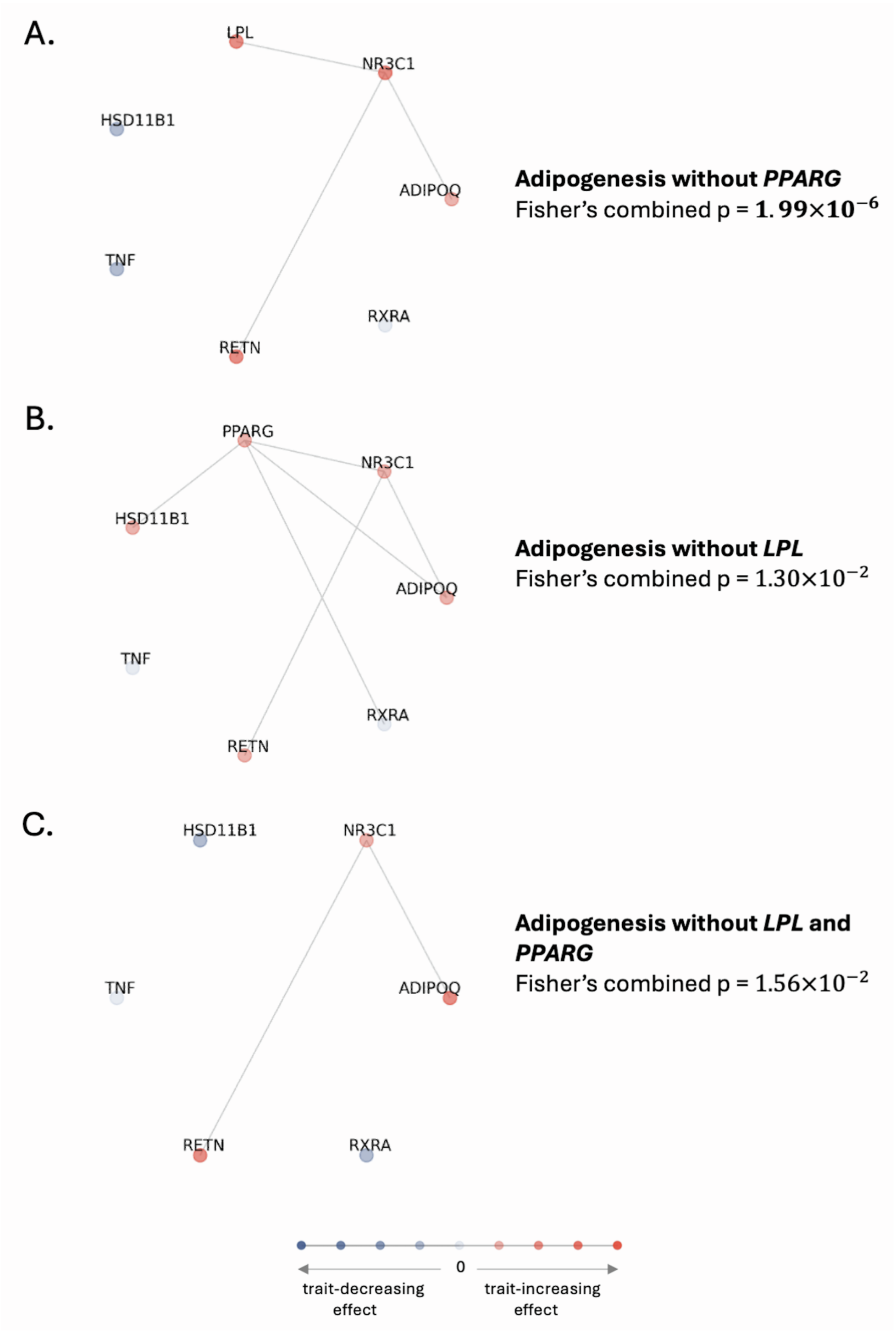
A.
LPL
NR3C1
HSD11B1
ADIPOQ
Adipogenesis without PPARG
Fisher's combined p = 1.99×10^−6
TNF
RXRA
RETN
B.
PPARG
NR3C1
HSD11B1
Adipogenesis without LPL
Fisher's combined p = 1.30×10^−2
ADIPOQ
TNF
RXRA
RETN
C.
HSD11B1
NR3C1
Adipogenesis without LPL and PPARG
Fisher's combined p = 1.56×10^−2
TNF
ADIPOQ
RETN
RXRA
0
trait-decreasing effect
trait-increasing effect

Figure S16. Sensitivity analysis showing NERINE's performance on the *adipogenesis* (BIOCARTA VOBESITY PATHWAY) network for T2D after removing *LPL and PPARG*, related to Figure 4 and STAR Methods.

A. NERINE identifies a significant rare damaging variant burden after removing *PPARG* and its connections from the network.

B. After removing *LPL* and its connections, NERINE identified a nominally significant burden across the rest of the network.

C. Removing both *LPL and PPARG* from the network, NERINE still identifies a nominally significant burden of rare damaging variant burden.

NERINE-predicted gene effects are averaged across UKBB and MGBB cohorts. Node color represents effect sizes and directions (orange: trait-increasing; blue: trait-decreasing; intensity reflects magnitude).

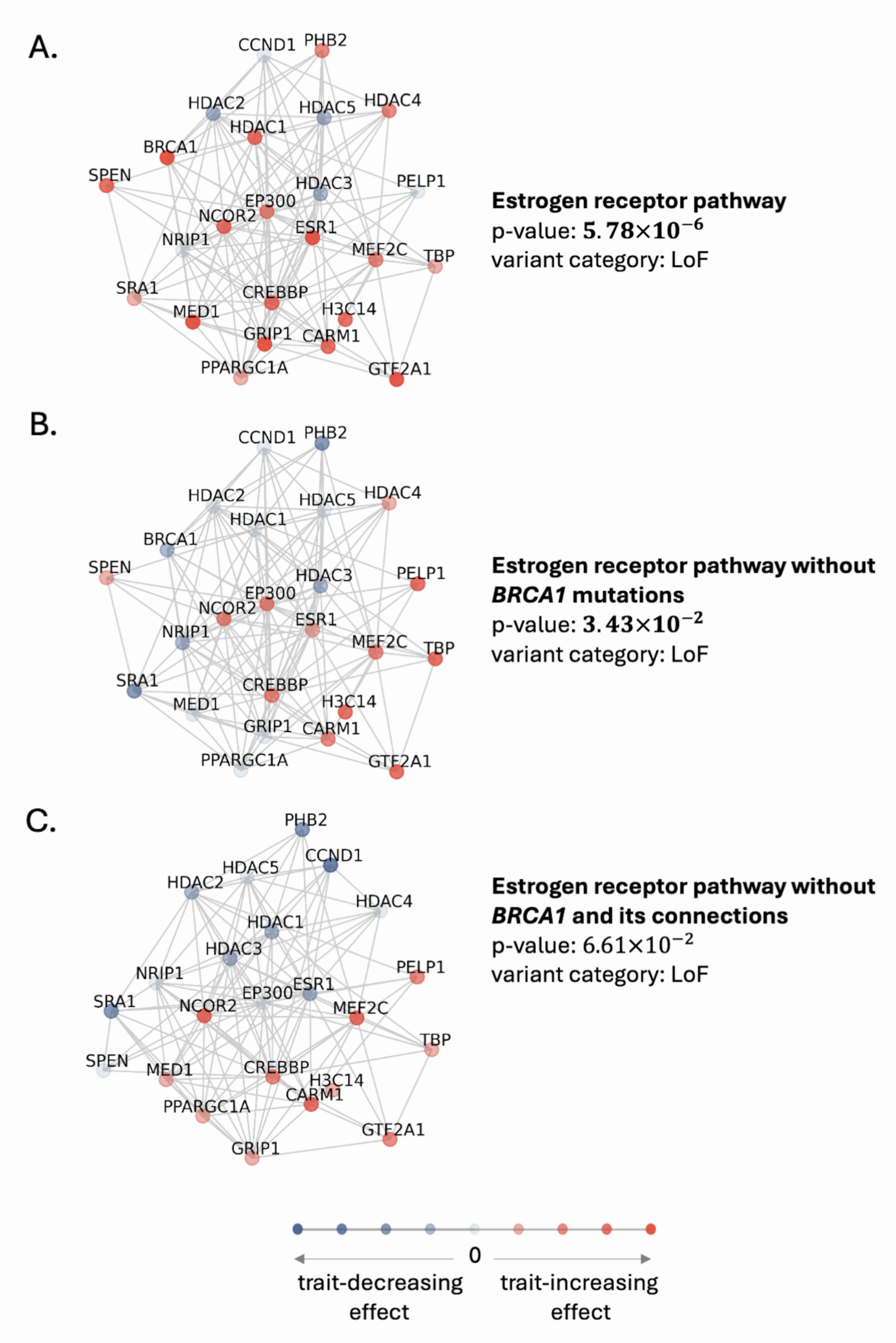
A.
CCND1
PHB2
HDAC2
HDAC5
HDAC4
HDAC1
BRCA1
SPEN
HDAC3
PELP1
EP300
NCOR2
ESR1
NRIP1
MEF2C
TBP
SRA1
CREBBP
MED1
H3C14
GRIP1
CARM1
PPARGC1A
GTF2A1
Estrogen receptor pathway
p-value: 5.78×10^{-6}
variant category: LoF
B.
CCND1
PHB2
HDAC2
HDAC5
HDAC4
HDAC1
BRCA1
SPEN
HDAC3
PELP1
EP300
NCOR2
ESR1
NRIP1
MEF2C
TBP
SRA1
CREBBP
MED1
H3C14
GRIP1
CARM1
PPARGC1A
GTF2A1
Estrogen receptor pathway without *BRCA1* mutations
p-value: 3.43×10^{-2}
variant category: LoF
C.
PHB2
CCND1
HDAC5
HDAC2
HDAC4
HDAC1
HDAC3
NRIP1
PELP1
EP300
ESR1
SRA1
NCOR2
MEF2C
TBP
SPEN
MED1
CREBBP
H3C14
CARM1
PPARGC1A
GTF2A1
GRIP1
Estrogen receptor pathway without *BRCA1* and its connections
p-value: 6.61×10^{-2}
variant category: LoF
0
trait-decreasing effect
trait-increasing effect

Figure S17. Sensitivity analysis showing NERINE's performance on the *regulation of the estrogen receptor* pathway for BRCA in UKBB after removing the effect of *BRCA1*, related to Figure 5 and STAR Methods.

A. NERINE identifies a database-wide significant rare LoF variant burden in the original analysis of the *regulation of the estrogen receptor* network.

B. After removing the observed mutation counts in *BRCA1* but keeping the gene and its connections in the network, NERINE identified a nominally significant burden.

C. NERINE was run after removing the *BRCA1* gene and its edges from the network, and a suggestive rare LoF variant burden was still identified.

Node color represents NERINE-predicted gene effect (orange: trait-increasing; blue: trait-decreasing; intensity reflects magnitude).

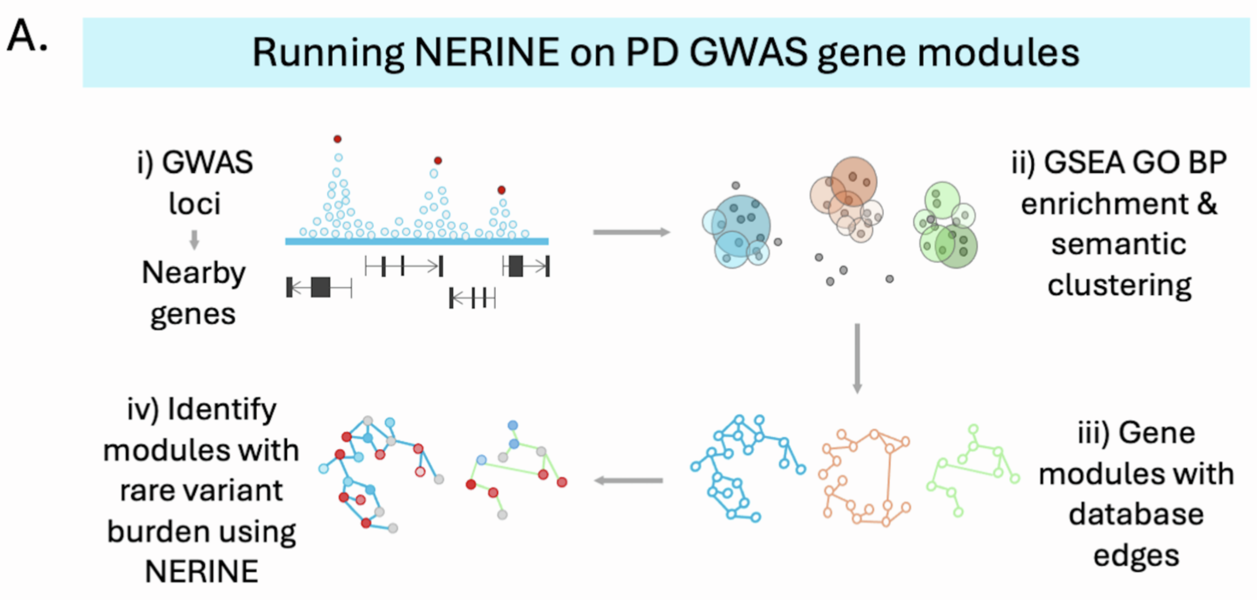

B.

GWAS gene module with significant rare variant burden

peptidyl-threonine modification

Category	Avg. $\hat{\theta}$	Fisher combo-p	Screen-wide Bonf. p
LoF	**0.9**	$\mathbf{7.23\times10^{-3}}$	**0.0434**
Neutral	0	5.97×10^{-1}	1
Synonymous	0	5.97×10^{-1}	1

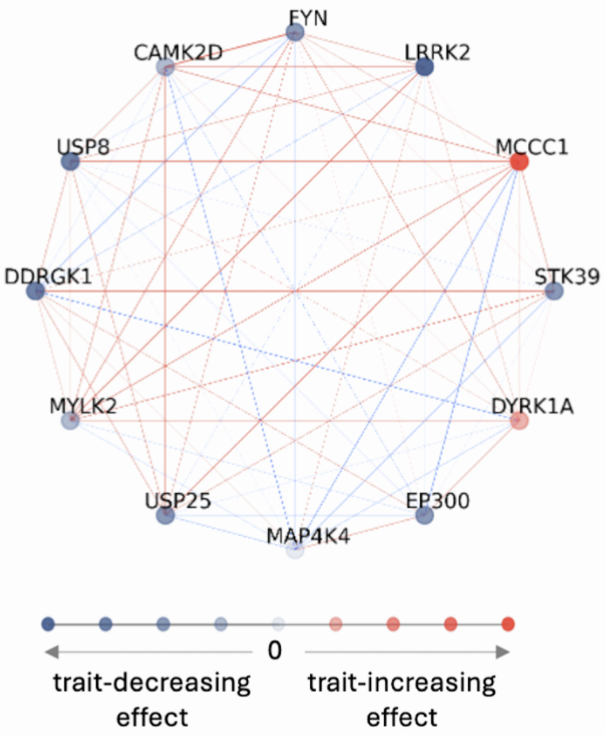

Gene symbol	Known rare variant hit	Role relevant to PD
CAMK2D	X	Synaptic plasticity
DDRGK1	X	Protein homeostasis, inflammation, stress response
DYRK1A	X	Tau phosphorylation, neurodevelopment
EP300	X	Histone acetylation, neuronal survival
FYN	X	Tau phosphorylation, synaptic function
LRRK2	✓	Key risk gene for PD, kinase activity, neuroinflammation
MAP4K4	X	Neuroinflammation, stress response, apoptosis
MCCC1	X	Mitochondrial metabolism
MYLK2	X	Muscle and neuronal cytoskeletal stability
STK39	X	Oxidative stress response, neuroinflammation
USP25	X	Inflammation, ubiquitin signaling, proteostasis
USP8	X	α-synuclein degradation, endosomal trafficking, LRRK2 regulation

Figure S18. NERINE identifies significant rare LoF variant burden in a *peptidyl-threonine modification* network enriched in PD GWAS genes, related to Results and STAR Methods.

A. Network hypotheses generation for interrogating gene modules enriched in PD GWAS genes using NERINE. We identified six GO biological process (BP) modules which were tested with NERINE on both AMP-PD and UKBB sporadic PD vs. control cohorts (STAR Methods).
B. The GO BP module related to *peptidyl-threonine modification* shows significant rare LoF variant (i.e., frameshifts, insertions, deletions, and splice variants) burden across both cohorts. NERINE selected co-essentiality in CNS cell-types as the optimal topology for this module. Screen-wide significance was determined by applying Bonferroni correction over Fisher combined p-values. The absence of enrichment of neutral missense and synonymous variants in the networks served as an internal control. Node color represents the direction of NERINE-predicted gene effect (orange: trait-increasing; blue: trait-decreasing; intensity reflects magnitude). Edge color indicates the sign of the correlation (red: positive; blue: negative), while edge width reflects the correlation strength. Sources of information on network genes: GWAS associations from GWAS catalog (https://www.ebi.ac.uk/gwas/); rare variant associations from Genebass[1], SAIGE-GENE+[2], and PD-specific rare variant studies[26,27]; functional annotations from SynGO (https://www.syngoportal.org/).

A.

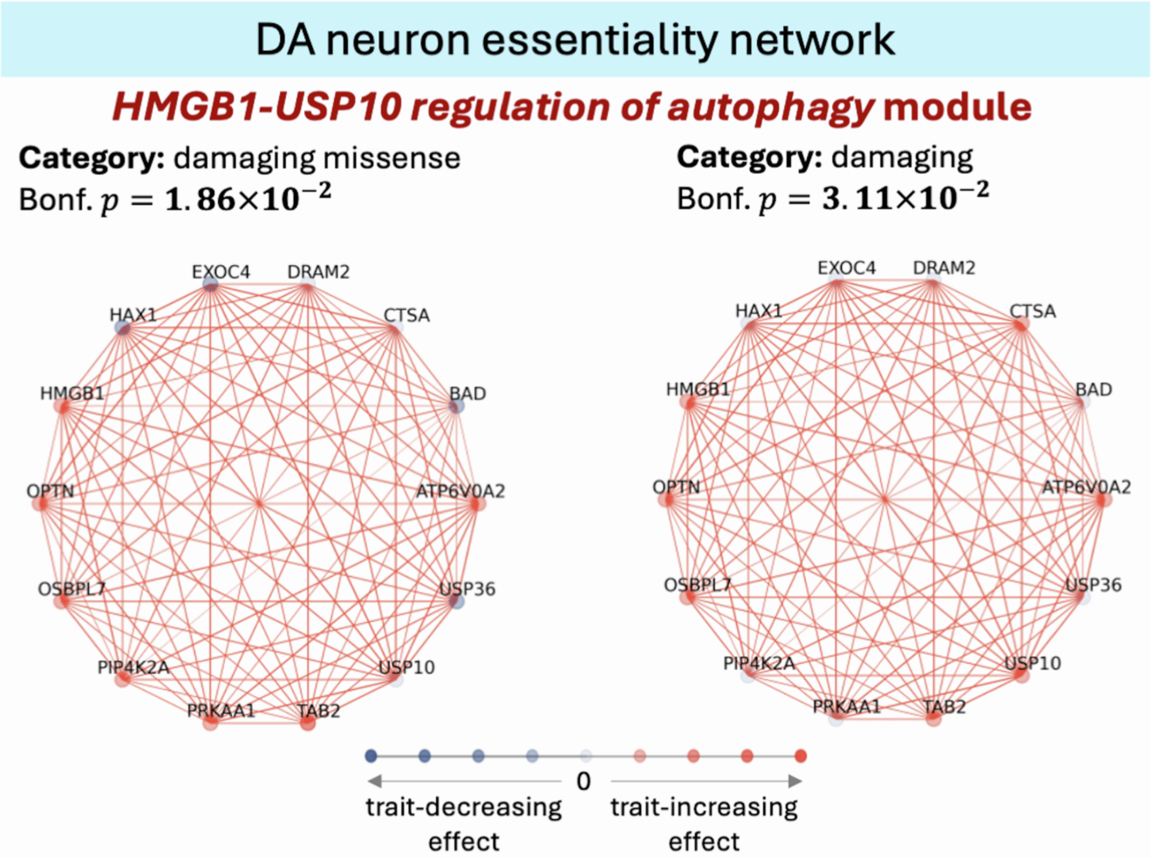

B.

Gene symbol	GWAS hit	Known rare variant hit	Role relevant to PD
ATP6V0A2	X	X	Lysosomal acidification, autophagy
BAD	X	X	Apoptosis regulation, neuronal survival
CTSA	X	X	Lysosomal function, proteostasis
DRAM2	X	X	Autophagy regulation, oxidative stress response
EXOC4	X	X	Synaptic vesicle trafficking
HAX1	X	X	Mitochondrial integrity, cell survival
HMGB1	X	X	Neuroinflammation, immune response
OPTN	X	X	Mitophagy, clearance of damaged mitochondria
OSBPL7	X	X	Lipid metabolism, neuronal membrane homeostasis
PIP4K2A	X	X	Membrane signaling, autophagy regulation
PRKAA1	X	X	Energy metabolism, neuronal stress response
TAB2	X	X	NF-κB signaling, neuroinflammation
USP10	X	X	Ubiquitin signaling, protein degradation
USP36	X	X	Nucleolar protein homeostasis

Figure S19. NERINE's screen-wide significant findings in DA neuron essentiality screen, related to Figure 6.

A. In the DA neuron essentiality screen, NERINE identified screen-wide significant burden of rare damaging missense (left) and damaging (right) variants in the GO biological process (BP) module *regulation of autophagy*. Co-expression in the mid-brain *substantia nigra* region provided the optimal topology for this module. Screen-wide significance of networks was determined by applying Bonferroni correction over the Fisher combined p-values using the number of gene modules tested in the screen ($t_{eff} = 10$). Node color represents the direction of NERINE-predicted gene effect (orange: trait-increasing; purple: trait-decreasing; intensity reflects magnitude). Edge color indicates the sign of the correlation (red: positive; blue: negative), while edge width reflects the correlation strength.

B. Additional information on DA essentiality genes in the *regulation of autophagy* module. Sources of information on network genes: GWAS associations from GWAS catalog (https://www.ebi.ac.uk/gwas/); rare variant associations from Genebass[1], SAIGE-GENE+[2], and PD-specific rare variant studies[3,4]; functional annotations from SynGO (https://www.syngoportal.org/).

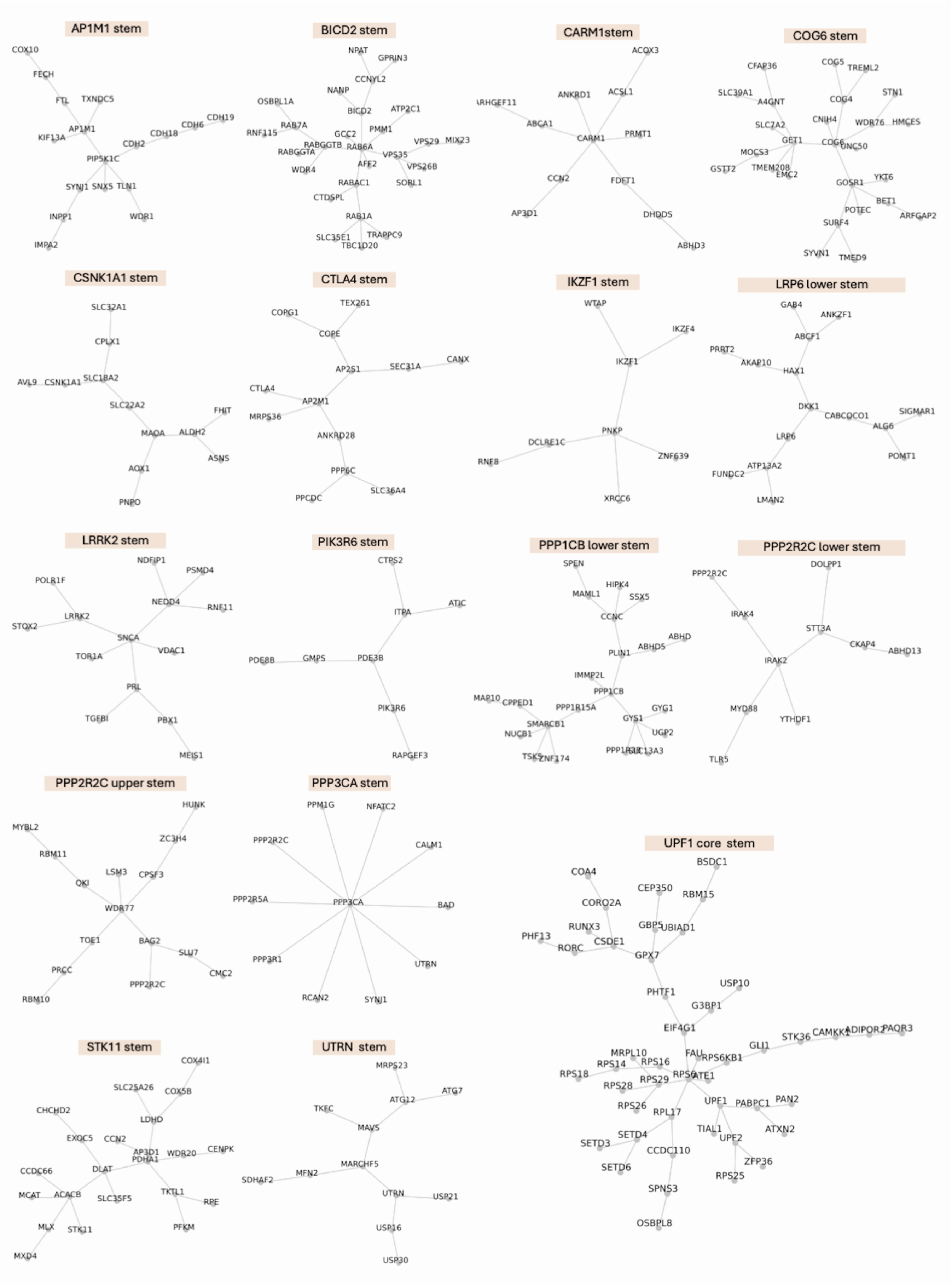

Figure S20. TransposeNet topologies of 17 stems of the humanized *αS*-proteotoxicity network, related to Figure 6.

A.

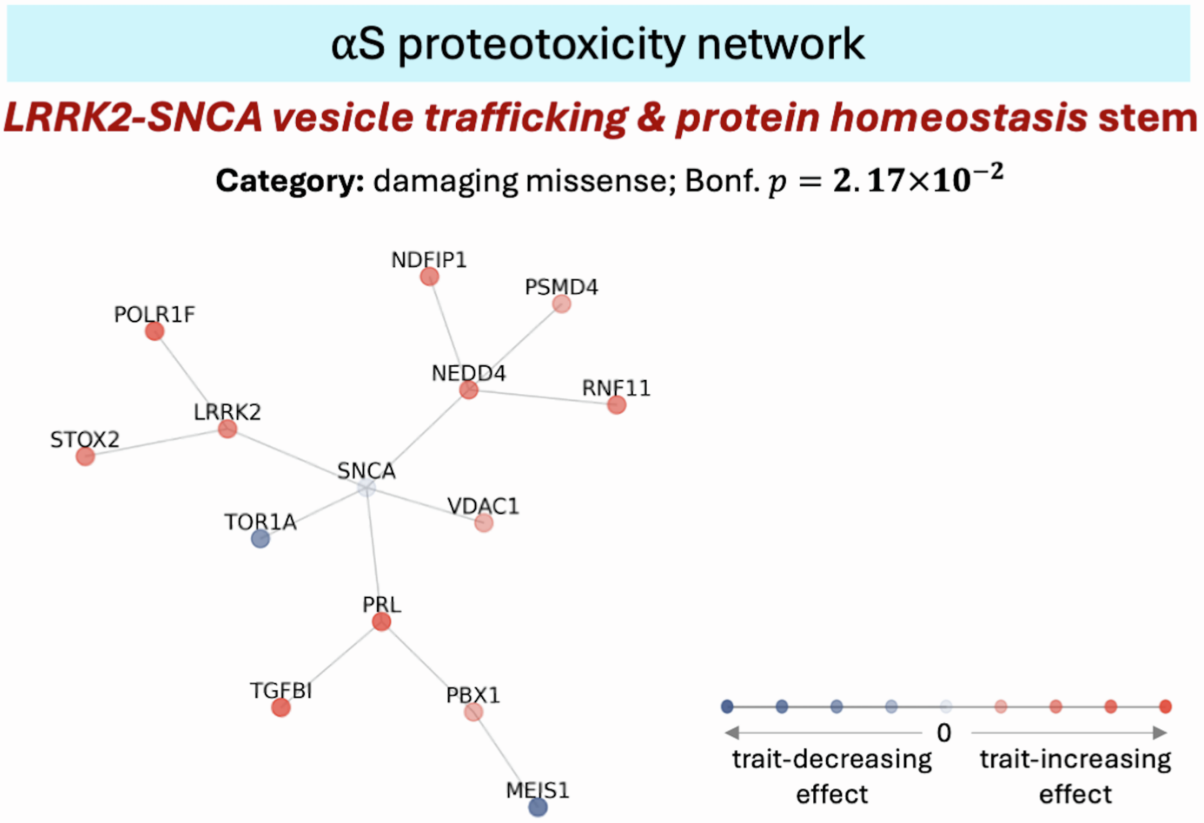

B.

Gene symbol	GWAS hit	Known rare variant hit	Role relevant to PD
PSMD4	X	X	Ubiquitin-proteasome system, protein degradation
NEDD4	X	X	Ubiquitin ligase, α-synuclein degradation
LRRK2	✓	✓	Key PD risk gene, kinase activity, neuroinflammation
SNCA	✓	*	Major component of Lewy bodies, α-synuclein aggregation
MEIS1	X	X	Dopaminergic neuron development
RNF11	X	X	Ubiquitin signaling, neuronal survival
POLR1F	X	X	Ribosomal RNA synthesis, neuroprotection
STOX2	X	X	Neurodevelopment, oxidative stress response
NDFIP1	X	X	protein degradation regulation, α-synuclein clearance
PRL	X	X	Neuroprotective effects, dopamine regulation
TOR1A	X	X	ER-associated protein folding, dystonia
PBX1	X	X	Neural differentiation, dopaminergic neuron maintenance
TGFBI	X	X	Neuroinflammation, extracellular matrix regulation
VDAC1	X	X	Mitochondrial function, apoptosis regulation

Figure S21. NERINE's screen-wide significant finding in the yeast-to-neuron *αS* proteotoxicity screen, related to Figure 6.

A. The *LRRK2- and SNCA*-containing *vesicle trafficking and protein homeostasis*-related subnetwork of αS-modifier genes showed a screen-wide significant burden of rare damaging missense variants in AMP-PD and UKBB datasets. Screen-wide significance of networks was determined by applying Bonferroni correction over the Fisher combined p-values using the number of gene modules tested in the screen (t_{eff} = 17). Here, node color represents the direction of NERINE-predicted gene effect, averaged across cohorts (orange: trait-increasing; purple: trait-decreasing; intensity reflects magnitude). Edges in the TransposeNet module represent binary relationships and are therefore colored in gray.

B. Additional information on *αS*-modifier genes in the *LRRK2-SNCA*-containing *vesicle trafficking and protein homeostasis* stem. Only *LRRK2* had converging signals from common and rare variants, as well as from Mendelian genetics. Although *SNCA* was not a hit in population-based rare variant association tests, linkage studies identified rare Mendelian variants at this locus (as indicated by * in the table). Sources of information on network genes: GWAS associations from GWAS catalog (https://www.ebi.ac.uk/gwas/); rare variant associations from Genebass[1], SAIGE-GENE+[2], and PD-specific rare variant studies[3,4]; functional annotations from SynGO (https://www.syngoportal.org/).

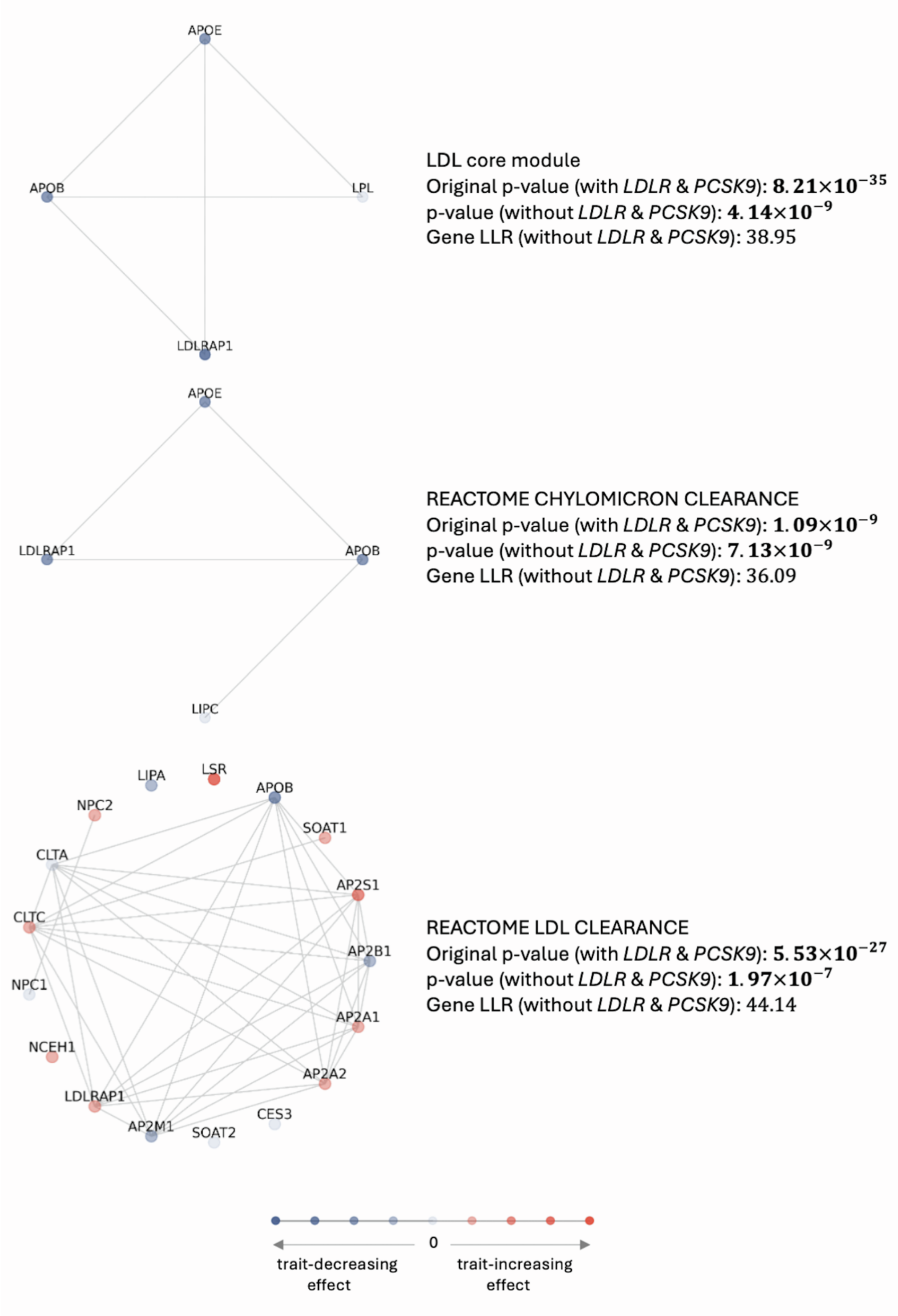

Figure S22. Sensitivity analysis showing lipid-related pathways with significant LoF variant burden identified by NERINE for the high LDL-C vs low LDL-C phenotype in UKBB after removing *LDLR* and *PCSK9*, related to STAR Methods.

Sensitivity analysis was performed by eliminating *LDLR* and *PCSK9* from the networks with significant LoF variant burden for the binarized LDL-C phenotype (high vs low) in UKBB. Despite removing *LDLR* and *PCSK9*, two genes with the largest trait-increasing and trait-decreasing effects on the phenotype, *LDL-core*, *LDL clearance*, and *chylomicron clearance* pathways, remained significant after Bonferroni correction. Here, node color represents the direction of NERINE-predicted gene effect (orange: trait-increasing; purple: trait-decreasing; intensity reflects magnitude).

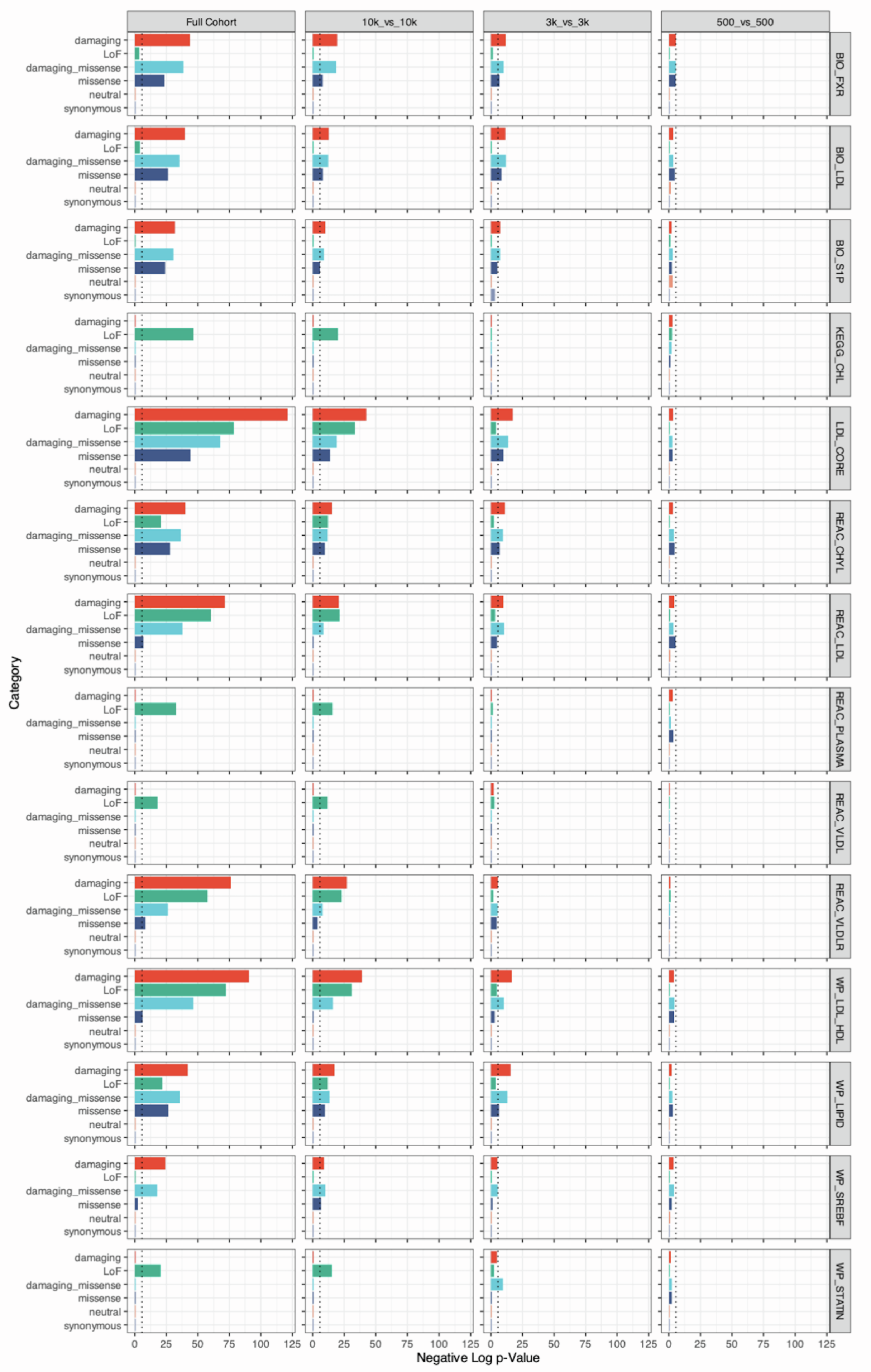

Full Cohort
10k_vs_10k
3k_vs_3k
500_vs_500
BIO_FXR
BIO_LDL
BIO_S1P
KEGG_CHL
LDL_CORE
REAC_CHYL
REAC_LDL
REAC_PLASMA
REAC_VLDL
REAC_VLDLR
WP_LDL_HDL
WP_LIPID
WP_SREBF
WP_STATIN
damaging
LoF
damaging_missense
missense
neutral
synonymous
Category
0
25
50
75
100
125
Negative Log p-Value

Figure S23. Performance of NERINE in full vs downsampled cohorts for the high LDL-C vs low LDL-C phenotype in UKBB, related to STAR Methods.

The high LDL-direct vs. low LDL-direct cohort was downsampled at different case-control ratios (1/3, 1/10, and 1/60). For each downsampled cohort, NERINE was competitively applied across our pathway database of 306 pathways (STAR Methods). We recovered a significant rare damaging variant burden in most of the lipid-related pathways that were significant in the analysis of the original cohort. For a cohort with as few as 500 cases and 500 controls, most of the top pathways showed nominal significance in the functional categories (LoF, damaging, and damaging missense). No inflation was observed in the neutral missense and synonymous categories. The dashed line represents database-wide Bonferroni-corrected p-value cuto4 of 0.05. Here, BIO_FXR = BIOCARTA FXR PATHWAY, BIO_LDL = BIOCARTA LDL PATHWAY, BIO_S1P = BIOCARTA S1P PATHWAY, HDL_CORE = HDL core, KEGG_CHL = KEGG CHOLESTEROL METABOLISM, LDL_CORE = LDL core, REAC_CHYL = REACTOME CHYLOMICRON CLEARANCE, REAC_LDL = REACTOME LDL CLEARANCE, REAC_PLASMA = REACTOME PLASMA LIPOPROTEIN CLEARANCE, REAC_VLDL = REACTOME VLDL CLEARANCE, REAC_VLDLR = REACTOME VLDLR INTERNALISATION AND DEGRADATION, WP_LIPID = WP COMPOSITION OF LIPID PARTICLES, WP_LDL_HDL = WP METABOLIC PATHWAY OF_LDL HDL AND TG INCLUDING DISEASES, WP_SREBF = WP SREBF AND MIR33 IN CHOLESTEROL AND LIPID HOMEOSTASIS, and WP_STATIN = WP STATIN PATHWAY.

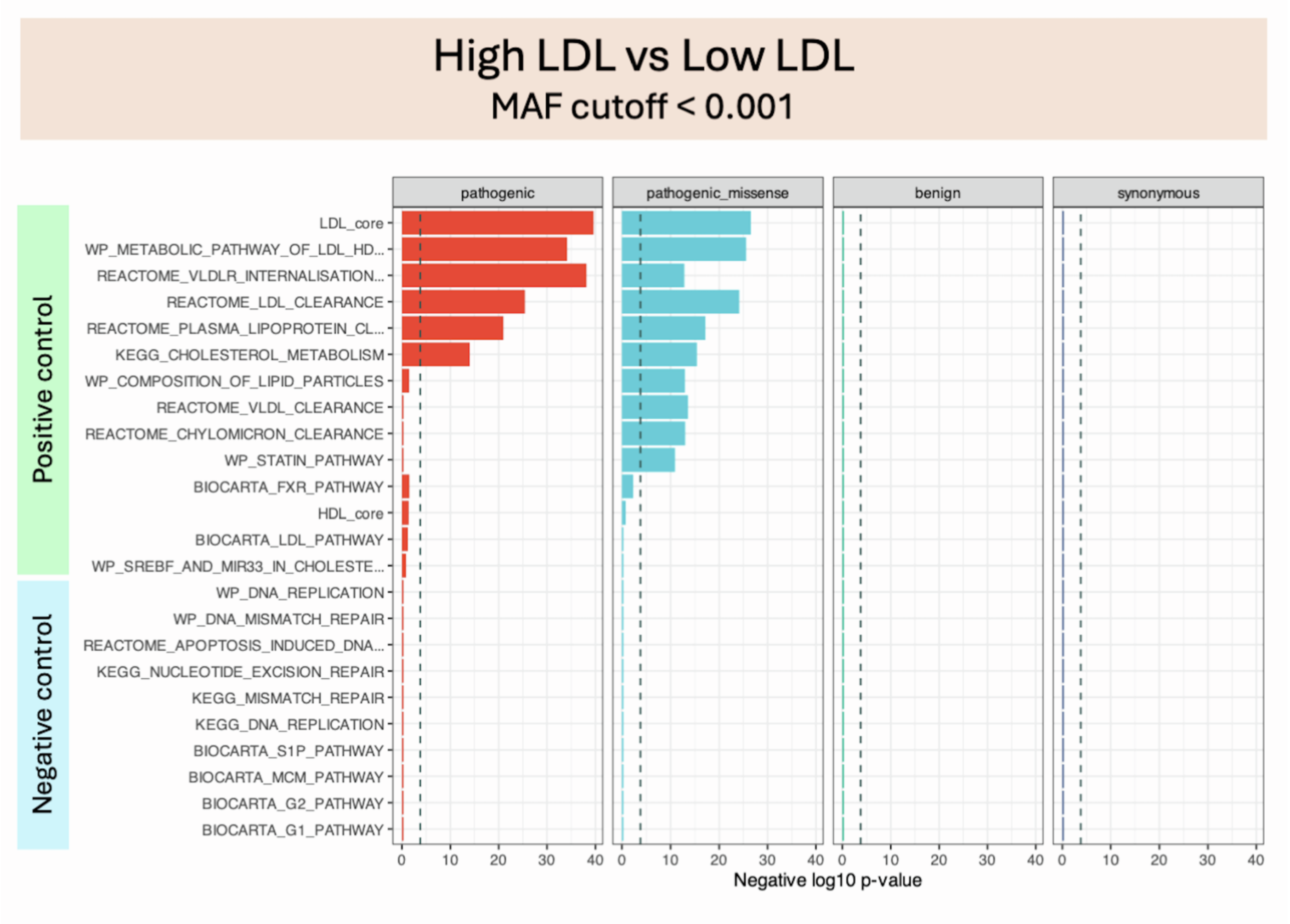

Figure S24. NERINE identifies significant rare variant network burden in functional categories defined by AlphaMissense and REVEL in high LDL-C vs. low LDL-C individuals in the UK Biobank, related to STAR Methods.

For the binarized LDL-C phenotype (high vs low) in UKBB, NERINE identifies significant burden of rare (MAF < 0.001) variants in pathogenic missense (i.e., missense variants with either AlphaMissense score > 0.564 or REVEL score >= 0.664), and pathogenic (i.e., frameshifts, insertions, deletions, splice region variants, and pathogenic missenses) categories in key lipid-related pathways (STAR Methods). The results are very similar to the analysis performed with functional variants identified by in-silico predictors, PolyPhen2 and SIFT. No significant burden of benign missense (i.e., missense variants with either AlphaMissense score < 0.34 or REVEL score < 0.5) and synonymous variants was observed. None of the cell-cycle and DNA damage repair pathways showed an enrichment in the pathogenic categories which served as a negative control. The tests were performed across our database of 306 pathways. Pathway gene lists were extracted from MSigDB (v7.3). High-confidence physical and genetic interactions in protein-protein interaction (PPI) databases were used as network edges between pathway genes (STAR Methods).

Supplementary Tables

Table S1. Qualitative comparison of NERINE's model with existing pathway or gene-set based rare variant tests, related to Figure 1 and STAR Methods.

Method	Variants collapsed at	Incorporate edge geometry	Model	Reference
NERINE	Indiv. genes	✓	Hierarchical parametric model, log-likelihood ratio test	This study
RVTT	Mega-gene	X	Permutation-based trend test on frequency of variants	[5-7]
META-STAAR	Mega-gene	X	Variance-component model	[8]
SAIGE-GENE+	Mega-gene	X	Variance-component model	[2]
PHARAOH-GEE	Indiv. genes	X	Generalized estimating equations-based test	[9]
PHARAOH-multi	Indiv. genes	X	Hierarchical doubly ridge-regularized regression model for multiple phenotypes	[10]
PHARAOH	Indiv. genes	X	Hierarchical doubly ridge-regularized regression model	[11]
TRAPD	Mega-gene	X	Fisher-exact test using gnomAD controls	[12]
SFPCA	Mega-gene	X	Smoothed functional principal component analysis	[13]
aSPUpath	Indiv. genes	X	Adaptive sum of powered scores test	[14]
WKS-pathway	Indiv. genes	X	Weighted Kolmogorov-Smirnov test combining gene-level p-values	[15]
Direct region tests	Mega-gene	X	SKAT, SKAT-O, Fisher-exact applied to mega-gene	[15]

Table S4. Competitively selecting tissue-specific network topologies from GTEx for core HDL-related gene module with NERINE for the binarized HDL-C phenotype (low vs high) in the UK biobank, related to Figure 3.

Network topologies were constructed from gene-gene co-expression in all 52 tissue types in GTEx (v8) for the core HDL gene module (STAR Methods). Each topology was tested with NERINE for rare damaging variants selected with a minor allele frequency (MAF) cutoff of 0.001. NERINE achieved the most significant p-value with the co-expression network constructed from the liver tissue, highlighted in boldface. Here, $\hat{\theta}$ indicates the estimated effect size of the gene network by NERINE and LLR stands for the log-likelihood ratio.

GTEx v8 tissue	NERINE *p*-value	- log10 (*p*)	Bonf. adj. *p*	$\hat{\theta}$	*LLR*
**liver**	**4.48E-76**	**75.35**	**1.34E-73**	0.80	**339.32**
bladder	5.79E-74	73.24	1.74E-71	0.06	329.63
brain substantia nigra	6.35E-74	73.20	1.91E-71	0.20	329.44
brain putamen basal ganglia	6.63E-74	73.18	1.99E-71	0.30	329.36
brain cortex	8.36E-74	73.08	2.51E-71	0.20	328.90
brain cerebellar hemisphere	1.93E-73	72.71	5.80E-71	0.30	327.22
adrenal gland	2.88E-73	72.54	8.65E-71	0.70	326.43
thyroid	3.82E-73	72.42	1.15E-70	0.20	325.87
kidney cortex	4.61E-73	72.34	1.38E-70	0.06	325.49
ovary	5.40E-73	72.27	1.62E-70	0.40	325.17
brain hippocampus	6.01E-73	72.22	1.80E-70	0.20	324.96
vagina	7.79E-73	72.11	2.34E-70	0.30	324.45
small intestine terminal ileum	2.75E-72	71.56	8.24E-70	0.50	321.93
colon transverse	3.03E-72	71.52	9.09E-70	0.06	321.74
colon sigmoid	3.59E-72	71.44	1.08E-69	0.06	321.40
uterus	4.86E-72	71.31	1.46E-69	0.40	320.80
brain caudate basal ganglia	6.78E-72	71.17	2.03E-69	0.30	320.13
spleen	1.08E-71	70.97	3.23E-69	0.06	319.21
minor salivary gland	1.08E-71	70.97	3.25E-69	0.20	319.20
adipose subcutaneous	1.40E-71	70.85	4.19E-69	0.30	318.69
heart left ventricle	2.17E-71	70.66	6.52E-69	0.90	317.81
cervix ectocervix	2.21E-71	70.66	6.63E-69	0.30	317.77
kidney medulla	2.62E-71	70.58	7.85E-69	0.20	317.44
skin not sun exposed suprapubic	3.19E-71	70.50	9.58E-69	0.06	317.04
brain cerebellum	4.27E-71	70.37	1.28E-68	0.90	316.46
prostate	4.60E-71	70.34	1.38E-68	0.50	316.32
heart atrial appendage	6.61E-71	70.18	1.98E-68	0.70	315.59
brain anterior cingulate cortex ba24	8.81E-71	70.06	2.64E-68	0.80	315.02
brain hypothalamus	9.11E-71	70.04	2.73E-68	0.20	314.95
nerve tibial	1.02E-70	69.99	3.05E-68	0.40	314.73
brain spinal cord cervical c-1	1.22E-70	69.92	3.65E-68	0.70	314.38
esophagus gastroesophageal junction	2.87E-70	69.54	8.62E-68	0.20	312.66
cells ebv-transformed lymphocytes	2.90E-70	69.54	8.69E-68	0.30	312.64

Table S4. ***continued.***

GTEx_v8 tissue	NERINE *p*-value	- log10 (*p*)	Bonf. adj. *p*	$\hat{\theta}$	LLR
artery tibial	4.29E-70	69.37	1.29E-67	0.80	311.86
artery aorta	4.92E-70	69.31	1.48E-67	0.40	311.59
adipose visceral omentum	1.03E-69	68.99	3.09E-67	0.40	310.12
esophagus mucosa	1.05E-69	68.98	3.15E-67	0.20	310.08
artery coronary	1.25E-69	68.90	3.74E-67	0.20	309.74
cervix endocervix	1.25E-69	68.90	3.76E-67	0.40	309.72
muscle skeletal	1.32E-69	68.88	3.97E-67	0.60	309.62
cells cultured fibroblasts	1.37E-69	68.86	4.11E-67	0.06	309.55
lung	1.40E-69	68.85	4.19E-67	0.30	309.51
pancreas	1.99E-69	68.70	5.96E-67	0.60	308.81
pituitary	2.42E-69	68.62	7.27E-67	0.20	308.41
brain nucleus accumbens basal ganglia	3.38E-69	68.47	1.01E-66	0.06	307.75
stomach	4.41E-69	68.36	1.32E-66	0.20	307.22
brain amygdala	4.75E-69	68.32	1.42E-66	0.40	307.07
breast mammary tissue	5.12E-69	68.29	1.53E-66	0.60	306.92
fallopian tube	1.44E-68	67.84	4.33E-66	0.50	304.85
brain frontal cortex ba9	3.92E-68	67.41	1.18E-65	0.20	302.86
esophagus muscularis	3.60E-67	66.44	1.08E-64	0.20	298.44
testis	5.81E-67	66.24	1.74E-64	0.50	297.49

Table S6. Pairwise Jaccard similarity and overlap between NERINE-identified database-wide significant pathways for breast cancer (BRCA), related to Figure 4.

The upper triangle shows the number of overlapping genes for each pair of pathways. The lower triangle shows the corresponding Jaccard indices. Number of genes per pathway is shown in parentheses.

	ATM PATHWAY	ATRBRCA PATHWAY	BARD1 PATHWAY	CARM ER PATHWAY	G2 PATHWAY	P53HYPOXIA PATHWAY	PLK3 PATHWAY	Group
**ATM PATHWAY** (20)		9	1	1	8	6	4	Cancer susceptibility
**ATRBRCA PATHWAY** (22)	0.27		7	1	6	2	5	Cancer susceptibility
**BARD1 PATHWAY** (8)	0.04	0.30		1	1	0	0	Cancer susceptibility
**CARM ER PATHWAY** (24)	0.02	0.02	0.03		2	1	0	Regulation of estrogen receptor
**G2 PATHWAY** (24)	0.22	0.15	0.03	0.04		6	7	Cancer susceptibility
**P53HYPOXIA PATHWAY** (20)	0.17	0.05	0.00	0.02	0.15		2	Cancer susceptibility
**PLK3 PATHWAY** (8)	0.17	0.20	0.00	0.00	0.28	0.07		Cancer susceptibility

Table S12. GO biological process modules constructed from PD GWAS genes, related to STAR Methods, Figure S18, and Methods S1.

GO enrichment analysis was performed for PD GWAS genes (STAR Methods). Semantically similar GO BP terms were grouped together to create modules. Modules containing less than 10 genes were filtered out.

Module name	Grouped GO terms	Genes
positive regulation of receptor recycling	GO:0001921; GO:0001919; GO:0002431; GO:0002579; GO:0002691; GO:0002828; GO:0002861; GO:0002862; GO:0003254; GO:0010507; GO:0010656; GO:0010746; GO:0010821; GO:0010975; GO:0019216; GO:0023056; GO:0031111; GO:0031327; GO:0031329; GO:0031340; GO:0031648; GO:0032272; GO:0032489; GO:0033143; GO:0035542; GO:0038094; GO:0043068; GO:0045652; GO:0045747; GO:0045794; GO:0045913; GO:0046579; GO:0050848; GO:0050871; GO:0051052; GO:0051128; GO:0051896; GO:0060159; GO:0060161; GO:0060627; GO:0060628; GO:0060688; GO:0060732; GO:0070431; GO:0080090; GO:0080135; GO:0090043; GO:0090311; GO:0090322; GO:0098815; GO:0098909; GO:0120183; GO:1900034; GO:1900044; GO:1900449; GO:1901077; GO:1901222; GO:1901224; GO:1901897; GO:1902306; GO:1902498; GO:1902950; GO:1903142; GO:1903391; GO:1903421; GO:1903429; GO:1903573; GO:1903719; GO:1903721; GO:1903748; GO:1903859; GO:1903861; GO:1904714; GO:1904889; GO:1905279; GO:2000109; GO:2000147; GO:2000377; GO:2000586	*STK39; RAB29; INPP5F; CHD9; FASN; BAG3; SEMA4A; CLCN3; KPNA1; EP300; GPNMB; RETREG3; NUCKS1; IP6K2; LRRK2; FYN; NSF; USP25; PMVK; FGD4; SNCA; BST1; DDRGK1; TRIM40; NOD2; SETD1A; DYRK1A; CRHR1; RIMS1; GAK; CD19; CAMK2D; HIP1R; MAP4K4; PIK3CA; FCGR2A; SCARB2; GCH1; IRS2; SYT17; RIT2; FGF20*
peptidyl-threonine modification	GO:0018210; GO:0006468; GO:0006475; GO:0006551; GO:0018105; GO:0018107; GO:0018209; GO:0018212; GO:0034205; GO:0046777; GO:0070536; GO:0071108; GO:0071569; GO:1990592	*STK39; MCCC1; LRRK2; FYN; CAMK2D; USP8; DDRGK1; MYLK2; USP25; MAP4K4; EP300; DYRK1A*
dopamine biosynthetic process	GO:0042416; GO:0006661; GO:0006665; GO:0006768; GO:0016310; GO:0032958; GO:0034626; GO:0042559; GO:0043604; GO:0046477; GO:0046949; GO:0090407; GO:1901566	*STK39; INPP5F; FASN; PAM; IP6K2; MCCC1; LRRK2; FYN; SNCA; GALC; SLC44A4; DYRK1A; SPTSSB; ELOVL7; CAMK2D; MAP4K4; PIK3CA; PIGL; GCH1; ITPKB*
membrane organization	GO:0061024; GO:0006996; GO:0007030; GO:0010256; GO:0048312; GO:0050808; GO:0051276; GO:0070841; GO:0070842; GO:0090166; GO:1903008	*RAB29; NUCKS1; VAMP4; LRRK2; SATB1; CHRNB1; FGD4; SNCA; USP8; DDRGK1; SH3GL2; VPS13C; SPTSSB; RIMS1; GBF1; GAK; HIP1R; SCARB2; MTF2; BAG3*

Table S12. ***continued.***

Module name	Grouped GO terms	Genes
protein localization to endoplasmic reticulum	GO:0070972; GO:0006325; GO:0006892; GO:0006895; GO:0006898; GO:0015871; GO:0015917; GO:0016482; GO:0017156; GO:0033365; GO:0034067; GO:0035493; GO:0045053; GO:0048205; GO:0061025; GO:0070973; GO:0072657; GO:0090494; GO:0099504; GO:0099641; GO:1903441; GO:1905383; GO:1990778	*RAB29; INPP5F; DLG2; NUCKS1; VAMP4; LRRK2; KCNIP3; SATB1; NSF; SNCA; DDRGK1; SLC44A4; FAM47E; SH3GL2; VPS13C; RIMS1; GBF1; GAK; HIP1R; MTF2; SCARB2; SYT17*
cellular response to metal ion	GO:0071248; GO:0000165; GO:0006971; GO:0007268; GO:0010042; GO:0019221; GO:0032026; GO:0035864; GO:0035865; GO:0038146; GO:0048015; GO:0062197; GO:0071225; GO:0071476; GO:0071481; GO:0071870; GO:0071871; GO:0071872; GO:1901652; GO:1901653; GO:1990416	*STK39; RAB29; INPP5F; DLG2; NUCKS1; MAP3K14; IP6K2; LRRK2; FYN; NSF; CHRNB1; SNCA; HLA-DRB5; SH3GL2; NOD2; RIMS1; ITPKB; CAMK2D; MAP4K4; PIK3CA; IRS2; SYT17; RIT2; FGF20*

Table S15. GO biological process modules constructed from the hits of genome-wide CRISPR essentiality screen in dopaminergic (DA) neuron, related to Figure 6.
GO enrichment analysis was performed for the essentiality genes that were significantly associated with DA neuron survival in the screen (STAR Methods). Semantically similar GO BP terms were grouped together to create modules. Modules containing less than 10 genes were filtered out.

Module name	Grouped GO terms	Genes
regulation of apoptotic process	GO:0042981	*ANP32E; APH1A; AREL1; ARHGEF18; ARHGEF3; ATF4; BAD; CAPN3; DHRS2; DUSP6; GLS2; HAX1; HIGD2A; HMGB1; HSP90AA1; HSPA1B; HTT; HYPK; IGF1R; IRF5; MOAP1; MRE11; NEUROD1; PHB2; PIK3R1; PIP5KL1; PNMA1; PRDX5; PRKAA1; PSEN2; RASGRF2; RBM5; RNPS1; RRP1B; RTN4; SGK1; TOMM20; TOMM70; TRIM2; USP36; XBP1; ZNF622*
mitochondria	GO:0051204; GO:0006839; GO:0046931; GO:0006626; GO:0007005	*AFDN; ATP2A1; BAD; CAV2; CHCHD10; COX7A2; DNAJC15; HAX1; HIGD2A; HSP90AA1; MOAP1; MSTO1; NDUFA13; NUP205; PHB2; PISD; RTN4; SIRT3; SLC25A5; THG1L; TIMM13; TIMM44; TIMM8B; TMEM14B; TMEM14C; TMEM170A; TOMM20; TOMM70*
positive regulation of programmed cell death	GO:0043068	*APH1A; ARHGEF18; ARHGEF3; ATF4; BAD; DUSP6; HMGB1; HTT; IRF5; MOAP1; NEUROD1; PIP5KL1; PNMA1; PSEN2; RASGRF2; RBM5; RNPS1; RRP1B; ZNF622*
mRNA processing	GO:0044417; GO:0000389; GO:0006397; GO:0051292	*ALYREF; AQR; ATF4; CRNKL1; CWF19L2; ESS2; GTF2F2; HNRNPD; ISY1; NUP205; PHF5A; RBM5; RNPS1; RTN4; SF3A1; SF3B5; THOC2; THOC3; THOC5; THOC6; TMEM170A; ZBTB1*
cellular protein modification process	GO:0006464	*ASB1; ASB16; ASB4; B4GALT7; C3; CCDC8; CDC25B; CDK11A; CDK3; CDK4; CDK8; CTH; DDA1; DUSP10; DUSP6; EIF2AK2; EIF2AK4; FAM20A; FBXL19; FBXL22; FSTL3; GPC3; IKBKE; LOXL1; LOXL2; MACROD1; MAML1; MAST2; NEK1; NEK4; NEURL2; NIM1K; PPM1J; PPP1CC; PRKAA1; PRKCB; PRKCH; PSMA4; PSME2; PSME3; SGK1; SIRT3; SOCS3; STK38L; TULP4; UBE2S; VCAN; VGF*
regulation of autophagy	GO:0010506	*ATP6V0A2; BAD; CTSA; DRAM2; EXOC4; HAX1; HMGB1; OPTN; OSBPL7; PIP4K2A; PRKAA1; TAB2; TAB3; USP10; USP36*
inflammation	GO:0051092; GO:0001819	*ADRA2A; ATF4; C3; CAPN3; CEBPG; CYBA; EGR1; EIF2AK2; HAVCR2; HMGB1; HSPA1B; IRF5; MAP3K7; MBP; MRE11; NFKBIB; PDE4B; PRKCB; PRKCH; PRKCQ; RNF25; TAB2; TAB3; XBP1*
protein catabolic process	GO:0010498; GO:0006511	*AREL1; FBXL19; FBXL22; GID4; HUWE1; KCTD10; NDFIP2; NEDD4L; NEDD9; PSMA4; PSME2; PSME3; TRIM2; TRPC4AP; UBE2S; UBE4B; UBL7; UBXN6; UFD1; XBP1*

Table S15. *continued.*

Module name	Grouped GO terms	Genes
positive regulation of intracellular signal transduction	GO:1902533	*ADRA2A; BAD; CAV2; EIF2AK2; FLT3LG; GDF15; HAX1; HMGB1; HSP90AA1; IFIT5; IGF1R; IKBKE; IRS2; MAP3K7; MAZ; NDFIP2; NR3C2; PHB2; PIK3R1; PRKCB; RTN4; TAB2; TAB3; TMEM9B; TRAF7; TRIP6; ZNF622*
cellular response to DNA damage stimulus	GO:0006974	*CBX3; CDK3; FAAP20; HMCES; IKBKE; MACROD1; MOAP1; MRE11; NEK4; NSMCE3; POLK; RAD51B; RBM24; RECQL4; TIMELESS; USP10; XPA; ZBTB1; ZBTB4*

Table S20. Age breakdown of Parkinson's disease (PD) cohorts, related to Figure 6 and STAR Methods.

	AMP-PD sporadic		UKBB sporadic		UKBB extreme	
**Age at recruitment**	**Case**	**Control**	**Case**	**Control**	**Case**	**Control**
Mean	63.6	62.5	62.9	53.7	62.9	67.2
Median	65	63	64	55	64	67
Stdev	9.1	10.3	5.3	5.4	5.3	1.5
**Median age as of 2024**	Not available	Not available	81	73	81	87

Supplementary Methods

Methods S1. Application of NERINE to GWAS gene modules in Parkinson's Disease (PD), related to STAR Methods, Figure S18, and Tables S12-S14.

To investigate rare variant signal around GWAS-identified loci in Parkinson's disease (PD) and to demonstrate how NERINE can be applied to molecular networks assembled from ontological gene sets to highlight genes and gene modules that are amenable to more focused experimentation, we applied NERINE to six gene ontology (GO) biological process (BP) modules significantly enriched in PD GWAS-associated genes[16-18] (**Figure S18A**, **Table S12**). Network modules were generated by grouping semantically similar GO biological process terms enriched in significant GWAS loci and then extracting the edge relationships of genes in each group from—(i) physical and genetic interaction databases, (ii) co-expression in substantia nigra of mid-brain (GTEx v8), and (iii) co-essentiality in CNS cell lines (DepMap v2023Q2) (**STAR Methods**). We analyzed UKBB-sporadic and AMP-PD cohorts for rare variant burden in six variant categories—(i) loss-of-function (LoF), (ii) damaging missense, (iii) damaging (i.e., damaging missense and LoF), (iv) missense, (v) neutral (benign missense), and (vi) synonymous (**STAR Methods**). Here, LoF variants refer to frameshifts, in-frame insertions and deletions, stop-gained, stop-lost, start-lost, splice acceptors, splice donors, and splice-region variants.

NERINE identified a significant burden of rare LoF variants in the module related to *peptidyl-threonine modification* (avg. $\hat{\theta}$ = 0.9, Bonf. $p = 4.34 \times 10^{-2}$; **Figure S18B**, **Tables S13-S14**), consistent with known kinase-phosphatase dysregulation in PD[19,20]. Co-essentiality in cells from the central nervous system (CNS) was selected as the optimal topology for this module. Within this module, NERINE suggested trait-increasing LoF burden in *MCCC1* and *DYRK1A*, concordant with their effect on mitochondrial dysfunction[21] and DA neuron degeneration[22-24], and trait-decreasing LoF effects in *FYN* and *USP8*, concordant with preclinical evidence that their inhibition protected DA neurons[22-24] and reduced *α-synuclein* (*αS*) accumulation[25,26]. These findings highlighted candidate genes for further functional investigation. For *LRRK2*, NERINE suggested a trait-decreasing effect of LoF variants, a pattern directionally consistent with preclinical studies pointing to potential benefits of *LRRK2* inhibition in PD[27,28]. Notably, however, prior genetic studies of PD found the evidence for the protective role of *LRRK2* haploinsufficiency inconclusive[29], underscoring the need for further experimental investigation.

We also explored an alternative strategy for constructing gene modules from GWAS signals. We utilized the results from the recent multi-ancestry PD GWAS[17], which applied MAGMA[30] gene set analysis and GO enrichment to identify 21 significant, conditionally independent GO BP modules (**Table S23**). For each module, NERINE was competitively applied with network topologies created from PPI, co-expression, and co-essentiality databases to test the six categories of rare variants as described above. This analysis yielded only nominally significant burdens using co-expression networks: damaging and damaging missense variants in the *regulation of neuronal action potential*, damaging variants in *microglial cell proliferation* and *macrophage proliferation*, and LoF variants in the *response to mitochondrial depolarization* module. None of the modules were significant after Bonferroni correction. A summary of these results is provided in **Table S23**.

Methods S2. Conditional probability of allele counts in genes in a network, related to Figure 1 and STAR Methods.

Let, the observed allele counts in gene i in cases and controls be represented by two independent Poisson random variables, X_i and Y_i, respectively, with rate parameters λ_{case}^{i} and $\lambda_{control}^{i}$. The total allele counts for gene i across the cohort is therefore distributed as $X_i + Y_i \sim Poisson(\lambda_{case}^{i} + \lambda_{control}^{i})$. The conditional probability of observing $X_i = k$ alleles in cases, given the total allele count $X_i + Y_i = n$, follows a Binomial distribution with success probability (p) being proportional to the ratio, $\frac{\lambda_{case}^{i}}{\lambda_{case}^{i}+\lambda_{control}^{i}}$. We assume p to be a function of the gene effect α_i in our model.

$$
\begin{aligned}
P(X_i = k | X_i + Y_i = n) &= \frac{P(X_i = k) \times P(Y_i = n-k)}{P(X_i + Y_i = n)} \\
&= \frac{e^{-\lambda_{case}^{i}}(\lambda_{case}^{i})^{k}}{k!} \times \frac{e^{-\lambda_{control}^{i}}(\lambda_{control}^{i})^{(n-k)}}{(n-k)!} \times \frac{n!}{e^{-(\lambda_{case}^{i}+\lambda_{control}^{i})}\left(\lambda_{case}^{i} + \lambda_{control}^{i}\right)^{n}} \\
&= \binom{n}{k}\left(\frac{\lambda_{case}^{i}}{\lambda_{case}^{i} + \lambda_{control}^{i}}\right)^{k}\left(\frac{\lambda_{control}^{i}}{\lambda_{case}^{i} + \lambda_{control}^{i}}\right)^{n-k} = Binom\left(k, n, \frac{\lambda_{case}^{i}}{\lambda_{case}^{i} + \lambda_{control}^{i}}\right) \\
&\approx Binom(n, p = \phi(\alpha_i))
\end{aligned}
$$

A more appropriate modeling strategy would treat X_i and Y_i as independent Binomial random variables. However, under that formulation, the sum $X_i + Y_i$, no longer follows a Binomial distribution[31], and the conditional probability $P(X_i | X_i + Y_i)$ does not have a closed-form expression. Consequently, evaluating NERINE's likelihood function would require computationally intensive moment-based approximations[32], substantially increasing resource requirements and runtime. For this reason, the Poisson-based formulation described above is adopted in NERINE, providing a practical and computationally efficient approximation while preserving essential probabilistic structure.

Methods S3. Custom Beta transformation for gene effects, related to Figure 1 and STAR Methods.

NERINE models the effects of genes within a network, denoted by vector $\vec{\alpha}$, as drawn from a multivariate skew-normal distribution $\vec{\alpha} \sim MSN(0, \theta \cdot \Sigma, \nu)$, where $\nu = f(N_{case}, N_{control})$. The univariate marginals follow skew-normal distributions on $(-\infty, \infty)$. When $\nu = 0$, the model reduces to a multivariate normal distribution with univariate normal marginals. Since we use each α_i as a proxy for the success probability parameter of a binomial distribution approximating the conditional probability of case allele counts for gene i in the cohort given the total allele counts $(P(X_i = k | X_i + Y_i = n) \sim Binom(k, n, p = \phi(\alpha_i))$, we require a transformation that maps $\vec{\alpha}$ to the interval $[0,1]$.

This transformation must satisfy two conditions:

1. The mean of the transformed distribution equals $N_{case}/(N_{case} + N_{control})$.

2. The shape of the transformed distribution adapts with θ: small θs (close to 0) concentrate density near the mean, while large θs shift density toward the extremes, allowing stronger gene effects with an increasingly high probability.

To achieve this, we apply a custom transformation from skew-normal to Beta distributions, rather than a probit transformation. The probit mapping, even under balanced cohorts, distorts local density around the mean, which is critical for lookup table-based likelihood calculations. Moreover, in imbalanced case-control settings, the standard probit approach is not applicable. Although skewed probit regression could theoretically achieve the mapping, it is computationally more complex involving moment-based approximations.

Let's denote the transformed gene effects by vector $\overrightarrow{\alpha'}$, where case-control imbalance is incorporated via the shape parameters of the Beta distribution. For simplicity, let's first start with a balanced case-control cohort; the univariate skew-normal marginals reduce to a scalar normals, $\alpha_i \sim Normal(\mu = 0, \sigma^2)$. In this case, we assume the transformed gene effects are denoted by $\alpha'_i \sim Beta(a, b)$. We find a mapping, $\phi: \alpha_{i_{(-\infty,\infty)}} \rightarrow \alpha'_{i_{[0,1]}}$ preserving the ordering of data points so that $(\alpha_i)_p < (\alpha_i)_q \Longrightarrow (\alpha'_i)_p < (\alpha'_i)_q$:

$$\alpha'_i = F_{\alpha'_i}^{-1}\left(\Phi\left(\frac{\alpha_i - \mu}{\sigma}\right)\right) = F_{\alpha'_i}^{-1}\left(\Phi\left(\frac{\alpha_i}{\sigma}\right)\right)$$

Here, $F_{\alpha'_i}$ is the cumulative distribution function (CDF) of α'_i and Φ is the standard normal CDF. In the balanced case, the Beta mean is 0.5, implying $a = b$. For $a > 1$, the distribution is bell-shaped; for $a < 1$, it is U-shaped, both symmetric around 0.5. These two cases correspond to:

- small θ (close to 0): raw gene effects, α_is, cluster near zero.
- large θ: raw gene effects, α_is, shift toward extremes.

To parameterize this dependence, we set both a and b proportional to the reciprocal of θ. Case-control imbalance is incorporated by setting $a \propto \frac{N_{case}}{N_{control}} b$. In NERINE's current implementation, we set:

$$a = \frac{N_{case}}{(N_{case}+N_{control})\times\theta}, \text{ and } b = \frac{N_{case}\times N_{control}}{N_{case}\times(N_{case}+N_{control})\times\theta}.$$

Figure S25 illustrates the custom transformation in a two-genes example, with gene effects modeled by a bivariate skew-normal distribution $\vec{\alpha} \sim BSN(0, \theta \cdot \Sigma, \nu)$. The mapping correctly shifts the mean gene effect to 0.5 in balanced cohorts, to 0.75 when cases outnumber controls 3:1, and to 0.25 when controls outnumber cases 3:1. This design also separates the treatment of skew from the lookup tables, allowing their efficient reuse in likelihood calculations.

Finally, **Figure S26** compares our custom Beta transformation with the standard probit approach under a balanced two-gene cohort ($\theta = 0.1, \nu = 0, \Sigma = \boldsymbol{I}$). Our method preserves the bell-shaped structure and local density of the marginals, while the probit mapping distorts both, making it unsuitable for NERINE's marginal likelihood calculations.

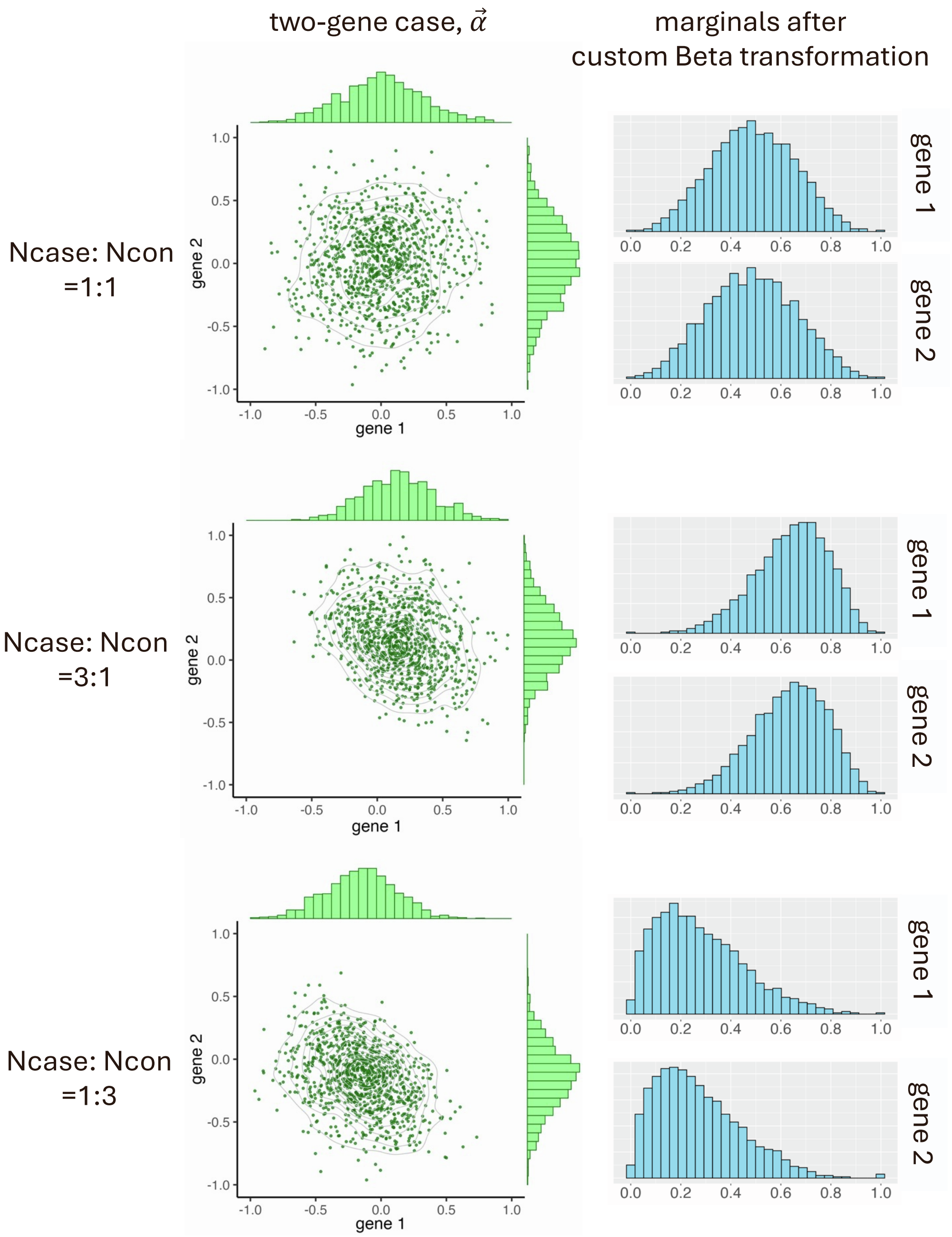

Figure S25. NERINE's custom Beta transformation of gene-effect marginals (two-genes case), related to STAR Methods and Methods S3.

Gene effects, $\vec{\alpha}$ are drawn from a $\mathrm{BSN}(0, \theta \cdot \Sigma, \nu)$, with $\theta = 0.1$, $\Sigma = \boldsymbol{I}$, and $\nu = 3\frac{N_{case}-N_{control}}{N_{case}+N_{control}}$. Both balanced and imbalanced cohorts with left and right skews are shown.

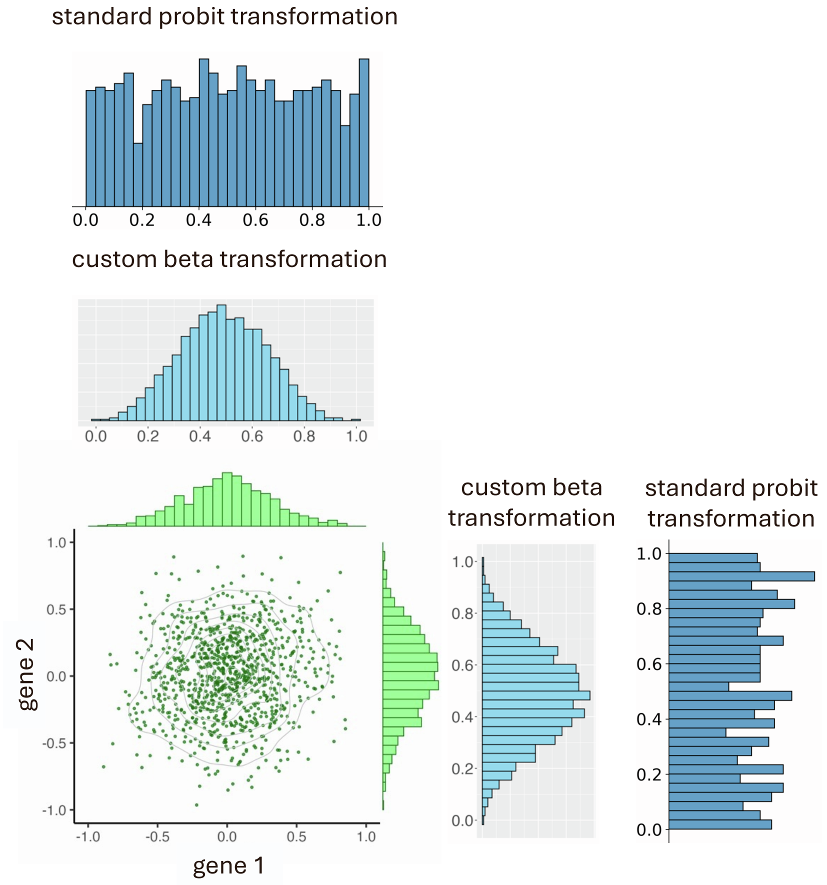

Figure S26. Comparing our custom beta transformation against standard probit transformation in two-genes case, related to STAR Methods and Methods S3.
For gene effects $\vec{\alpha}$ drawn from $\mathrm{BVN}(0, \theta \cdot \Sigma)$, with $\theta = 0.1$ and $\Sigma = \boldsymbol{I}$, our transformation preserves the desired properties of shape and local density needed for NERINE's likelihood calculation. In contrast, the probit transformation (computed using `scipy.stats.norm.cdf` with a mean of 0 and scale of $\sqrt{0.1}$) distorts the local densities, and is therefore unsuitable for our purposes.

Methods S4. Computing the likelihood of network effect (*L*) using a lookup table with pruning, related to Figure 1 and STAR Methods.

NERINE infers the network-effect (θ) on a dichotomous phenotype using the maximum likelihood estimation (MLE) framework, where the likelihood is given by,

$$L(\theta|\boldsymbol{X}, \mathbf{Y}, \vec{\alpha}, \Sigma, N_{case}, N_{control}) = \int \left(\prod_{i=1}^{m} P(X_i|X_i + Y_i, \alpha_i) \right) P(\vec{\alpha}|\theta; \Sigma, \nu) d\vec{\alpha}$$

, where $\nu = f(N_{case}, N_{control})$.

We approximate this integral as a weighted sum over *K*-dimensional quadrature points in the domain of integration:

$$L(\theta|\boldsymbol{X}, \mathbf{Y}, \vec{\alpha}, \Sigma, N_{case}, N_{control}) \approx \sum_{\vec{\alpha}} \left(\prod_{i=1}^{m} P(X_i|X_i + Y_i, \alpha_i) \right) P(\vec{\alpha}|\theta; \Sigma, \nu)$$

The weight of each K-variate quadrature point is given by the product of the corresponding univariate weights, effectively sampling the function over a K-dimensional grid. Each quadrature point involves computing two terms. For the first term, the conditional probability of case allele counts in each gene can be approximated using the probability density function of a standard Binomial distribution as described in Methods S1. To calculate the probability of network-gene effects, $\vec{\alpha}$, for a given network topology (Σ) of m genes and network effect ($\theta = \theta_z$), $P(\vec{\alpha}|\theta; \Sigma, \nu)$, we use a lookup table approach with pruning.

In our general framework, $\vec{\alpha} \sim MSN(0, \theta \cdot \Sigma, \nu)$. When $\nu = 0$, $\vec{\alpha}$ follows a multivariate normal (MVN) distribution with mean 0 and univariate normal marginals. For this distribution we can adopt the Gauss-Hermite (GH) quadrature[33] approach. Here, $\theta = \theta_z = 0$ implies each gene-effect α_i is zero. For $\theta = \theta_z > 0$, we first sample N=10,000 points from $\vec{\alpha} \sim MVN(0, \theta_z \cdot \Sigma)$ and apply variable transformation as described in Methods S2 to bound the marginals between 0 and 1. The transformed gene effects are denoted by $\overrightarrow{\alpha'}$. To keep the size of the lookup table tractable we impose a $1 \times K$ grid on each α_i'. This implicitly achieves the effect of applying Cholesky decomposition on our sampled set of points. For the one-directional version of NERINE (i.e., genes can have only trait-increasing effects), we set $K = 4$. For the bi-directional version (i.e., genes can contribute to either increasing or decreasing the trait), we set $K = 9$. From the sampled points, we determine the weightings of the combinations of α_i's. We prune α_i' combinations with extremely low weight (i.e. weight < 1e-4). After the transformation, the approximate likelihood is given by,

$$L \approx \sum_{\overrightarrow{\alpha'}} \left(\prod_{i=1}^{m} P(X_i | X_i + Y_i, \alpha'_i) \right) P\left(\overrightarrow{\alpha'} | \theta; \Sigma, \nu\right)$$

We sample θ_zs from a modified log-linear scale such that we have more θ_zs with very small values close to zero (0) and sparsely distributed samples of θ_z as we move towards larger values. Since very large network effects (i.e., θ >1) are unlikely in practice, we restrict our search on the interval $[0, 1]$. Thus, the final size of the lookup table is set at $|\theta_z| \times K$, where $|\theta_z|$ represents the number of sampled θ_zs. We can extend this multivariate quadrature setup for an MVN (balanced cohort) to compute the general MSN expectations almost for free by reweighting the MVN nodes[34] based on the skew parameter ν. In our case, the reweighting is effectively achieved through the custom Beta transformation of the marginals as described in Methods S2. This approximation scheme performs reasonably well in practice.

Methods S5. Detailed steps for variant and sample quality control in WGS and WES data sets, related to STAR Methods.

For each dataset, we retained high-quality biallelic variants passing GATK best practices filters and having maximum 10% missingness. For the UKBB dataset, variant-level pre-processing was performed on the DNAnexus platform. Only variants with AQ >= 50 were considered as high-quality. For MGBBB and AMP-PD datasets, variants having depth of coverage (DP) at least 10 and mapping quality (MQ) at least 90 were included. All the variant calls were based on the GRCh38 assembly. We annotated the variants with the gnomAD minor allele frequencies (genome AF: gnomADv3 and exome AF: lifted over gnomADv2 exome AFs) and in-silico predictions of deleteriousness of the missense variants by PolyPhen2 and SIFT from the dbNSFP (v4.3a) database using the using VEP (v109). Variants termed as synonymous, missense, splice

donor, splice acceptor, splice region, stop-gained, stop-lost, start-lost, frameshift, in-frame insertion, and in-frame deletion, were included in the analysis. We used six masks to group variants into functional categories: (i) Damaging missense: missense variants predicted to be either “P’’ or “D’’ by PolyPhen2 or “deleterious’’ by SIFT, (ii) LoF: variants labelled as splice donors, splice acceptors, splice region variants, stop-gained, stop-lost, start-lost, frameshifts, in-frame insertions, and in-frame deletions; (iii) Damaging: LoFs and damaging missenses, (iv) Missense, (v) Neutral: missense variants predicted to be either “B’’ by PolyPhen2 or “tolerated’’ by SIFT, and (vi) Synonymous.

Relatedness of individuals was calculated using *King* (v2.3.2) on all variants with MAF > 0.01. All individuals marked as related by King were excluded from our analyses. We performed ancestry analysis of the individuals with the first five genetic principal components using the *somalier* (v0.2.16) tool. Our analyses primarily focused on individuals of European ancestry. Additional sample outliers were removed based on Ts/Tv, Het/Hom ratios, and per-haploid SNV counts. Outliers were defined as samples which are +/- 3 standard deviations away from the mean. We examined the distributions of ultra-rare variants such as singletons (bi-allelic SNPs for which the alternative allele is observed exactly once in the population), doubletons (bi-allelic SNPs for which the alternative allele is observed only twice in the population) and tripletons (bi-allelic SNPs for which the alternative allele is observed only thrice in the population) in all the retained samples to ensure that their distribution follows the binomial expectation. This test enables us to examine whether the distribution of ultra-rare alleles in the case-control cohorts is driven by the underlying structure of the data. We detected no differences when evaluating the distribution of doubletons and tripletons in the AMP-PD dataset. AMP-PD showed highly inflated counts of singletons in PD cases; therefore, we excluded singletons from our analysis of the AMP-PD cohort.

Methods S6. Quantification code of immunostaining data, related to Figure 7 and STAR Methods.

Images were analyzed using ImageJ Macro Software. Source code is provided below. Program 1 was used for analyzing all *prolactin* immunostaining images of CiS neurons at DIV7. Program 2 was used for analyzing the images at DIV28.

<u>Program 1:</u>

```
//READ THE FOLLOWING BEFORE USE:
//This macro will analyze the images and output the number of cells, and the green intensity

run("Colors...", "foreground=white background=black selection=yellow");
run("Options...", "iterations=1 count=1 black");
run("Set Measurements...", "area mean standard min integrated area_fraction redirect=None
decimal=5");

var width, height, pixelscale;
var cellroi, range=5, flag=0;;
var dapi_ch=1, cell_ch=1, green_ch=2;    //you can change the channels here
var T_upper_limit=220, exclude_percent=3, min_cell_size=1000;
var value;
var name;
```

```
var Intensity;
rawdir=getDirectory("User Choose Raw Data Folder");
resultdir=getDirectory("User Choose Result Data Folder");
list=getFileList(rawdir);

print("RawFolder: "+rawdir);
print("Neighbor Range:  "+range);
print("dapiCh     cellCh      punctaCh");
print(dapi_ch+"   "+cell_ch);

print("File name  cellROI#    Total Intensity   Total Intensity/Cell Number");


for(f=0;f<list.length;f++)
 {
  run("Bio-Formats Windowless Importer", "open=["+rawdir+list[f]+"]");

  //get single focused image
  focusimage();

  //width=getWidth(); height=getHeight();
  //get positive cell outline
  selectWindow("Log");
  saveAs("Text", resultdir+ "Summary" +name+ ".csv");

 }

selectWindow("Log"); run("Close");
beep();

//get depth focus image
function focusimage()
 {
   getDimensions(width, height, channels, slices, frames);
   getPixelSize(unit, pixelscale, pixelscale);
   run("Split Channels");

     selectImage("C"+1+"-"+list[f]);
     run("Z Project...", "projection=[Max Intensity]");
     saveAs("Tiff", resultdir+list[f]+"_focusch"+1+".tif"); rename("ch"+1);
      cell();
      selectImage("C"+1+"-"+list[f]); close();
      selectImage("C"+2+"-"+list[f]);
     run("Z Project...", "projection=[Max Intensity]");
     saveAs("Tiff", resultdir+list[f]+"_focusch"+2+".tif"); rename("ch"+2);
     Intensityquant();
   run("Close All");
 }
```

```
function cell()
 {
  // get dapi ROI
  selectImage("ch1");

  run("Duplicate...", "title=ch1_copy.tif");
  run("Enhance Contrast", "saturated=0.50");
  setOption("ScaleConversions", true);
  run("8-bit");
  //run("Subtract Background...", "rolling=50");
  //run("Auto Threshold", "method=MaxEntropy white");
  //run("Auto Threshold", "method=Li white");
  //run("Auto Threshold", "method=Default white");
  //run("Auto Threshold", "method=Intermodes white"); //for clumping
  run("Auto Threshold", "method=Huang2 white");
  //run("Threshold...");
  //setThreshold(51, 255);
  setOption("BlackBackground", true);
  run("Convert to Mask");
  //run("Fill Holes");
  run("Watershed");
  run("Analyze Particles...", "size=100-Infinity circularity=0.1-2.00 clear add");

  if (roiManager("Count") > 0){
  roiManager("Save", resultdir+list[f]+"_dapiROI.zip");
  cellroi=roiManager("Count");
  selectImage("ch1_copy.tif"); close();
  roiManager("Show None");
  }
  //get strong DAPI/dead cell ROI
  selectImage("ch1");
  run("Duplicate...", "title=[ch"+dapi_ch+" strong]");
  run("Enhance Contrast", "saturated=0.50");
  run("8-bit");
  run("Subtract Background...", "rolling=50");
  setThreshold(T_upper_limit, 255);
  setOption("BlackBackground", true);
  run("Convert to Mask");

  //get cell positive
  selectImage("ch"+dapi_ch+" strong");
  run("Clear Results");
  roiManager("Measure"); count=roiManager("Count");
  tmp=0;

  for(i=0;i<count;i++)
   {
```

```
    if( getResult("%Area",i) >= exclude_percent )
     {
      roiManager("Select", i-tmp);
      roiManager("Delete");
      tmp++;
     }
   }
  run("Clear Results");
  value=roiManager("count");

 if (roiManager("Count") > 0){
      roiManager("Save", resultdir+list[f]+"_cellROI.zip");
      cellroi=roiManager("Count");
      selectImage("ch"+dapi_ch+" strong"); close();
}
      roiManager("reset");
 }


function Intensityquant(){
      selectImage("ch2");
      setOption("ScaleConversions", true);
      run("8-bit");
      run("Threshold...");
      setThreshold(20, 250, "raw");
      run("Convert to Mask");
      run("Watershed");
      run("Analyze Particles...", "size=50-Infinity circularity=0-1.00 clear add");
      if (roiManager("Count") > 0){
      roiManager("Save", resultdir+list[f]+"_intensityroi.zip");
            }
      roiManager("reset");
      open(resultdir+list[f]+"_focusch2.tif");
      if( File.exists(resultdir+list[f]+"_IntensityROI.zip")){
            roiManager("Open", resultdir+list[f]+"_Intensityroi.zip");
            roiManager("Measure");
      }
      roiManager("Measure");
      if(isOpen("Results")){
            selectWindow("Results");
            saveAs("Text", resultdir+list[f]+"_Intensity.csv");
            }
      Intensity=0;

      for(row=0; row<nResults; row++)
      {
      Intensity = Intensity + getResult("IntDen", row);
      roiManager("reset");
```

```
}
      name=list[f];
      print(list[f]+"  "+cellroi+"  "+Intensity+"     "+Intensity/cellroi);
      run("Clear Results"); run("Close All");

}
```

<u>Program 2:</u>

Convert NDN2 Files to OmeTiff:

```
//This macro opens High Content .nd2 file, and split it into individual .tiff files
//Output: Individual Z-stack images

rawdir=getDirectory("User Choose Individual Data Folder");

run("Bio-Formats Macro Extensions");
file = File.openDialog("Choose raw .nd2 file");
Ext.setId(file);
Ext.getSeriesCount(seriesCount);
name=File.getName(file);

for(j=1;j<seriesCount;j++)
{
            run("Bio-Formats Importer", "open=["+file+"] color_mode=Default rois_import=[ROI manager] view=Hyperstack stack_order=XYCZT series_"+j);
            run("Bio-Formats Exporter", "save=["+rawdir+"/"+name+"series_"+j+".ome.tif] compression=Uncompressed");
            close();
 }
```

Find live cell count and green intensity:

```
// raw data .nd2 three channel, zstack
// dapi channel : max projection collect ROI#
// green channel : ave projection  intensity for whole frame

run("Colors...", "foreground=white background=black selection=yellow");
run("Options...", "iterations=1 count=1 black");
run("Set Measurements...", "area mean min integrated redirect=None decimal=5");

var width, height, pixelscale;
var cellroi, punctaroi, neighborroi, range, flag=0, minsize, maxsize, nuclei_method, puncta_method, rollingball;
var dapi_ch=1, cell_ch=2, puncta_ch=3;
var min_cell_size, cir;
var width, height, pixelscale;
var g_Int, r_Int, puncta_size, puncta_Int, frame_size, cell_size;
```

```
rawdir=getDirectory("User Choose Raw Data Folder");
resultdir=getDirectory("User Choose Result Data Folder");
list=getFileList(rawdir);

parameter_input();

print("RawFolder: "+rawdir);

print(minsize+"   "+maxsize+" "+min_cell_size+" "+nuclei_method+" "+rollingball+"
      "+cir);
print("dapiCh     cellCh");
print(dapi_ch+"   "+cell_ch); print("");
print("File FrameSize   cellROI#    CellSize    GreenInt    RedInt");
print("dapiCh     cellCh");
print(dapi_ch+"   "+cell_ch); print("");
print("File FrameSize   cellROI#    CellSize    GreenInt    RedInt");

selectWindow("Log");
saveAs("Text", resultdir+"Summary.xls");
selectWindow("Log"); run("Close");


for(f=0;f<list.length;f++)
 {
  run("Bio-Formats Importer", "open=["+rawdir+list[f]+"] color_mode=Default
rois_import=[ROI manager] view=Hyperstack stack_order=XYCZT");
  getDimensions(width, height, channels, slices, frames);
  getPixelSize(unit, pixelscale, pixelscale);

  // create maximum projection of each channel
  run("Split Channels");
  selectWindow("C"+dapi_ch+"-"+list[f]); run("Z Project...", "projection=[Max
Intensity]"); saveAs("Tiff", resultdir+list[f]+"_ch1max.tif"); rename("ch1max");
run("Enhance Contrast", "saturated=0.35");
  selectWindow("C"+cell_ch+"-"+list[f]); run("Z Project...", "projection=[Average
Intensity]"); saveAs("Tiff", resultdir+list[f]+"_ch2ave.tif"); rename("ch2ave");
run("Enhance Contrast", "saturated=0.35");

  selectWindow("C"+dapi_ch+"-"+list[f]); close();
  selectWindow("C"+cell_ch+"-"+list[f]); close();

  //width=getWidth(); height=getHeight();
  //get positive cell outline
  cell();

  // measure green
  selectWindow("ch"+cell_ch+"ave"); run("Measure");
  frame_size = getResult("Area", 0); g_Int = getResult("IntDen", 0);
```

```
  // append measurement
  //print("File   FrameSize   cellRO#     Puncta#     PunctaSize  PunctaInt   GreenInt");
  string = list[f]+"    "+frame_size+"    "+cellroi+" "+cell_size+"     "+g_Int+"
      "+r_Int;
  File.append(string, resultdir+"Summary.xls");

  run("Close All"); roiManager("reset"); run("Clear Results");
 }

roiManager("reset");
run("Clear Results");
print("MACRO FINISHED!!!");

function parameter_input()
 {
  Dialog.create("Parameter_input");
  Dialog.addNumber("Min Cell Size:", 200);
  Dialog.addChoice("Nuclei Threshold Type:", newArray("Huang dark", "Otsu dark", "Default
dark", "Triangle dark", "Yen dark", "Sahnbhag dark", "Intermodes dark", "IsoData dark",
"Li dark", "MaxEntropy dark", "Mean dark", "MinError dark", "Minimum dark", "Moments dark",
"Percentile dark", "RenyEntropy dark" ));
  Dialog.addNumber("RollingBall Radius (pixel):", 50);
  Dialog.addNumber("Circularity:", 0.6);

  Dialog.show();

  min_cell_size = Dialog.getNumber();
  minsize = Dialog.getNumber();
  maxsize = Dialog.getNumber();
  nuclei_method = Dialog.getChoice();
  puncta_method = Dialog.getChoice();
  rollingball = Dialog.getNumber();
  cir = Dialog.getNumber();
 }


function cell()
 {
  // get dapi ROI
  selectImage("ch"+dapi_ch+"max");

  run("Duplicate...", "title=[ch"+dapi_ch+" copy]");
  run("Enhance Contrast", "saturated=0.35");
  run("8-bit");
  run("Gaussian Blur...", "sigma=1");
  run("Threshold...");
  setThreshold(59, 231, "raw");
```

```
  setOption("BlackBackground", true);
  run("Convert to Mask");
  run("Watershed");

  run("Analyze Particles...", "size="+min_cell_size+"-Infinity pixel circularity="+cir+"-
1.00 exclude clear add");
  cellroi = roiManager("Count"); cell_size=0;
  if( cellroi > 0 )
   {
    roiManager("Save", resultdir+list[f]+"_dapiROI.zip");
    roiManager("Measure");
    for(i=0; i< cellroi; i++)
     {
      cell_size = cell_size + getResult("Area",i);
     }
   }
  selectImage("ch"+dapi_ch+" copy"); close();
 roiManager("Show None"); roiManager("reset");
  run("Clear Results");
 }
```

Supplementary References

1. Karczewski, K.J., Solomonson, M., Chao, K.R., Goodrich, J.K., Tiao, G., Lu, W., Riley-Gillis, B.M., Tsai, E.A., Kim, H.I., Zheng, X., et al. (2022). Systematic single-variant and gene-based association testing of thousands of phenotypes in 394,841 UK Biobank exomes. Cell Genom *2*, 100168. 10.1016/j.xgen.2022.100168.
2. Zhou, W., Bi, W., Zhao, Z., Dey, K.K., Jagadeesh, K.A., Karczewski, K.J., Daly, M.J., Neale, B.M., and Lee, S. (2022). SAIGE-GENE+ improves the efficiency and accuracy of set-based rare variant association tests. Nat Genet *54*, 1466-1469. 10.1038/s41588-022-01178-w.
3. Pitz, V., Makarious, M.B., Bandres-Ciga, S., Iwaki, H., andMe Research, T., Singleton, A.B., Nalls, M., Heilbron, K., and Blauwendraat, C. (2024). Analysis of rare Parkinson's disease variants in millions of people. NPJ Parkinsons Dis *10*, 11. 10.1038/s41531-023-00608-8.
4. Makarious, M.B., Lake, J., Pitz, V., Ye Fu, A., Guidubaldi, J.L., Solsberg, C.W., Bandres-Ciga, S., Leonard, H.L., Kim, J.J., Billingsley, K.J., et al. (2023). Large-scale rare variant burden testing in Parkinson's disease. Brain *146*, 4622-4632. 10.1093/brain/awad214.
5. Bendapudi, P.K., Nazeen, S., Ryu, J., Soylemez, O., Robbins, A., Rouaisnel, B., O'Neil, J.K., Pokhriyal, R., Yang, M., Colling, M., et al. (2024). Low-frequency inherited complement receptor variants are associated with purpura fulminans. Blood *143*, 1032-1044. 10.1182/blood.2023021231.
6. Hallacli, E., Kayatekin, C., Nazeen, S., Wang, X.H., Sheinkopf, Z., Sathyakumar, S., Sarkar, S., Jiang, X., Dong, X., Di Maio, R., et al. (2022). The Parkinson's disease protein alpha-synuclein is a modulator of processing bodies and mRNA stability. Cell *185*, 2035-2056 e2033. 10.1016/j.cell.2022.05.008.
7. Bendapudi, P.K., Nazeen, S., Ryu, J., Söylemez, O., Rouaisnel, B., Colling, M., Pasko, B., Robbins, A., Bouzinier, M., Tomczak, L., et al. (2022). Pathway-based Rare Variant Burden Analysis Identifies a Role for the Complement System in an Extreme Phenotype of Sepsis with Coagulopathy. medRxiv, 2022.2002.2024.22271459. 10.1101/2022.02.24.22271459.
8. Li, X., Quick, C., Zhou, H., Gaynor, S.M., Liu, Y., Chen, H., Selvaraj, M.S., Sun, R., Dey, R., Arnett, D.K., et al. (2023). Powerful, scalable and resource-efficient meta-analysis of rare variant associations in large whole genome sequencing studies. Nat Genet *55*, 154-164. 10.1038/s41588-022-01225-6.
9. Lee, S., Kim, S., Kim, Y., Oh, B., Hwang, H., and Park, T. (2019). Pathway analysis of rare variants for the clustered phenotypes by using hierarchical structured components analysis. BMC Med Genomics *12*, 100. 10.1186/s12920-019-0517-4.
10. Lee, S., Kim, Y., Choi, S., Hwang, H., and Park, T. (2018). Pathway-based approach using hierarchical components of rare variants to analyze multiple phenotypes. BMC Bioinformatics *19*, 79. 10.1186/s12859-018-2066-9.
11. Lee, S., Choi, S., Kim, Y.J., Kim, B.J., Consortium, T.d.-G., Hwang, H., and Park, T. (2016). Pathway-based approach using hierarchical components of collapsed rare variants. Bioinformatics *32*, i586-i594. 10.1093/bioinformatics/btw425.
12. Guo, M.H., Plummer, L., Chan, Y.M., Hirschhorn, J.N., and Lippincott, M.F. (2018). Burden Testing of Rare Variants Identified through Exome Sequencing via Publicly Available Control Data. Am J Hum Genet *103*, 522-534. 10.1016/j.ajhg.2018.08.016.
13. Zhao, J., Zhu, Y., Boerwinkle, E., and Xiong, M. (2015). Pathway analysis with next-generation sequencing data. Eur J Hum Genet *23*, 507-515. 10.1038/ejhg.2014.121.
14. Pan, W., Kwak, I.Y., and Wei, P. (2015). A Powerful Pathway-Based Adaptive Test for Genetic Association with Common or Rare Variants. Am J Hum Genet *97*, 86-98. 10.1016/j.ajhg.2015.05.018.
15. Wu, G., and Zhi, D. (2013). Pathway-based approaches for sequencing-based genome-wide association studies. Genet Epidemiol *37*, 478-494. 10.1002/gepi.21728.

16. Nalls, M.A., Blauwendraat, C., Vallerga, C.L., Heilbron, K., Bandres-Ciga, S., Chang, D., Tan, M., Kia, D.A., Noyce, A.J., Xue, A., et al. (2019). Identification of novel risk loci, causal insights, and heritable risk for Parkinson's disease: a meta-analysis of genome-wide association studies. Lancet Neurol *18*, 1091-1102. 10.1016/S1474-4422(19)30320-5.
17. Kim, J.J., Vitale, D., Otani, D.V., Lian, M.M., Heilbron, K., andMe Research, T., Iwaki, H., Lake, J., Solsberg, C.W., Leonard, H., et al. (2024). Multi-ancestry genome-wide association meta-analysis of Parkinson's disease. Nat Genet *56*, 27-36. 10.1038/s41588-023-01584-8.
18. Foo, J.N., Chew, E.G.Y., Chung, S.J., Peng, R., Blauwendraat, C., Nalls, M.A., Mok, K.Y., Satake, W., Toda, T., Chao, Y., et al. (2020). Identification of Risk Loci for Parkinson Disease in Asians and Comparison of Risk Between Asians and Europeans: A Genome-Wide Association Study. JAMA Neurol *77*, 746-754. 10.1001/jamaneurol.2020.0428.
19. Gitler, A.D., Chesi, A., Geddie, M.L., Strathearn, K.E., Hamamichi, S., Hill, K.J., Caldwell, K.A., Caldwell, G.A., Cooper, A.A., Rochet, J.C., and Lindquist, S. (2009). Alpha-synuclein is part of a diverse and highly conserved interaction network that includes PARK9 and manganese toxicity. Nat Genet *41*, 308-315. 10.1038/ng.300.
20. Dzamko, N., Zhou, J., Huang, Y., and Halliday, G.M. (2014). Parkinson's disease-implicated kinases in the brain; insights into disease pathogenesis. Front Mol Neurosci *7*, 57. 10.3389/fnmol.2014.00057.
21. Barallobre, M.J., Perier, C., Bove, J., Laguna, A., Delabar, J.M., Vila, M., and Arbones, M.L. (2014). DYRK1A promotes dopaminergic neuron survival in the developing brain and in a mouse model of Parkinson's disease. Cell Death Dis *5*, e1289. 10.1038/cddis.2014.253.
22. Saminathan, H., Ghosh, A., Zhang, D., Song, C., Jin, H., Anantharam, V., Kanthasamy, A., and Kanthasamy, A.G. (2021). Fyn Kinase-Mediated PKCdelta Y311 Phosphorylation Induces Dopaminergic Degeneration in Cell Culture and Animal Models: Implications for the Identification of a New Pharmacological Target for Parkinson's Disease. Front Pharmacol *12*, 631375. 10.3389/fphar.2021.631375.
23. Panicker, N., Sarkar, S., Harischandra, D.S., Neal, M., Kam, T.I., Jin, H., Saminathan, H., Langley, M., Charli, A., Samidurai, M., et al. (2019). Fyn kinase regulates misfolded alpha-synuclein uptake and NLRP3 inflammasome activation in microglia. J Exp Med *216*, 1411-1430. 10.1084/jem.20182191.
24. Guglietti, B., Carr, L., Ellul, B., Mustafa, S., Corrigan, F., and Collins-Praino, L.E. (2021). Fyn kinase inhibition using AZD0530 improves recognition memory and reduces depressive-like behaviour in an experimental model of Parkinson’s disease. bioRxiv, 2021.2006.2016.448746. 10.1101/2021.06.16.448746.
25. Alexopoulou, Z., Lang, J., Perrett, R.M., Elschami, M., Hurry, M.E., Kim, H.T., Mazaraki, D., Szabo, A., Kessler, B.M., Goldberg, A.L., et al. (2016). Deubiquitinase Usp8 regulates alpha-synuclein clearance and modifies its toxicity in Lewy body disease. Proc Natl Acad Sci U S A *113*, E4688-4697. 10.1073/pnas.1523597113.
26. Mauri, S., Bernardo, G., Martinez, A., Favaro, M., Trevisan, M., Cobraiville, G., Fillet, M., Caicci, F., Whitworth, A.J., and Ziviani, E. (2023). USP8 Down-Regulation Promotes Parkin-Independent Mitophagy in the Drosophila Brain and in Human Neurons. Cells *12*. 10.3390/cells12081143.
27. Lee, B.D., Shin, J.H., VanKampen, J., Petrucelli, L., West, A.B., Ko, H.S., Lee, Y.I., Maguire-Zeiss, K.A., Bowers, W.J., Federoff, H.J., et al. (2010). Inhibitors of leucine-rich repeat kinase-2 protect against models of Parkinson's disease. Nat Med *16*, 998-1000. 10.1038/nm.2199.
28. Taymans, J.M., Fell, M., Greenamyre, T., Hirst, W.D., Mamais, A., Padmanabhan, S., Peter, I., Rideout, H., and Thaler, A. (2023). Perspective on the current state of the LRRK2 field. NPJ Parkinsons Dis *9*, 104. 10.1038/s41531-023-00544-7.
29. Blauwendraat, C., Reed, X., Kia, D.A., Gan-Or, Z., Lesage, S., Pihlstrom, L., Guerreiro, R., Gibbs, J.R., Sabir, M., Ahmed, S., et al. (2018). Frequency of Loss of Function Variants in LRRK2 in Parkinson Disease. JAMA Neurol *75*, 1416-1422. 10.1001/jamaneurol.2018.1885.

30. de Leeuw, C.A., Mooij, J.M., Heskes, T., and Posthuma, D. (2015). MAGMA: generalized gene-set analysis of GWAS data. PLoS Comput Biol *11*, e1004219. 10.1371/journal.pcbi.1004219.
31. Butler, K., and Stephens, M.A. (2017). The distribution of a sum of independent binomial random variables. Methodology and Computing in Applied Probability *19*, 557-571.
32. Liu, B., and Quertermous, T. (2017). Approximating the sum of independent non-identical binomial random variables. arXiv preprint arXiv:1712.01410.
33. Jäckel, P. (2005). A note on multivariate Gauss-Hermite quadrature. London: ABN-Amro. Re.
34. Gupta, A.K., González-Farıas, G., and Domınguez-Molina, J.A. (2004). A multivariate skew normal distribution. Journal of multivariate analysis *89*, 181-190.
